# Long-Term Patterns in the Population Dynamics of *Daphnia longispina*, *Leptodora kindtii* and Cyanobacteria in a Shallow Reservoir: A Self-Organising Map (SOM) Approach

**DOI:** 10.1371/journal.pone.0144109

**Published:** 2015-12-03

**Authors:** Adrianna Wojtal-Frankiewicz, Andrzej Kruk, Piotr Frankiewicz, Zuzanna Oleksińska, Katarzyna Izydorczyk

**Affiliations:** 1 Department of Applied Ecology, Faculty of Biology and Environmental Protection, University of Lodz, Lodz, Poland; 2 Department of Ecology and Vertebrate Zoology, Faculty of Biology and Environmental Protection, University of Lodz, Lodz, Poland; 3 European Regional Centre for Ecohydrology, Polish Academy of Science, Lodz, Poland; National Taiwan University, TAIWAN

## Abstract

The recognition of long-term patterns in the seasonal dynamics of *Daphnia longispina*, *Leptodora kindtii* and cyanobacteria is dependent upon their interactions, the water temperature and the hydrological conditions, which were all investigated between 1999 and 2008 in the lowland Sulejow Reservoir. The biomass of cyanobacteria, densities of *D*. *longispina* and *L*. *kindtii*, concentration of chlorophyll *a* and water temperature were assessed weekly from April to October at three sampling stations along the longitudinal reservoir axis. The retention time was calculated using data on the actual water inflow and reservoir volume. A self-organising map (SOM) was used due to high interannual variability in the studied parameters and their often non-linear relationships. Classification of the SOM output neurons into three clusters that grouped the sampling terms with similar biotic states allowed identification of the crucial abiotic factors responsible for the seasonal sequence of events: cluster CL-ExSp (extreme/spring) corresponded to hydrologically unstable cold periods (mostly spring) with extreme values and highly variable abiotic factors, which made abiotic control of the biota dominant; cluster CL-StSm (stable/summer) was associated with ordinary late spring and summer and was characterised by stable non-extreme abiotic conditions, which made biotic interactions more important; and the cluster CL-ExSm (extreme/summer), was associated with late spring/summer and characterised by thermal or hydrological extremes, which weakened the role of biotic factors. The significance of the differences between the SOM sub-clusters was verified by Kruskal-Wallis and *post-hoc* Dunn tests. The importance of the temperature and hydrological regimes as the key plankton-regulating factors in the dam reservoir, as shown by the SOM, was confirmed by the results of canonical correlation analyses (CCA) of each cluster. The demonstrated significance of hydrology in seasonal plankton dynamics complements the widely accepted pattern proposed by the plankton succession model for lakes, the PEG (Plankton Ecology Group), and may be useful for the formulation of management decisions in dam reservoirs.

## Introduction

Dam reservoirs are formed and modified by human activity for specific purposes, and they therefore have different hydrological characteristics and thermal and chemical regimes compared to natural lakes. The specific features of reservoirs, especially unstable hydrological conditions, determine the species composition and dynamics of the plankton communities inhabiting them [1]. In this study, the long-term patterns in the seasonal dynamics of three types of planktonic organisms were investigated, dependent upon their interactions, the water temperature and the hydrological alterations. The study was conducted in the lowland dam of the Sulejow Reservoir, which is regarded as a model ecosystem for ecological research due to its location and characteristics [2]. We examined 1) the dominant species of daphniid in this ecosystem, *Daphnia longispina* (O.F. Müller); 2) the predatory cladoceran *Leptodora kindtii* (Focke); and 3) cyanobacteria, which are a serious problem in the reservoir due to their creation of toxic blooms.

The importance of daphniids in ecosystem functioning is related not only to their effects on the abundance, species composition and size structure of phytoplankton [3] but also to bacterial and protistan dynamics [4, 5] and thus to the epilimnetic cycle of organic matter and nutrient balance [6]. The relatively well-known biology and ecology of daphniids (e.g., feeding behaviour, reproduction and both predator-prey and host-parasite interactions) underlie their particular suitability for studies on interactions between trophic levels and on the propagation of trophic effects in aquatic ecosystems [7, 8]. In this context, *Daphnia* spp. are proper model organisms for ecological and evolutionary studies in aquatic ecosystems as well as in laboratory conditions [8]. The density of *Daphnia* populations varies strongly within the growing season and depends on both environmental factors and biotic interactions [9]. Because density is the essential parameter determining the functional importance of *Daphnia* in an ecosystem, we focused our study on this parameter and on selected factors that could potentially significantly affect density. *Daphnia longispina* attains a relatively large body size (0.8 to 2.1 mm) in the studied ecosystem, which indicates the potential strength of the top-down effect through their grazing. Unfortunately, during summer months, this effect is unsettled due to annual toxic cyanobacterial blooms in the Sulejow Reservoir. *Microcystis aeruginosa* (Kutzing), which produces microcystin-LR, microcystin-YR and microcystin-RR, is the dominant bloom-forming cyanobacteria in this reservoir [10]. Cyanobacterial filaments and colonies are inadequate and/or inedible food for grazers due to their large size, poor nutritional value and toxicity [11]; thus, *Daphnia* spp. usually exhibit decreased survival and reproduction potential in the presence of cyanobacteria [12]. However, our previous studies have demonstrated that *D*. *longispina*, which have coexisted with a high biomass of toxic cyanobacteria in the Sulejow Reservoir, have effective protection mechanisms against the toxic effects of microcystins [13, 14]. These new facets may indicate that the *Daphnia*–cyanobacteria relationship is more complex than previously thought and that the presence of toxic cyanobacterial blooms is not always related to the collapse of a *Daphnia* population. To verify their impact on *D*. *longispina* population dynamics, we included cyanobacteria as a biotic parameter in this long-term study. We did not evaluate the grazing pressure of *D*. *longispina* on phytoplankton in our study; however, chlorophyll *a* was used as the indicator of the food base availability for daphniids because it provides a reasonable estimate of algal biomass [15, 16].

As the next biotic factor that could potentially limit the density of zooplankton species, including *D*. *longispina*, the predatory *Leptodora kindtii* was taken into account. This decision resulted from our earlier studies indicating that the role of fish in the reduction of daphniid density in the pelagic zone of the Sulejow Reservoir was much smaller than that of *L*. *kindtii*. We found that the short but intense predation of *L*. *kindtii* during the summer significantly affects the dynamics of *Daphnia* populations by causing their seasonal decline [17]. Similar predatory pressure of *L*. *kindtii* on *Diaphanosoma brachyurum* (Lievin) was observed in Lake Neusiedl [18].

In this paper, the effects of selected abiotic factors on *D*. *longispina*, *L*. *kindtii* and cyanobacteria were studied. Freshwater ecosystems undergo a dual-regulation process in which abiotic conditions (e.g., hydrology and temperature) strongly influence biota and vice versa [19]. As far as the hierarchy of these factors is concerned, the abiotic conditions play a dominant role [20], mainly by limiting the growth season of organisms (primarily due to unfavourable temperature and light conditions). The dynamics of plankton populations are closely related to the thermal gradient [21]; thus, we considered temperature as an important environmental factor that generally influences the physiology, life history and demography of planktonic organisms. The seasonal response of daphniids to temperature consists of modifications of species-specific life-history traits (such as emergence from resting stages, spawning or generation times), depending on the timing of favourable environmental conditions [22]. In this regard, one of the most critical periods for cladocerans is spring, when the temperature determines the biomass of both *Daphnia* and *L*. *kindtii*, e.g., resting eggs of *L*. *kindtii* begin to hatch to the metanauplius stage at temperatures of 8–10°C [21]. Spring temperatures also determine the timing and the strength of predators’ pressure. Wagner and Benndorf [21] found that the abundance of *L*. *kindtii* above which its predatory impact on daphniids became significant occurred at water temperature of 16°C and increased rapidly at water temperatures between 19 and 24°C. Increased temperatures in autumn as well as during mild winters may have important consequences for the overwintering strategies of daphniids [23]. The mean temperature in January-March was found to determine the future seasonal dynamics of the *Daphnia* population, which, consequently, determines the springtime occurrence of the clear-water phase and the duration of the daphniid midsummer decline [24]. Cyanobacteria have a relatively slow growth rate at moderate temperatures, which considerably increases in warmer conditions, often in excess of 25°C. Consequently, in temperate ecosystems, cyanobacterial dominance and bloom formation occur during hot summers [25]. These observations demonstrate that temperature is one of the major abiotic factors shaping the patterns of plankton population dynamics in water bodies of the temperate zone.

However, the seasonal events in cladoceran and cyanobacterial density dynamics may appear mostly in ecosystems with relatively stable hydrological conditions (e.g., in lakes with low inflow rates). In dam reservoirs, the abiotic environment is not as predictable as in lakes, and the dynamics of plankton populations appear to largely depend on the hydrological regime. Hydrological instability may decrease the importance of biotic interactions, including the predator-prey relationship between *Daphnia* and *Leptodora*, through the effects of flushing on zooplankton species during flash floods and through the reduction in zooplankton biomass peaks and/or shifts in the time of their appearance caused by a short retention time [26]. Moreover, during periods of frequent flushing, the plankton community is dominated by zooplankton organisms that reproduce fast enough to offset their removal by flushing, such as rotifers and small cladocerans, and replace *Daphnia* populations [27]. During stable hydrological conditions, biotic regulation may become dominant [20]. A long residence time and enhanced water stratification usually promote cyanobacterial growth and bloom formation [28]. Therefore, both inflow and retention time were included in our study as crucial factors that influence the plankton population dynamics in a dam reservoir.

The aforementioned abiotic and biotic factors interact with each other in a complex manner, underlying the reason behind the typical strongly non-linear relations between these factors and the population dynamics of plankton [29]. Thus, the seasonal dynamics of *D*. *longispina*, *L*. *kindtii* and cyanobacteria should be analysed with appropriate mathematical techniques. Hence, we decided to apply a Kohonen self-organising map (SOM), which is an unsupervised artificial neural network (ANN). SOMs effectively recognise patterns in complex data sets, even if 1) the relationships between variables are non-linear; the SOMs, in contrast to classical statistical methods, are applicable to data expressed at different measurement scales [30], and/or 2) the distribution of the data is non-normal. The latter problem is encountered particularly often in environmental studies because of the presence of many zeroes in data sets with an abundance of species (e.g., organism counts). Such variables, even when transformed, seldom satisfy the assumptions for normality of distribution [31]. In contrast to the classical statistical approach, analysis with SOMs requires no *a priori* specification about the underlying model. Moreover, SOMs reduce *n*-dimensional data to a two-dimensional map, which is interpreted quite easily, i.e., similar to the results of principal component analysis (PCA; similar objects are neighbouring, whereas different objects are distant) [32, 33].

The aims of this study, accomplished through use of an SOM, were as follows: i) to recognise long-term patterns (1999–2008) in the seasonal dynamics of biotic elements (cyanobacteria, *Daphnia longispina* and *Leptodora kindtii*) despite the high variation of abiotic and biotic parameters between years and ii) to determine the influence of seasonal changes in abiotic (temperature and hydrological conditions) and biotic parameters on the population dynamics of *D*. *longispina* and *L*. *kindtii* and on the biomass dynamics of cyanobacteria in the Sulejow Reservoir.

## Materials and Methods

### Ethics statement

No specific permits were required for the field studies described herein. There was no activity involving endangered or protected species in this study.

### Study area

**Fig 1:**
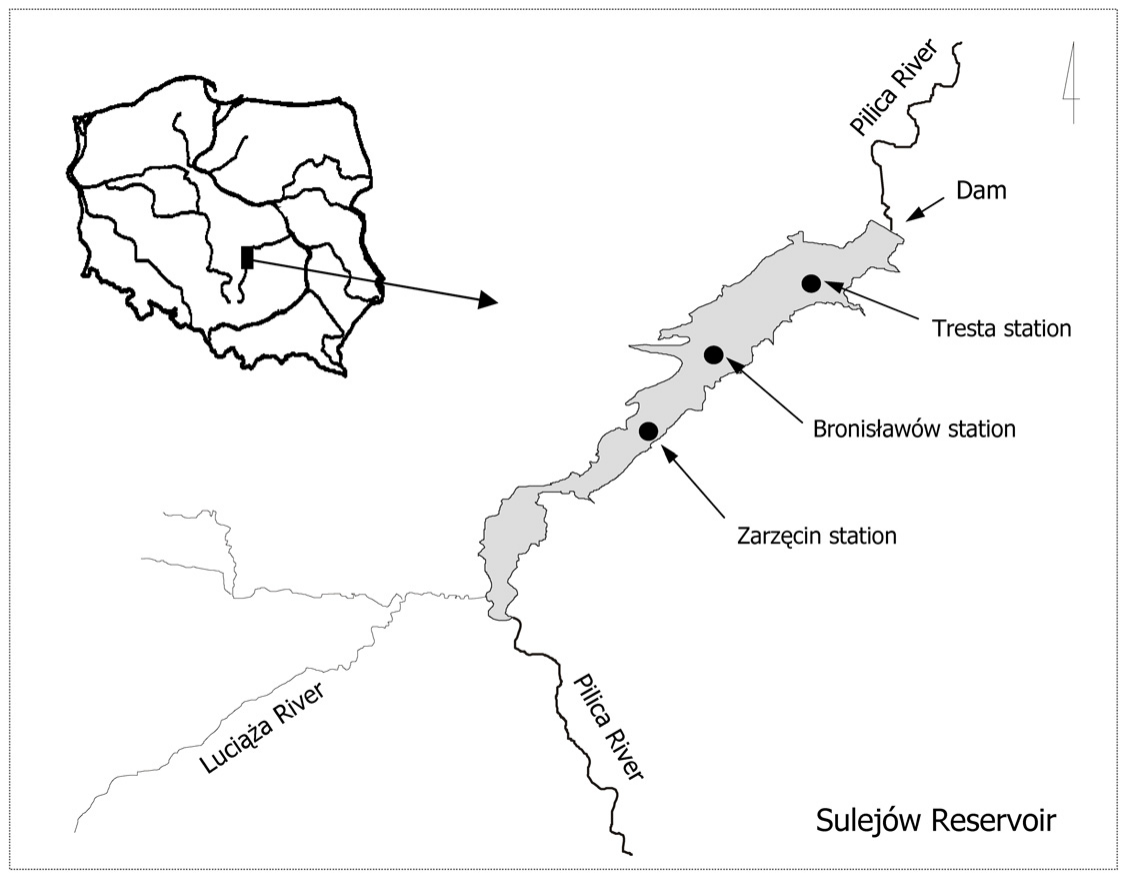
The study area. Map of the Sulejow Reservoir showing the distribution of the sampling stations.

The Sulejow Reservoir is a shallow reservoir that comprises 138.9 km of the Pilica River (the Vistula River catchment) in central Poland (Fig 1). The reservoir was built in 1973. Its maximum length is 15.5 km, and its maximum width is 2.1 km. At maximum capacity (75 x 10^6^ m^3^), the reservoir covers 1980 ha, with a mean depth of 3.3 m and a maximum depth of 11 m. The reservoir sub-catchment area is covered mainly with agricultural land (50% arable land, 13% meadows and pastures, 1% orchards) and forests (31%). The shoreline length is approximately 54 km. The Sulejow Reservoir is a non-stratified, eutrophic ecosystem [2]. In the initial stage of seasonal succession, the phytoplankton community consists mostly of diatoms, cryptophytes and green algae. The phytoplankton community in the reservoir has a typical composition for Central Europe, characterised by the dominance of the microcystin-producing genus of *Microcystis* [34]. In the summer months, blooms of mainly *Microcystis aeruginosa* Kütz. and *Aphanizomenon flos-aquae* (L.) Ralfs have been frequently observed [35].

The dominant species of cladocerans in the Sulejow Reservoir are as follows: *Daphnia longispina* (O.F. Müller), *D*. *cucullata* (Sars.), *Bosmina coregoni* and *Leptodora kindtii* (Focke) [36].

Gillnet catches have been regularly conducted since 1993 and revealed a very stable composition of the fish stock during the 1990s, with dominance (in terms of biomass) of common bream, *Abramis brama* L.; pikeperch, *Stizostedion lucioperca* L.; white bream, *Abramis bjöerkna* L.; roach, *Rutilus rutilus* L. and perch, *Perca fluviatilis* L. [37].

Studies were conducted at each of the three sampling stations located along the longitudinal reservoir axis. The sampling stations (Fig 1) included the following: an upper (Tresta = TR), a middle (Bronisławów = BR) and a lower (Zarzęcin = ZA) station. The studies were conducted every week between April and October during the years 1999–2008 for a total of 412 sampling terms. The database is presented in S1 Table.

### Plankton sampling and analyses

A total of 1236 samples (3 sampling stations × 412 sampling terms) of zooplankton and phytoplankton were collected. The samples were collected weekly in the pelagic zone of the reservoir at the three sampling stations. Water samples were taken using a 5-L sampler from each metre of the entire water column and integrated. Then, a 1-litre subsample of the integrated water was collected for the determination of cyanobacterial presence and biomass. These water samples were preserved in Lugol’s solution and sedimented in the laboratory. Algae were analysed using a Fusch-Rosenthal counting cell (procedure according [38]).

*Daphnia longispina* and *Leptodora kindtii* were sampled using a 5-L Bernatowicz sampler at four depths: one, two, three and four metres (maximal depth at the sampling stations did not exceed four metres). Samples were taken with four or five replicates for each depth to properly evaluate the density of *L*. *kindtii* [39]. Because diurnal vertical migration of zooplankton has not been observed in this shallow reservoir and zooplankton were almost evenly distributed throughout the entire water column ([36] and unpublished data based on the annual monitoring of the reservoir), samples from all four depths were integrated. Samples were then filtered with a 50 μm mesh size plankton net. Collected zooplankton were preserved in 4% Lugol’s solution. The samples were identified and counted under a Nikon 115 microscope using a Sedgewick Rafter counting chamber. Morphological analyses of collected *Daphnia* species were performed according to Benzie [40].

### Chlorophyll *a* analysis

Water samples were taken weekly using a 5-L sampler from each metre of the entire water column at the three sampling stations in the pelagic zone of the reservoir. Samples from different depths were integrated, and a 1-litre subsample was then obtained for the analysis of chlorophyll *a*. Chlorophyll *a* concentrations were analysed using a spectrophotometric method [41].

### Hydrological parameters

The water retention time of the reservoir was calculated as the ratio of the reservoir volume to the volume of water inflow to the reservoir. These data were available from the Smardzewice branch of the Regional Water Management in Warsaw (http://warszawa.rzgw.gov.pl/the-regional-water-management-authority-in-warsaw), which administers the Sulejow Reservoir and conducts permanent statutory monitoring of the hydrological parameters according to Polish and EU standards.

The hydrological data relate to the same dates as the collection of other parameters. The same values of retention time and water inflow to the reservoir were used for hydrological characteristics of the sampling station. We did not consider the pattern of water inflow propagation along the reservoir and assumed that, due to the specific reservoir shape, the biota were impacted by inflowing water in a similar way at all three sampling stations, especially during flood events. The coefficient of water inflow variation (CV) was used to classify years as hydrologically stable or instable and was calculated as the ratio of the standard deviation and the mean of the water inflows to the reservoir in June-September, expressed as a percentage.

### Temperature

Water temperature (°C) was measured between 10:00 and 11:00 a.m. at each sampling term in the offshore area of the Sulejow Reservoir at 1.5 m depth using WTW 340i/SET multisensors.

### SOM analysis

The patterns in the biotic components (nine variables: the biomass of cyanobacteria and the densities of *D*. *longispina* and *L*. *kindtii* at the three stations) were recognised using Kohonen’s artificial neural network, i.e., a self-organizing map (SOM) [32]. An SOM is constructed from two (input and output) layers of data processing units (neurons). The SOM training was performed using the SOM Toolbox available at http://www.cis.hut.fi/projects/somtoolbox/ [42], developed in the Laboratory of Information and Computer Science at the Helsinki University of Technology. During the network training (using the sequential algorithm), the log-transformed data (nine variables × 412 sampling terms; ST) were repeatedly displayed on nine input neurons (their number = the number of variables). The input neurons, each connected to all of the output neurons, transmitted information to the output layer of the neurons. On this basis, a virtual ST (understood as a set of values of the nine analysed variables) was created in each output neuron. The virtual STs (and thus the respective output neurons) were clustered with hierarchical cluster analysis (Ward linkage method, Euclidean distance), which is a standard procedure in the SOM Toolbox [43]. The relative position of any two virtual STs on the SOM reflects their similarity; virtual STs in neighbouring neurons are similar, whereas those in distant neurons are different. Finally, each real ST was assigned to the best matching virtual ST (and the respective output neuron and cluster of output neurons); similar real STs were located in the same neuron or in adjoining neurons and were considerably different from the real STs located in distant neurons.

We decided to input STs (with values for the three stations treated as separate variables) to the SOM instead of individual samples. The latter solution was more intuitive but would cause problems in further analyses mostly because of the unbalanced statistical influence of a given ST on the values calculated for output neurons containing different numbers of dependent samples from that ST. The adopted solution allowed us to assign STs (all of equal merit) univocally to output neurons (and their (sub-)cluster) and to analyse different effects, including the differences between sampling stations, with more conventional analyses.

We finally adopted the classification of the SOM output neurons into three clusters and six sub-clusters, resulting in a fairly symmetrical division of the SOM, i.e., the particular clusters or sub-clusters of virtual units corresponded to similar parts of the total mapped variability.

The SOM Toolbox allowed visualisation of the intensity of particular variables in output neurons in the form of a greyness gradient over the SOM [33]. It was possible for both density and biomass (biomass of cyanobacteria, densities of *D*. *longispina* and *L*. *kindtii*, all input to the SOM) to explain the remaining variables (chlorophyll *a*, water temperature, water inflow to the Sulejow Reservoir and the retention time of water in the reservoir, not presented directly to the SOM). The greyness intensity reflected 1) the values of density/biomass variables in virtual STs (i.e., in respective output neurons) and 2) the mean values of the remaining variables calculated for real STs assigned to a given output neuron.

The number of output neurons (24 on a two-dimensional, 6 × 4 grid) was arbitrarily selected from the other tested options (from 4 × 4 to 12 × 9). Although there was no universal principle to determine the optimum map size, we considered quality measures for SOMs such as the quantisation (QE) and topographic (TE) errors [43]. The former is the average distance between each real ST and its best matching virtual ST, whereas the latter is the proportion of all real STs for which the first and second best-matching virtual STs were not in adjacent neurons [44]. In our study, similar QE and TE were observed for larger maps compared to the 6 × 4 map that was ultimately adopted (S2 Table). Some of the larger maps corresponded to the often-applied rule stating that the number of output neurons should be close to 5√n, where n is the number of real STs [45]. Our decision on the actual number of output neurons in the map was influenced by 1) obtaining clear patterns and 2) avoiding output neurons that were either empty (i.e., without any real STs) [43] or contained 1–2 real STs assigned, such that the aforementioned greyness used to visualize the values of variables in such neurons did not assume a random intensity, which would disturb the general greyness gradients.

### Canonical correlation analyses

We used canonical correlation analyses (CCA) to explore the relationship between abiotic and biotic sets of variables that were measured in the reservoir during the entire investigation period. Inflow, retention time, temperature and chlorophyll *a* were chosen as predictor variables, and cyanobacterial biomass, *D*. *longispina* density, and *L*. *kindtii* density were used as response variables. Data from each sampling term represented the average values of the three sampling stations. Prior to the analyses, all data were log (x+1) transformed for variance stabilisation. CCA were performed separately for the three main clusters of SOM output neurons. By analysing data subsets characterised by relatively reduced variability, we expected to reveal more subtle effects that were presumably different for particular SOM clusters.

### Statistical tests

We decided to explore the data at a lower typology level by comparing the biotic and abiotic variables between 1) the six sub-clusters of SOM output neurons and 2) the three sampling stations within each SOM sub-cluster. Because the SOM does not provide a statistical verification of the observed differences, the significance of differences between the SOM sub-clusters was assessed by the Kruskal-Wallis and *post-hoc* Dunn tests [46] in the Statistica package (Statistica 12, StatSoft, Inc.). The significance of differences between the three sampling stations was assessed with the Friedman test for multiple groups of dependent samples [46] in the XLSTAT package. The Kruskal-Wallis and Friedman tests are non-parametric (based on ranks) equivalents of the analysis of variance that allow for testing of whether more than two groups of samples differ. The groups compared with the Kruskal-Wallis test may contain different numbers of samples, whereas groups compared with the Friedman test must be of the same size. Similar to the SOM, both tests can be applied to data of a normal or skewed distribution. Because these tests indicate the existence of a significant difference but do not indicate how many groups of samples and which of them differ, a *post-hoc* test should be subsequently applied for multiple comparisons between the groups [46].

## Results

### The dynamics of abiotic and biotic parameters over ten years (1999–2008) of study

**Fig 2:**
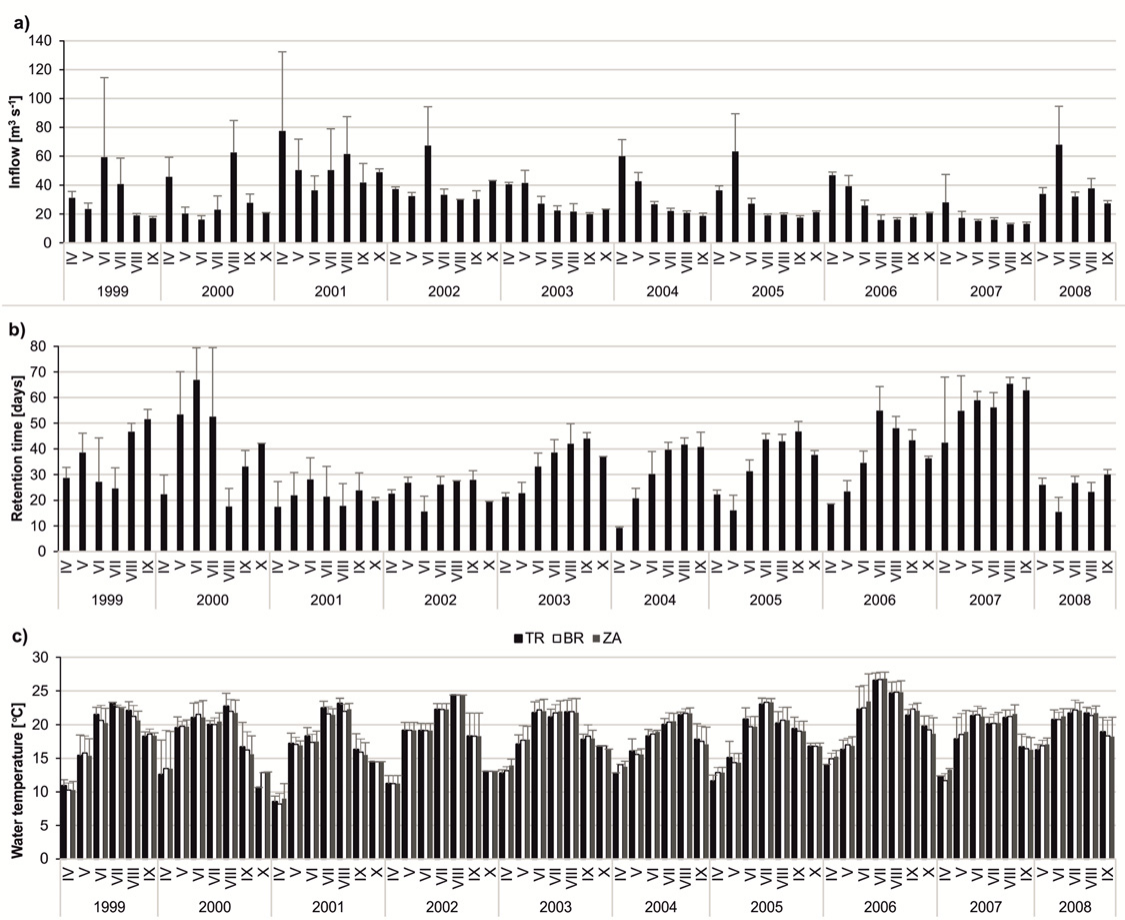
Dynamics of the selected abiotic parameters over the study period of ten years (1999–2008) at three stations. (A) inflow (m^3^ s^-1^); (B) retention time (days); (C) temperature (°C). The bars show the monthly average with standard deviations.

In all the studied years, a high water stage in the spring months (April–May) was observed. However, later on, the hydrological regime varied within the ten-year period (Fig 2A and 2B). To classify the years as hydrologically stable or unstable, the coefficient of water inflow variation in June-September was considered. The years 1999, 2000, 2001, 2002 and 2008, which were characterised by many inflow peaks and short retention times, had the highest values of CV (ranging between 46–94%) and were regarded as hydrologically unstable. In the years 2003, 2004, 2005, 2006 and 2007, the coefficient of water inflow variation was substantially lower (ranging between 12–26%), and these years were classified as hydrologically stable. The most hydrologically stable year was 2007, whereas the most unstable year (high inflow in both spring and summer) was 2001. The year 2001 was also the coldest, with a median seasonal (from April to October) water temperature of 17.57°C in ZA, 17.52°C in BR and 18.16°C in TR. The warmest year was 2006, with a median seasonal water temperature of 22.21°C in ZA, 22.14°C in BR and 21.71°C in TR (Fig 2C). A warm summer characterised years 2005–2008, whereas a particularly warm spring was observed in 2006 and 2008.

**Fig 3:**
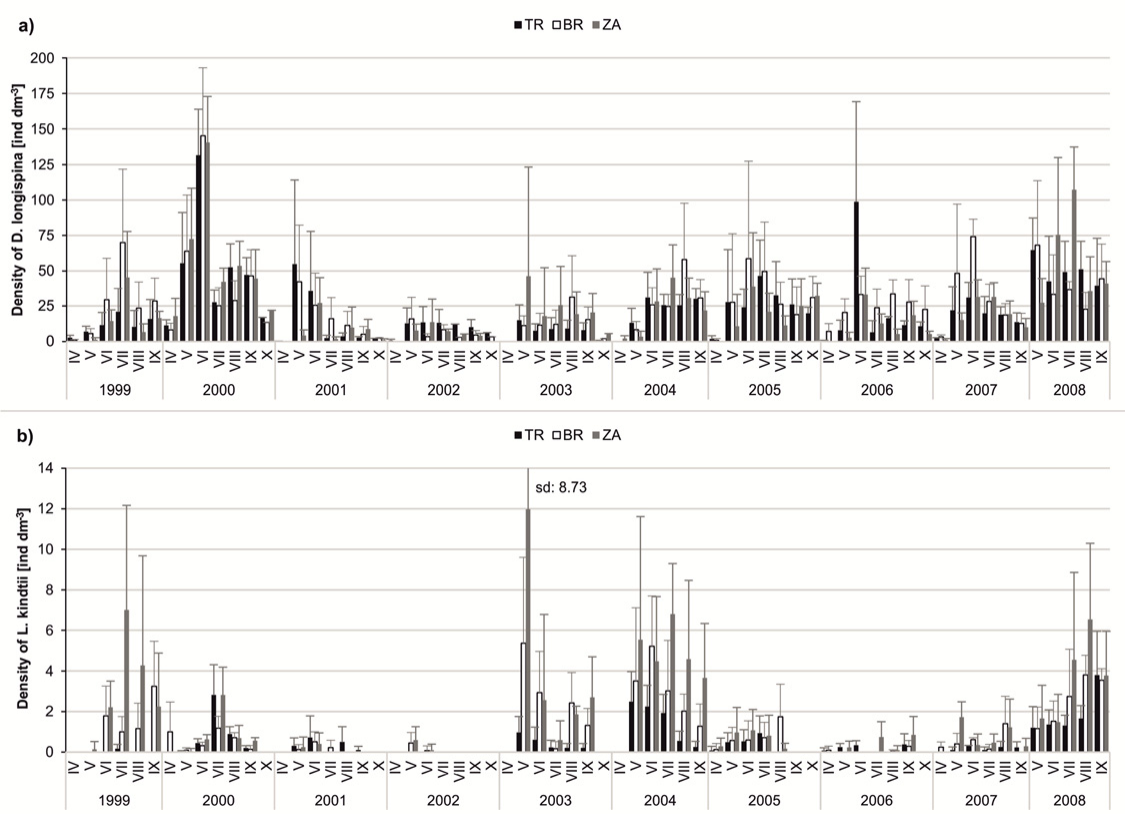
Dynamics of the selected biotic parameters over the study period of ten years (1999–2008) at three stations. (A) dynamics of *Daphnia longispina* density (ind dm^-3^); (B) dynamics of *Leptodora kindtii* density (ind dm^-3^). The bars show the monthly average with standard deviations.

**Fig 4:**
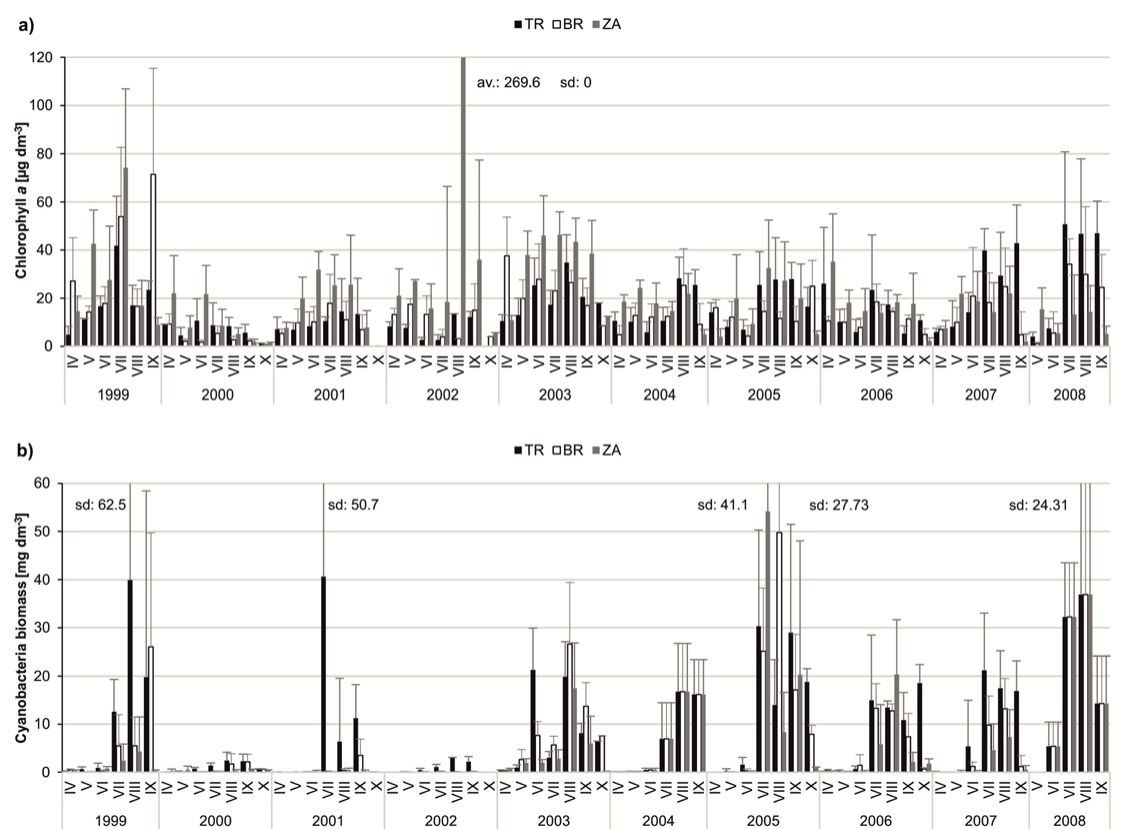
Dynamics of the selected biotic parameters over the study period of ten years (1999–2008) at three stations. (A) dynamics of chlorophyll *a* concentration (μg dm^-3^); (B) dynamics of cyanobacterial biomass (mg dm^-3^). The bars show the monthly average with standard deviations.

We observed a high variation in both abiotic and biotic parameters among years, which disturbed the long-term patterns in the population dynamics of *Daphnia longispina*, *Leptodora kindtii* and cyanobacteria. The density of *D*. *longispina* was very diverse in 1999–2008. The lowest daphniid density was observed in the early spring in all years, but their maximal peaks appeared at different times of each year. The year 2000 was characterised by the highest density of *D*. *longispina*; in June, their median density amounted to 140.59 ind dm^-3^ in ZA, 145.33 ind dm^-3^ in BR and 131.68 ind dm^-3^ in TR. In turn, the lowest density of daphniids was observed in 2002, with the median monthly value not exceeding 15.92 ind dm^-3^ (Fig 3A). A high abundance of *L*. *kindtii* was observed in 1999, 2003, 2004 and 2008, particularly in ZA where the median seasonal density was 7 ind dm^-3^ (in 1999), 11.98 ind dm^-3^ (in 2003), 6.80 ind dm^-3^ (in 2004) and 6.54 ind dm^-3^ (in 2008). The TR station was characterised by the lowest density of *L*. *kindtii* among the three stations in all studied years (Fig 3B). The values of chlorophyll *a* were diverse over the 10-year period, but the dynamics of its concentration followed a similar pattern in all years; high values usually persisted from May to September (Fig 4A). Cyanobacterial blooms developed mainly from July to September and were the most intense at the TR and BR stations. In 2000, blooms were not observed (Fig 4B).

### Ordination of sampling terms with the self-organising map

**Fig 5:**
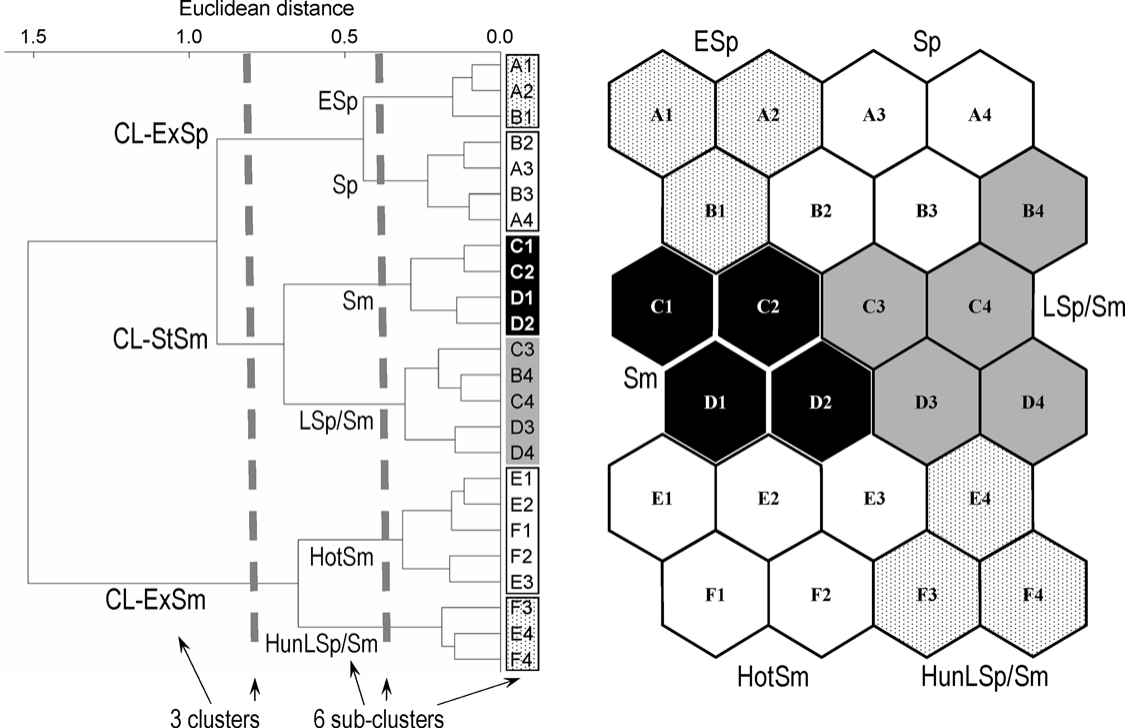
The 24 SOM output neurons arranged into a two-dimensional grid (6 × 4). Neurons were grouped into three clusters (CL-ExSp, CL-StSm and CL-ExSm; separated by dashed lines) and six sub-clusters (ESp, Sp, LSp/Sm, Sm, HotSm, HunLSp/Sm; shown using different degrees of greyness) through hierarchical cluster analysis.

In the output layer of the SOM, the following three main clusters of neurons were distinguished (Fig 5):

1. CL-ExSp (abbr. extreme/spring; including neurons A1-B3), referring to an “unstable cold season” characterized by extreme values and a high variability of abiotic factors, resulting in a dominance of the abiotic control over the biota.
2. CL-StSm (abbr. stable/summer; including neurons B4-D4), characterised by stable non-extreme abiotic conditions, making biotic interactions more important.
3. CL-ExSm (abbr. extreme/summer; including neurons E1-F4), corresponding to a “warm season with thermal or hydrological extremes” when the role of biotic control was decreased due to extreme values and/or a high variability of abiotic factors.

Each main cluster was composed of two sub-clusters: ESp (“early spring”: neurons A1, A2, B1) and Sp (“spring”: neurons A3, A4, B2, B3) in CL-ExSp, LSp/Sm (“late spring/summer”: neurons B4, C3, C4, D3, D4) and Sm (“summer”: neurons C1, C2, D1, D2) in CL-StSm, and HotSm (“hot summer”: neurons E1-E3, F1, F2) and HunLSp/Sm (“hydrologically unstable late spring/summer”: neurons E4, F3, F4) in CL-ExSm (Fig 5).

In the above, we intentionally used alternative terms for spring and summer because of the differences between years, which were large enough to make usage of the words “spring” and “summer” and analyses based on such divisions misleading. This is best shown by the uneven distribution of sampling terms from some years over the SOM. For example, the coldest and most hydrologically unstable year, 2001, was almost entirely a spring in the ecological sense (most of the sampling terms from 2001 are assigned to ESp (“early spring”), Sp (“spring”) and LSp/Sm (“late spring/summer)). The year 2000 was very homogenous because the majority of its sampling terms were grouped in LSp/Sm (“late spring/summer”). In contrast, the year 2008 was almost completely devoid of the ecological spring phase because few sampling terms were assigned to ESp (“early spring”), Sp (“spring”) and LSp/Sm (“late spring/summer”). In the summer of that year, similar to 2004, the most extreme conditions were recorded (sampling terms from 2004 and 2008 are the most frequent in cluster CL-ExSm “Extreme/Sumer”) (S1 Fig). In this paper, we distinguished ecological states or ecological seasons (because the SOM classification was based only on density/biomass data for plankton components) and then looked for explanations of the observed patterns. The above differences between years show the advantage of our approach over others based on the calendar and/or classical seasons.

**Fig 6:**
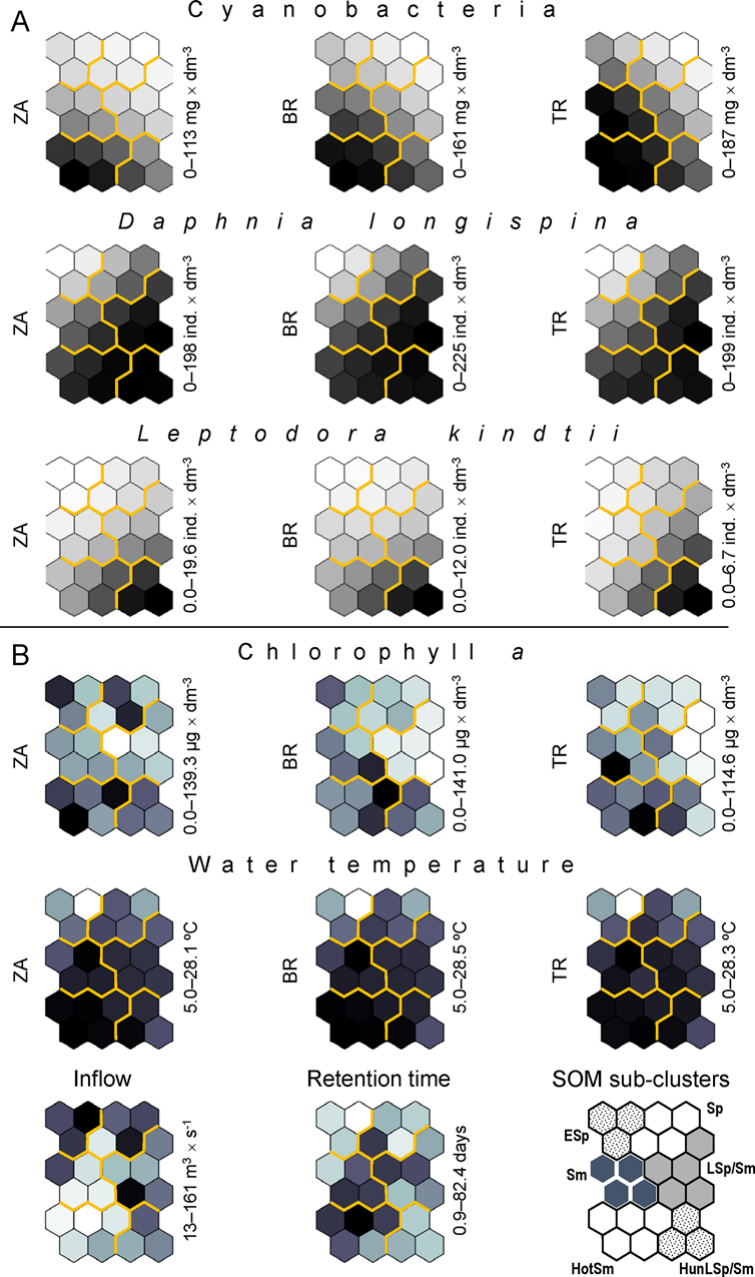
The intensity (stronger = darker shading) of nine density/biomass variables (A; used for SOM training) and eight explanatory variables (B; not used for SOM training) in particular SOM regions. Values of the A variables come from the virtual sampling terms (one per output neuron), whereas values of the B variables are the means of the real sampling terms assigned to a given output neuron (see S1 Fig). The intensity of the grey colour is scaled independently for each variable (the range of variability in the data set is presented on the right side of each plane). Zarzęcin (ZA), Bronisławów (BR) and Tresta (TR) are the sampling stations. See Figs 7 and 8 for statistical verification of the differences between the SOM sub-clusters.

**Fig 7:**
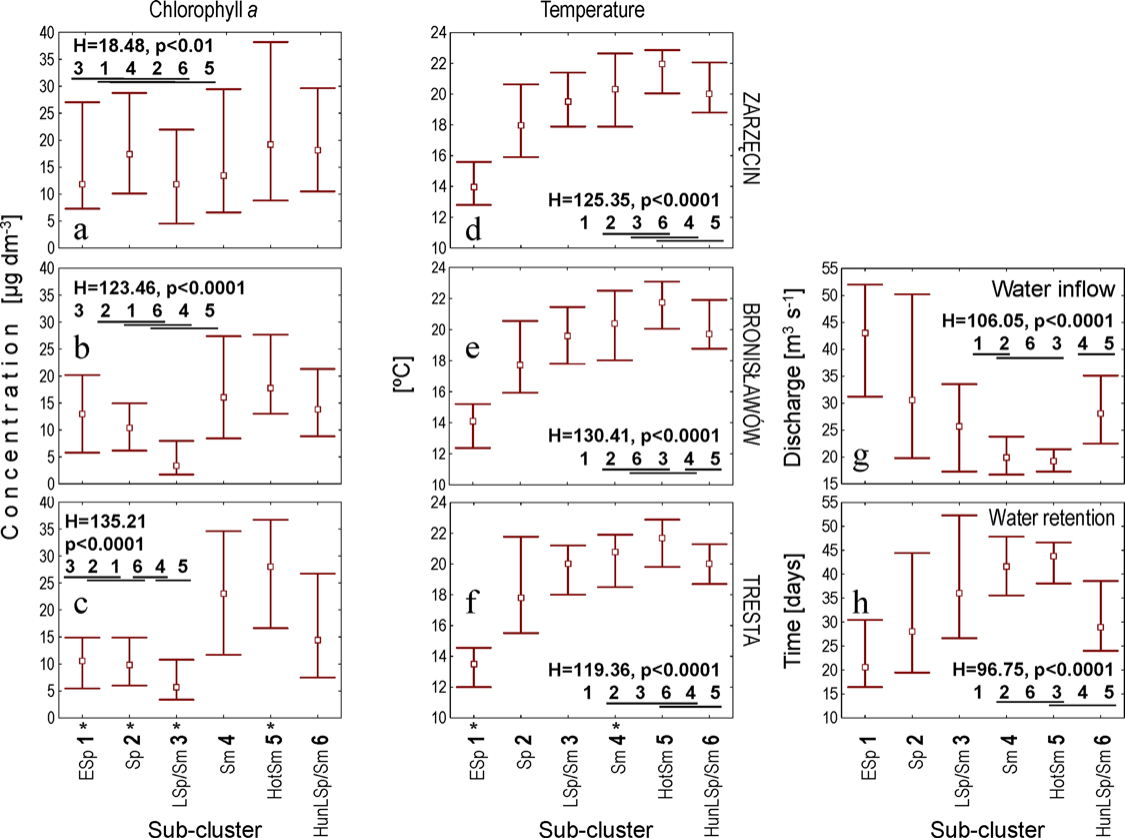
Explanatory variables that were not presented directly to the Kohonen artificial neural network. Chlorophyll *a* (a-c), water temperature (d-f) at the Zarzęcin (a, d), Bronisławów (b, e) and Tresta (c, f) stations; water inflow to the Sulejow Reservoir (g) and time of water retention in the reservoir (h). Point–median; whiskers–interquartile range; H–statistics of the Kruskal-Wallis test (df = 5, N_ESp_ = 66, N_Sp_ = 46, N_LSp/Sm_ = 106, N_Sm_ = 57, N_HotSm_ = 80, N_HunLSp/Sm_ = 57) applied in testing the differences between the SOM sub-clusters (the latter is underlined with the same line if not different at p ≤ 0.05 in *post-hoc* tests). An asterisk indicates SOM sub-clusters for which a significant difference between sampling stations was recorded (see Table 1 for details). Abbreviations of the sub-clusters names: **1** ESp–early spring; **2** Sp–spring; **3** Sp/Sm–spring/summer; **4** Sm–summer; **5** HotSm–hot summer; **6** HunLSp/Sm—hydrologically unstable late spring/summer.

The order of sub-clusters LSp/Sm and Sm was determined on the basis of the gradients observed in abiotic factors (Figs 6 and 7). The water temperature at the three sampling stations increased for the successive five sub-clusters, from ESp to HotSm (ESp, Sp, LSp/Sm, Sm and HotSm), and the temperature of HunLSp/Sm was lower than that of HotSm. The temperature of HunLSp/Sm was similar to that of LSp/Sm or Sm. A similar upward trend was recorded for five sub-clusters, from ESp to HotSm, in the retention time of water in the reservoir; the median for HunLSp/Sm was similar to that for Sp. The inflow of water to the reservoir decreased in five successive sub-clusters ESp–HotSm and increased in HunLSp/Sm (Fig 7).

**Fig 8:**
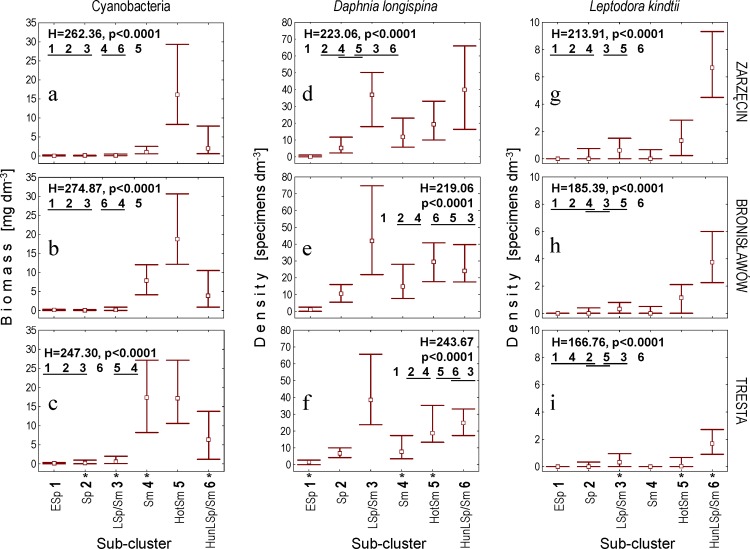
Density/biomass variables presented directly to the Kohonen artificial neural network. Biomass of cyanobacteria (a-c), densities of *Daphnia longispina* (d-f) and *Leptodora kindtii* (g-i) at the Zarzęcin (a, d, g), Bronisławów (b, e, h) and Tresta (c, f, i) stations. Explanations are the same as in Fig 7.

Regarding the biotic variables, the patterns that best reflected those mentioned above (for the abiotic factors) were recorded for cyanobacterial biomass, which increased in five successive sub-clusters ESp–HotSm and decreased in HunLSp/Sm (Figs 6 and 8). Increases in cyanobacterial biomass were also visible for four sub-clusters when moving downstream (from ZA to TR); the most distinct of these increases were recorded for Sm, LSp/Sm and HunLSp/Sm. Chlorophyll *a* increased in three successive sub-clusters, LSp/Sm, Sm and HotSm, and the differences were more pronounced downstream (Fig 8, Table 1).

**Table 1:** Spatial differences (between the three sampling stations) in biotic variables and water temperature.

Variable and SOM sub-cluster	ZA	BR	TR	N	Q observed	Significance level
Biomass of cyanobacteria						
ESp				66	2.520	ns
Sp	a	a	b	46	12.198	p < 0.01
LSp/Sm	a	a	b	106	31.849	p < 0.0001
Sm	a	b	c	57	73.286	p < 0.0001
HotSm				80	3.370	ns
HunLSp/Sm	a	a	a	57	8.111	p < 0.05
Density of *D*. *longispina*						
ESp	a	b	b	66	21.474	p < 0.0001
Sp				46	3.945	ns
LSp/Sm				106	4.402	ns
Sm	a, b	a	b	57	16.062	p < 0.001
HotSm	a	b	a	80	11.775	p < 0.01
HunLSp/Sm				57	5.612	ns
Density of *L*. *kindtii*						
ESp				66	4.512	ns
Sp				46	2.275	ns
LSp/Sm	a	b	a, b	106	11.362	p < 0.01
Sm				57	5.460	ns
HotSm	a	a	b	80	50.336	p < 0.0001
HunLSp/Sm	a	b	c	57	48.329	p < 0.0001
Concentration of Chl-*a*						
ESp	a	a	b	66	18.350	p < 0.0001
Sp	a	b	b	46	33.391	p < 0.0001
LSp/Sm	a	b	c	106	48.931	p < 0.0001
Sm				57	5.612	ns
HotSm	a, b	a	b	80	7.825	p < 0.05
HunLSp/Sm				57	5.982	ns
Water temperature						
ESp	a	a	a	66	6.177	p < 0.05
Sp				46	0.893	ns
LSp/Sm				106	4.805	ns
Sm	a	a, b	b	57	10.430	p < 0.01
HotSm				80	0.423	ns
HunLSp/Sm				57	0.699	ns

Sampling stations: ZA–Zarzęcin, BR–Bronisławów, TR–Tresta. N–number of samples, Q–statistics of the Friedman test (df = 2), Q critical is 5.991. For sampling stations marked with the same symbol in a given SOM sub-cluster (row) no significant difference (*p*≤ 0.05) in *post-hoc* comparisons was recorded.

Another pattern was observed in the densities of the two Cladocera species. The densities of *Daphnia longispina* and *Leptodora kindtii* generally increased in sub-clusters ESp, Sp and LSp/Sm, followed by (after they dropped in Sm compared to LSp/Sm) HotSm and HunLSp/Sm (Figs 6 and 8). Additionally, *L*. *kindtii* decreased downstream in HotSm and HunLSp/Sm (Fig 8, Table 1).

Detailed results of the Kruskal-Wallis and *post-hoc* Dunn tests used for statistical verification of the significance of observed differences between the SOM sub-clusters are presented in Figs 7 and 8, and results of the Friedman test applied to assess the significance of differences between the three sampling stations are presented in Table 1.

### Characteristics of abiotic and biotic factors in sampling terms assigned to the SOM sub-clusters

All sampling terms were assigned to the neurons in the CL-ExSp, CL-StSm and CL-ExSm clusters according to their biotic and abiotic characteristics (more details in Table 2):

1. Sub-cluster ESp (“early spring”) grouped sampling terms with very low densities of *D*. *longispina* (median of 1.19 ind dm^-3^) and *L*. *kindtii* (median close to zero). The median concentration of chlorophyll *a* amounted to 11.84 μg dm^-3^, but the median cyanobacterial biomass was 0.09 mg dm^-3^. Sampling terms grouped in ESp were characterised by high values of inflow (median > 40 m^3^ s^-1^), very short retention times (median < 21 days) and low temperatures (13.86°C at three stations). Such unstable hydrology, low temperatures and low values of biotic parameters were specific to the period of early spring. Sampling terms grouped in ESp did come predominantly from May and April.
2. Sub-cluster Sp (“spring”) grouped terms in which the median density of *D*. *longispina* increased to 5.79 ind dm^-3^ but the density of *L*. *kindtii* remained close to zero. Values for the chlorophyll *a* concentration and cyanobacterial biomass were similar to those in sub-cluster ESp and reached medians of 12.38 μg dm^-3^ and 0.14 mg dm^-3^, respectively. Sp included the sampling terms of May and June with persistent unstable hydrology (median inflow value > 30 m^3^s^-1^ and retention time < 29 days). The median temperature was 17.98°C.
3. Sub-cluster LSp/Sm (“late spring/summer”) grouped terms with a high density of *D*. *longispina* (median of 39.15 ind dm^-3^) and relatively low concentrations of chlorophyll *a* (median of 6.95 μg dm^-3^), which was characteristic for the clear water phase. The cyanobacterial biomass was still very low (median of 0.30 mg dm^-3^), as was the density of *L*. *kindtii* (median of 0.42 mg dm^-3^). It reflected late spring conditions characterised by more stable hydrological parameters (median inflow value < 26 m^3^ s^-1^ and retention time > 35 days) and higher temperatures (median 19.83°C). The sampling terms of this sub-cluster corresponded mostly to June, as well as May.
4. In terms grouped in sub-cluster Sm (“summer”), the median density of *D*. *longispina* amounted to 11.45 ind dm^-3^. The density of *L*. *kindtii* was still very low (0.33 ind dm^-3^). A high cyanobacterial biomass was observed only at the TR station (median of 17.33 mg dm^-3^), whereas it reached a median of 7.89 in BR and only 1.04 mg dm^-3^ in ZA. The terms of Sm were also characterised by a high chlorophyll *a* concentration (median of 17.52 μg dm^-3^) and a relatively high temperature (median of 20.54°C), as well as stable hydrological parameters: a low inflow (< 20 m^3^s^-1^) and a long retention time (> 40 days). This sub-cluster contained the sampling terms of July and September.
5. Sub-cluster HotSm (“hot summer”) contained the terms of August, July and September. The median density of *D*. *longispina* was 22.49, while that of *L*. *kindtii* was 0.84 ind dm^-3^. In sampling terms assigned to HotSm, high cyanobacterial biomass was observed at all three stations (median of 17.35 mg dm^-3^). The median concentration of chlorophyll *a* amounted to 21.64 μg dm^-3^. Sampling terms grouped in HotSm were characterised also by very low inflow values (< 19.5 m^3^s^-1^) and long retention times (> 43 days). The median temperature reached 21.78°C.
6. Sub-cluster HunLSp/Sm (“hydrologically unstable late spring/summer”) grouped both spring (May, June) and summer (July, August, September) sampling terms. The sampling terms of HunLSp/Sm were characterised by a relatively high density of *D*. *longispina* (median of 29.61 ind dm^-3^) and the highest density of *L*. *kindtii* of all sub-clusters (median of 4.04 ind dm^-3^). The chlorophyll *a* concentration reached a median of 15.43 μg dm^-3^, but the cyanobacterial biomass amounted to only 4.06 mg dm^-3^. The hydrological parameters were rather unstable: the median inflow reached > 28 m^3^ s^-1^, and the retention time was > 28.5 days. The median temperature was 19.85°C.

**Table 2:** Characteristics of abiotic and biotic factors in the sampling terms assigned to the SOM sub-clusters.

	SOM sub-clusters					
	ESp	Sp	LSp/Sm	Sm	HotSm	HunLSp/Sm
**1. Dominant months of samples collection assigned to the sub-cluster**						
	April (49%)	May (35%)	June (39%)	September (35%)	August (50%)	July (30%)
	May (25%)	June (22%)	May (28%)	July (30%)	July (27%)	June (21%)
					September (22%)	May (18%)
						September (18%)
						August (14%)
**2. Dominant season of the year**						
	Early spring	Spring	Late spring / summer	Summer	Hot summer	Hydrologically unstable late spring/summer
**3. Characteristics of selected parameters:**						
a) Water temperature (°C)	7.14–17.83	12.91–23.83	15.90–24.40	14.69–28.54	16.88–28.10	16.30–23.40
Medians:	< 14.2	18.19–18.80	19.72–20.00	20.30–20.77	> 21.5	19.70–20.00
b) Inflow (m^3^ s^-1^)	20.85–158.30	19.40–78.90	12.60–83.00	14.70–51.10	12.61–38.84	18.30–54.91
Medians:	> 40	> 30	< 26	< 20	< 19.5	> 28
c) Retention time (days)	6.50–41.08	11.2–71.86	11.00–65.44	17.60–60.75	24.10–68.95	16.50–48.10
Medians:	< 21	< 29	> 35	> 40	> 43	> 28.5
d) Chlorophyll *a* concentration (μg dm^-3^)	2.10–74.33	3.04–68.42	0.77–34.44	5.01–67.60	8.97–114.06	1.73–139.33
Medians:	11.52–13.24	9.55–17.35	3.40–11.77	13.48–23.07	17.73–28.03	13.78–18.10
e) Cyanobacteria biomass (mg dm^-3^)	0.01–3.35	0.01–2.11	0.01–5.41	1.75–95.23	6.13–160.56	0.03–98.36
Medians:	< 0.13	< 0.24	< 0.60	1.04–17.33	16.11–18.76	1.97–6.31
f) *D*. *longispina* density (ind dm^-3^)	0.20–8.67	1.15–53.75	13.72–224.80	2.00–49.00	1.67–158.00	2.33–198.00
Medians:	< 1.4	5.22–10.51	36.84–41.89	7.68–14.67	18.75–29.35	24.00–40.00
g) *L*. *kindtii* density (ind dm^-3^)	0.25–4.00	0.15–4.67	0.25–5.26	0.14–2.98	0.33–16.00	0.10–19.57
Medians:	0.00	0.00	0.33–0.61	0.00	0.03–1.33	1.69–6.67

### Results of canonical correlation analyses

For cluster CL-ExSp (extreme/spring), only the first canonical correlation (0.678; p<0.01), which was calculated between the first and second correlation pairs, was significant (Table 3). The highest contribution to the formation of the U1 canonical root for predictor variables belonged to retention time, with a canonical weight of 1.252. The highest contribution to the formation of the V1 canonical root for the response variables was the *D*. *longispina* density (0.845). Considering the values of the factor loadings, which showed the overall correlation of the respective variables with the canonical roots, the highest loading for the U1 and V1 canonical roots was temperature (0.890) and *D*. *longispina* density (0.897), respectively. The redundancy of response variables calculated for the first canonical correlation indicated that 17.5% of the biotic factor variance was explained by predictor variables. When the correlation coefficients between abiotic and biotic factors were examined (Table 3), most of them were statistically significant; the correlation coefficients between biotic factors were very low, and all but one was statistically insignificant.

**Table 3:** Results of the canonical correlation analyses for cluster CL-ExSp of the SOM output neurons.

Canonical roots for predictor variables		U1	U2	U3	U1	U2	U3
		Canonical weights			Canonical factor loadings		
*Infl*		-0.851	-1.807	0.702	0.496	-0.493	-0.684
*Rete*		-1.252	-1.479	1.573	-0.538	0.411	0.734
*Temp*		-0.860	-0.211	-0.523	-0.890	0.053	-0.447
*Chla*		0.160	0.826	-0.310	-0.107	0.881	-0.296
Canonical roots for response variables		**V1**	V2	V3	V1	V2	V3
		Canonical weights			Canonical factor loadings		
*Cyan*		-0.441	0.731	-0.527	-0.507	0.679	-0.530
*Daph*		-0.845	-0.605	0.033	-0.897	-0.388	0.222
*Lept*		-0.064	0.606	0.839	-0.283	0.442	0.851
Canonical correlations		**0.678**	0.143	0.055			
Redundancy of response variables		17.49%	0.60%	0.10%			
	Correlations between variables						
	*Infl*	*Rete*	*Temp*	*Chla*	*Cyan*	*Daph*	*Lept*
*Infl*	1.000						
*Rete*	**-0.982**	1.000					
*Temp*	-0.177	0.177	1.000				
*Chla*	**-0.212**	**0.186**	**0.249**	1.000			
*Cyan*	**-0.198**	**0.204**	**0.325**	0.131	1.000		
*Daph*	**-0.282**	**0.313**	**0.533**	0.012	0.079	1.000	
*Lept*	-0.159	0.164	0.153	0.062	-0.007	**0.263**	1.000

Statistically significant correlations are bolded. Infl–water inflow; Rete–water retention time; Temp–water temperature; Chl*a*–Chlorophyll *a* concentration; Cyan–cyanobacteria biomass; Daph–*Daphnia longispina* density; Lept–*Leptodora kindtii* density

For cluster CL-StSm (stable/summer), both the first and second canonical correlations were statistically significant (0.666; p<0.01 and 0.391; p<0.01, respectively). The highest contributions to the U1 and V1 canonical roots had a retention time and cyanobacterial biomass with canonical weights of 1.911 and 0.656, respectively. Water inflow (1.730) and *D*. *longispina* density (-0.972) were the main contributors to the U2 and V2 canonical roots. In turn, the highest loading for the U1 and V1 canonical roots was the chlorophyll *a* concentration (0.840) and cyanobacterial biomass (0.919), respectively, and for the U2 and V2 canonical roots was water inflow (0.742) and *L*. *kindtii* density (0.633), respectively (Table 4). For the two significant canonical correlations, 27.4% of the biotic factor variance was explained by predictor variables. Contrary to cluster CL-ExSp, in cluster CL-StSm, most of the correlation coefficients between abiotic and biotic factors were weak, and the correlation coefficients between biotic variables were much higher and reached the highest (negative) value (-0.640) for cyanobacterial biomass and *D*. *longispina* density (Table 4).

**Table 4:** Results of the canonical correlation analyses for cluster CL-StSm of the SOM output neurons.

Canonical roots for predictor variables		U1	U2	U3	U1	U2	U3
		Canonical weights			Canonical factor loadings		
*Infl*		-1.801	1.730	2.079	-0.118	0.742	0.227
*Rete*		-1.911	1.005	1.783	-0.067	-0.639	-0.098
*Temp*		-0.089	-0.575	0.761	0.138	-0.563	0.776
*Chla*		0.800	0.418	0.309	0.840	0.081	0.364
Canonical roots for response variables		**V1**	**V2**	V3	V1	V2	V3
		Canonical weights			Canonical factor loadings		
*Cyan*		0.656	-0.848	0.816	0.919	-0.385	0.087
*Daph*		-0.475	-0.972	0.726	-0.873	-0.346	0.343
*Lept*		0.139	0.534	0.892	-0.130	0.633	0.763
Canonical correlations		**0.666**	**0.391**	0.194			
Redundancy of response variables		24.02%	3.41%	0.89%			
	Correlations between variables						
	*Infl*	*Rete*	*Temp*	*Chla*	*Cyan*	*Daph*	*Lept*
*Infl*	1.000						
*Rete*	**-0.959**	1.000					
*Temp*	-0.103	0.085	1.000				
*Chla*	**-0.200**	**0.155**	**0.254**	1.000			
*Cyan*	**-0.180**	0.054	**0.182**	**0.508**	1.000		
*Daph*	-0.016	0.119	0.047	**-0.476**	**-0.640**	1.000	
*Lept*	**0.228**	**-0.167**	-0.036	0.001	**-0.297**	**0.157**	1.000

Statistically significant correlations are bolded. Infl–water inflow; Rete–water retention time; Temp–water temperature; Chl*a*–Chlorophyll *a* concentration; Cyan–cyanobacteria biomass; Daph–*Daphnia longispina* density; Lept–*Leptodora kindtii* density

For cluster CL-ExSm (extreme/summer), all three canonical correlations were significant (0.678; p<0.01, 0.560; p<0.01, and 0.236; p<0.05, respectively). The highest contributions to the U1 and V1 canonical roots had a water inflow and cyanobacterial biomass with canonical weights of -0.667 and 0.776, respectively. Water inflow (-0.957) and *L*. *kindtii* density (-0.901) were the main contributors to the U2 and V2 canonical roots, while temperature (-0.862) and *D*. *longispina* density (-1.060) were the main contributors to the U3 and V3 canonical roots, respectively. Similar to the canonical weights, the highest canonical factor loadings for U1 and V1 belonged to water inflow (0.715) and cyanobacterial biomass (0.925). In turn, the highest canonical factor loading was inflow (-0.689) for U2 and *D*. *longispina* density (-0.639) for V2. For U3 and V3, the highest factor loadings were found for temperature (-0.681) and *D*. *longispina* density (-0.752), respectively (Table 5).

**Table 5:** Results of the canonical correlation analyses for cluster CL-ExSm of the SOM output neurons.

Canonical roots for predictor variables		U1	U2	U3	U1	U2	U3
		Canonical weights			Canonical factor loadings		
*Infl*		-0.667	-0.957	-0.031	-0.715	-0.689	-0.102
*Rete*		-0.074	-0.168	0.151	0.698	0.589	0.118
*Temp*		0.458	-0.345	-0.862	0.648	-0.340	-0.681
*Chla*		0.458	-0.566	0.709	0.608	-0.568	0.552
Canonical roots for response variables		**V1**	**V2**	**V3**	V1	V2	V3
		Canonical weights			Canonical factor loadings		
*Cyan*		0.776	-0.798	0.514	0.925	-0.377	-0.038
*Daph*		-0.156	-0.278	-1.060	-0.159	-0.639	-0.752
*Lept*		-0.333	-0.901	0.836	-0.771	-0.579	0.265
Canonical correlations		**0.678**	**0.560**	**0.236**			
Redundancy of response variables		22.59%	9.26%	1.19%			
	Correlations between variables						
	*Infl*	*Rete*	*Temp*	*Chla*	*Cyan*	*Daph*	*Lept*
*Infl*	1.000						
*Rete*	**-0.938**	1.000					
*Temp*	-0.162	**0.184**	1.000				
*Chla*	-0.097	0.136	**0.209**	1.000			
*Cyan*	**-0.302**	**0.312**	**0.484**	**0.496**	1.000		
*Daph*	**0.342**	**-0.307**	**0.173**	0.040	0.122	1.000	
*Lept*	**0.591**	**-0.548**	**-0.271**	-0.099	**-0.505**	**0.293**	1.000

Statistically significant correlations are bolded. Infl–water inflow; Rete–water retention time; Temp–water temperature; Chl*a*–C lorophyll *a* concentration; Cyan–cyanobacteria biomass; Daph–*Daphnia longispina* density; Lept–*Leptodora kindtii* density

For the all three canonical correlations, the predictor variables explained 33% of the variance of biotic factors. All correlations between abiotic and biotic factors were significant. Among the biotic factors, the highest (negative) correlation was found for *L*. *kindtii* density and cyanobacterial biomass (-0.505) (Table 5).

## Discussion

In this study, we used an SOM to distinguish fairly homogeneous classes (clusters and sub-clusters) of sampling terms with similar biotic states, which then enabled the identification of the crucial abiotic factors responsible for the seasonal sequence of events. This allows us to now discuss the main patterns of seasonal variations in *D*. *longispina*, *L*. *kindtii* and cyanobacteria observed in our study in relation to the steps proposed by the classical model of plankton succession, the PGE model. The conceptual framework for this model concerning the seasonal dynamics of plankton communities in an idealized temperate lake was developed by an international group of limnologists, the Plankton Ecology Group (PEG), in the 1980s. The original PEG model described the roles of different abiotic and biotic factors driving the succession of zoo- and phytoplankton in 24 sequential steps [47].

The PEG model is mainly based on the temporal sequences and assumes the progressive growth of zooplankton during spring, when small, fast-growing algae are available [47]. However, in spite of a spring crop of algae (median chlorophyll *a* concentration of 10.52–13.24 μg dm^-3^), the abundance of *D*. *longispina* remained at a very low level in our study during early spring (sub-cluster ESp; Figs 6–8). The CCA analysis indicated that abiotic conditions (water temperature and hydrological regime) were key for the development of *Daphnia* in spring in the Sulejow Reservoir (Table 3). In reservoirs, increases in water levels are associated with varying dynamics in the zooplankton population [48] due to both hydrological advection and an increased concentration of suspended sediments [49]. Instability in water depth may change the underwater light climate as well as the nutrient dynamics, which both affect phytoplankton biomass and species composition [50] and can indirectly modify the succession of daphniids [51]. High water inflow conditions directly remove the large-sized species of cladocerans and copepods and favour the development of rotifers [52]. Such flushing effects on macrozooplankton may be periodically stronger than the top-down control exerted by planktivores [27]. In the Sulejow Reservoir, the negative effect of the hydrological conditions (high inflow and short retention time) most likely determined the very low density of *D*. *longispina* in April and May (Table 3). Unfortunately, the hydrological regime, which is an important driver of plankton seasonality in dam reservoirs, was not included in the PEG model focused on lakes. Moreover, the model (even in the updated version [53]) does not consider the temperature as the crucial factor for zooplankton spring phenology [53, 54], even though the close dependence of daphniid population dynamics on epilimnetic water temperatures during spring has been documented by many authors [21, 55]. In addition, some recent analyses of long-term data have indicated that spring development of *Daphnia* has mainly been regulated by temperature and not by food availability [56]. The temperature directly (via changes in life processes) and indirectly (via changes in habitat properties) influences *Daphnia* spp. populations [56]. The response of *Daphnia* to temperature variability depends on species-specific thermal tolerance ranges. Previous observations of *D*. *longispina* under natural conditions in a temperate zone indicated that this species became highly abundant at temperatures above 14°C, and their favourable temperature range was 15–21°C [57]. In terms grouped in sub-cluster ESp (“early spring”), the temperature reached a median of 13.86°C. Thus, our results confirmed that the very low water temperature (< 14°C) was another critical factor in the development of the *Daphnia* population in early spring, in addition to hydrology (Fig 7). The terms of sub-cluster Sp (“spring”) were characterised not only by a raised temperature (median range for the three stations of 17.80–18.19°C) but also by a lower inflow (median of 30.57 m^3^ s^-1^) and longer retention time (median of 28 days) compared to ESp. Because the chlorophyll *a* concentrations in Sp were comparable to those in sub-cluster ESp, our results indicate that the increase in *Daphnia* density in spring (May–June) was still determined by abiotic conditions: an increase in water temperature and more stable hydrological conditions compared with early spring (Figs 7 and 8; Table 3).

One of the most important sequential statements of the seasonal succession of plankton is the “clear water phase” (CWP) that usually occurs in late spring. The PEG model describes the CWP as a decline in phytoplankton biomass towards a mid-season biomass minimum due to the intensive grazing pressure of zooplankton [47]. This event is connected with a high peak of planktonic herbivores, especially daphniids and copepods [58]. A similar pattern was observed in our study: the highest density of *Daphnia* was observed in June [sampling terms grouped in LSp/Sm (“late spring/summer”)] (Fig 8), i.e., at the same time as the appearance of the lowest concentration of chlorophyll *a* in stations BR and TR (median of 3.40 μg dm^-3^ for BR and 5.68 μg dm^-3^ for TR). The low concentration of chlorophyll *a* in late spring or early summer, i.e., when *Daphnia* populations were observed to be at their highest densities, could be attributed to the grazing of *Daphnia* on algal biomass, resulting in the spring clear-water phase (CWP). This phenomenon, characteristic of the plankton phenology of many temperate lakes [47], has been regularly observed in the Sulejow Reservoir, which is a eutrophic (with high levels of nutrients during the spring), shallow and polymictic ecosystem [2]. Thus, CWP development appears to be regulated in the reservoir mainly by selective grazing of *Daphnia* on small-sized algal populations, rather than by thermal stratification or seasonal changes in nutrient concentrations [59]. The duration of the CWP in the Sulejow Reservoir differed between years and was usually terminated by a collapse in the *Daphnia* abundance and the intensive expansion of cyanobacteria [35], as was also recorded for sampling terms in sub-cluster Sm (“summer”) (Figs 6 and 8).

The terms of sub-cluster Sm (“summer”) (September and July) were characterised by the lowest inflow, the longest retention time and a high median water temperature (> 20°C; Figs 6 and 7). Consequently, the analysed abiotic factors were completely different than in the sampling terms grouped in sub-clusters ESp, Sp and LSp/Sm corresponding to spring conditions. A stable hydrological regime and high temperature may increase the importance of biotic interactions [19], which is consistent with the assumptions of the PEG model [47]. In eutrophic lakes and reservoirs, such abiotic conditions are favourable for cyanobacterial growth [28]. We observed a clear relationship between cyanobacterial biomass and hydrological conditions in a spatial gradient of terms grouped in sub-cluster Sm (“summer”); blooms were more frequent at the TR and BR stations than at the ZA station (Figs 2, 4 and 8). However, in a temporal gradient, the highest cyanobacterial biomass appeared at the highest temperatures and in the most stable hydrological conditions represented by sub-cluster HotSm (“hot summer”) (June, August) (Table 2). Cyanobacteria generally grow better at higher temperatures (above 20°C) than other phytoplankton organisms, which gives them a competitive advantage in warm periods [25]. Apart from rising temperatures and nutrient concentrations, stable hydrological conditions are recognised as one of the most important factors in determining the development of bloom-forming cyanobacteria [60, 61]. An increased retention time favours cyanobacterial dominance [62], which was also confirmed by our analyses (CCA, Table 4). Analyses of the correlation matrices revealed that the abundance of cyanobacteria was closely dependent on abiotic factors (temperature and hydrology) within all seasons in the studied years (Tables 3–5).

In the summer months (sub-clusters Sm and HotSm), the dynamics of the *D*. *longispina* population were dependent on biotic factors, mostly cyanobacteria. Cyanobacteria are well-known poor food sources for daphniids due to their low nutrient content, toxicity and colony and filament formation [63, 64]. A negative correlation between the density of daphniids and the biomass of toxin-producing cyanobacteria has been previously reported [65, 66]. The PEG model also assumed growth limitation of herbivore crustaceans resulting from the dominance of bloom-forming, less edible algae or cyanobacteria during summer [47]. In our study, the sampling terms associated with sub-cluster Sm (“summer”) were characterised by a high cyanobacterial biomass and a low density of *D*. *longispina* (Fig 8). Indeed, a strong negative correlation between *D*. *longispina* and cyanobacteria in the CL-StSm cluster was found (Table 4). We suppose that the negative impact of cyanobacteria on *D*. *longispina* resulted from their low nutritional value or to mechanical damage to the filtering apparatus from their colonies and filaments [67], rather than to cyanobacterial toxins. These conclusions are based on our previous studies indicating that generations of daphniids from the Sulejow Reservoir contained effective antioxidant systems to protect themselves against the accumulation of cyanobacterial toxins and their harmful effects [13, 14]. In the Sulejow Reservoir, the concentrations of microcystins reached an average of 5.83 μg dm^-3^, and, in the areas of the bloom, the concentrations were recorded at levels up to 22–30 μg dm^-3^ [2]. Despite this observation, the population of *D*. *longispina* was eventually reduced in abundance, but it did not completely decline during the blooms (as presented in this study and [14]).

In the PEG model, the effects of higher trophic levels on herbivorous zooplankton are only restricted to fish predation [47]. In addition, the updated version of the model [53] does not include the impact of invertebrate predators on filter feeders. However, our previous study on the Sulejow Reservoir revealed that the abundance of daphniids in the summer months was more dependent on *L*. *kindtii* predation than on fish pressure [17, 39]. In June–July, *Leptodora*, due to their high densities in the pelagic zone, were able to eliminate 10–51% of *Daphnia* biomass, whereas fish eliminated only 0.1–5.4% [17]. The predatory rate of *L*. *kindtii* depends on temperature [21] and the density of the prey population [18]. Therefore, the density and predatory pressure of *Leptodora* increased during a period with a relatively high abundance of potential prey (*Daphnia*). This effect was particularly evident during summer months at the ZA and BR stations (Fig 3). The SOM showed that both the spatial distribution (in particular stations) and the temporal dynamics of daphniids and *L*. *kindtii* were similar in sampling terms grouped in sub-cluster HunLSp/Sm (“hydrologically unstable late spring/summer”) (Fig 8). A comparable sequence of growth and decline in the densities of these animals corresponded to the classical coupled system of predator and prey oscillations observed in other lakes [68]. Thus, the SOM results confirmed that the abundance of *Daphnia* was regulated by the predation of *L*. *kindtii* during summer. However, because the peaks in *Leptodora* density were short in duration and their frequency was different within the studied years (Fig 3), the precise estimation of *L*. *kindtii* pressure on daphniid population dynamics should be measured individually for each season. Analysis of the correlation matrices revealed that *Leptodora*, similar to *Daphnia*, avoided summer blooms. Interestingly, there was also a positive influence of increased inflow on *L*. *kindtii* density (Table 5). The positive correlation between inflow and *L*. *kindtii* density might be related to the increased turbidity of the water during periods of intensive flow. Zettler and Carter [69] observed that a reduction in transparency protected this large cladoceran from visually seeking fish predators and contributed to its increased abundance in Lake Temiskaming. Moreover, in reservoirs, the raised concentration of suspended organic matter caused by inflow is often associated with peaks in bacteria, protists and their consumers, such as *Bosmina* spp. [70]. These small cladocerans can be good food resources for *L*. *kindtii* [71]. Our assumptions, however, require confirmation by further detailed research.

In this paper, we analysed and discussed the role of selected mechanisms that drive temporal changes in the structure of plankton communities. However, a more comprehensive study of the Sulejow Reservoir may be necessary for expanding the protection and restoration strategies for this eutrophic ecosystem. Thus, further research projects focused on light availability and nutrient uptake as determinants of phytoplankton growth, the effect of juvenile fish on zooplankton during summer and the importance of the microbial food web for herbivorous zooplankton community dynamics have been developed.

## Conclusions

In conclusion, we recommend SOMs as an appropriate method for the analysis of complex patterns in reservoir habitats. We believe that our results are important in providing a better understanding of *L*. *kindtii*-*Daphnia*-cyanobacteria interactions in an ecosystem that is highly dependent on the hydrological regime. Long-term studies generate comprehensive and detailed databases, which, when analysed by the appropriate mathematical approaches, can reveal ecosystem patterns, dynamics and control processes. The results of long-term research provide knowledge to the broader scientific community concerning the function of aquatic ecosystems. They are also essential in the development of management and policy decisions regarding the protection of ecosystems [72]. The application of an SOM in our research allowed the determination of a cause-and-effect understanding of the relationship between selected biotic and abiotic parameters. The importance of hydrology (flow rate, retention time, water-level fluctuations, etc.) as a plankton-regulating factor may be essential for the formulation of management decisions in dam reservoirs. This mainly refers to the regulation of retention time with respect to the control of the seasonal dynamics of large grazers, such as *Daphnia* spp., and/or as a tool for decreasing the occurrence of algal blooms. This method is especially recommended for anthropogenically modified ecosystems with toxic cyanobacterial blooms [28].

## Supporting Information

S1 Fig The 412 sampling terms assigned to the 24 SOM output neurons. The code for each term consists of two digits for the day, two digits for the month and two digits for the year of sampling, e.g., 190799 = 19^th^ of July 1999.
(TIF)

S1 Table Database of the self-organizing maps (SOM). (DOCX)

S2 Table Self-organizing maps of different calculated sizes, quantization and topographic errors and the number of empty neurons (which were not appreciated). (DOCX)

## References

[1] Thornton J, Steel A, Rast W. Reservoirs (Chapter 8) In:. Chapman D, editor. Water Quality Assessments—A Guide to Use of Biota, Sediments and Water in Environmental Monitoring—Second Edition. UNESCO/WHO/UNEP, Printed in Great Britain at the University Press, Cambridge; 1996 pp. 371–411.

[2] Wagner I, Izydorczyk K, Kiedrzyńska E, Mankiewicz-Boczek J, Jurczak T, Bednarek A, et al Ecohydrological system solution for enhancement of ecosystem services: the Pilica river demonstration project. Ecohydrol Hydrobiol. 2009; 9: 13–39.

[3] Persson J, Brett MT, Verde T, Ravet JL. Food quantity and quality regulation of trophic transfer between primary producers and a keystone grazer (*Daphnia*) in pelagic freshwater food webs. Oikos. 2007; 116: 1152–1168.

[4] Jürgens K. Impact of *Daphnia* on planktonic microbial food webs–review. Mar Microb Food Webs. 1994; 8: 295–324.

[5] Lampert W, Sommer U. Limnoecology: the ecology of lakes and streams. Oxford University Press, New York; 1997.

[6] Sommer F, Santer B, Jamieson C, Hansen T, Sommer U. *Daphnia* population growth but not moulting is a substantial phosphorus drain for phytoplankton. Freshw Biol. 2003; 48:67–74.

[7] Ebert D. Ecology, Epidemiology and Evolution of Parasitism in *Daphnia* Bethesda (MD): National Library of Medicine (US), National Center for Biotechnology Information Available: http://www.ncbi.nlm.nih.gov/entrez/query.fcgi?db=Books. 2005; Available: http://www.ncbi.nlm.nih.gov/corehtml/pmc/homepages/bookshelf/pdf/daph_screenUS.pdf

[8] Lampert W. *Daphnia*: Development of a model organism in ecology and evolution International Ecology Institute, Oldendorf/Luhe, Germany; 2011.

[9] Wetzel RG. Limnology: Lake and River Ecosystems, 3rd Edition Academic Press; 2001.

[10] Jurczak T, Tarczyńska M, Izydorczyk K, Mankiewicz J, Zalewski M, Meriluoto J. Elimination of microcystins by water treatment process–examples from Sulejow Reservoir, Poland. Water Res. 2005; 39: 2394–2406. 15927226 10.1016/j.watres.2005.04.031

[11] Fulton RS, Paerl HW. Toxic and inhibitory effects of the blue green alga *Microcystis aeruginosa* on herbivorous zooplankton. J Plankton Res. 1987; 9: 837–855.

[12] DeMott WR. Foraging strategies and growth inhibition in five daphniids feeding on mixtures of a toxic cyanobacterium and a green alga. Freshwat Biol. 1999; 42: 263–274.

[13] Wojtal-Frankiewicz A, Bernasińska J, Jurczak T, Gwoździński K, Frankiewicz P, Wielanek M. Microcystin assimilation and detoxification by *Daphnia* spp. in two ecosystems of different cyanotoxin concentrations. J Limnol. 2013; 72: 154–171.

[14] Wojtal-Frankiewicz A, Bernasińska J, Frankiewicz P, Gwoździński K, Jurczak T. Response of *Daphnia*’s antioxidant systems to spatial heterogeneity in cyanobacteria concentrations in a lowland reservoir. PLoS ONE 2014; 9(11): e112597 10.1371/journal.pone.0112597 25380273 PMC4224506

[15] Harris GP. Phytoplankton Ecology: Structure, Function and Fluctuation, 1st edn. Chapman & Hall, London; 1986.

[16] Vörös L, Padisak J. Phytoplankton biomass and chlorophyll a in some shallow lakes in central Europe. Hydrobiologia. 1991; 215: 111–119.

[17] Wojtal A, Frankiewicz P, Wagner-Łotkowska I, Zalewski M. The evaluation of the role of pelagic invertebrate versus vertebrate predators on the seasonal dynamics of filtering Cladocera. Hydrobiologia. 2004; 515: 123–135.

[18] Herzig A, Auer B. The feeding behaviour of *Leptodora kindti* and its impact on the zooplankton community of Neusiedler See (Austria). Hydrobiologia. 1990; 198: 107–117.

[19] Zalewski M. Ecohydrology–the use of ecological and hydrological processes for sustainable management of water resources. Hydrolog Sci J. 2002; 47: 823–832.

[20] Zalewski M, Naiman RJ. The regulation of riverine fish communities by continuum of abiotic-biotic factors In: Alabaster JS, editor. Habitat Modification and Freshwater Fisheries. FAO/UN/Butterworths Scientific Ltd, London; 1985 pp. 3–9.

[21] Wagner A, Benndorf J. Climate-driven warming during spring destabilises a *Daphnia* population: a mechanistic food web approach. Oecologia. 2007; 151: 351–364. 17120058 10.1007/s00442-006-0554-5

[22] Adrian R, Wilhelm S, Gerten D. Life-history traits of lake plankton species may govern their phenological response to climate warming. Glob Change Biol. 2006; 12: 652–661.

[23] Chen CY, Folt CL. Consequences of fall warming for zooplankton overwintering success. Limnol Oceanogr. 1996; 41: 1077–1086.

[24] Benndorf J, Kranich J, Mehner T, Wagner A. Temperature impact on the midsummer decline of *Daphnia galeata*: an analysis of long-term data from the biomanipulated Bautzen Reservoir (Germany). Freshwat Biol. 2001; 46: 199–211.

[25] Jöhnk KD, Huisman J, Sharples J, Sommeijer B, Visser PM, Stroom JM. Summer heat waves promote blooms of harmful Cyanobacteria. Glob Change Biol. 2008; 14: 495–512.

[26] Wojtal A, Bogusz D, Menshutkin V, Izydorczyk K, Frankiewicz P, Wagner-Łotkowska I, et al A study of *Daphnia*-*Leptodora*-juvenile Percids interactions using a mathematical model in the biomanipulated Sulejow Reservoir. Ann Limnol–Int J Lim. 2008; 44: 69–86.

[27] Renella AM, Quirós R. The effects of hydrology on plankton biomass in shallow lakes of the Pampa Plain. Hydrobiologia. 2006; 556: 181–191.

[28] Paerl HW . Mitigating harmful cyanobacterial blooms in a human and climatically impacted world. Life. 2014; 4: 988–1012. 10.3390/life4040988 25517134 PMC4284478

[29] Carlotti F, Giske J, Werner F. Modeling zooplankton dynamics In: Harris R, Wiebe P, Lenz J, Skjoldal HR, Huntley M, editors. ICES Zooplankton Methodology Manual. Academic Press; 2000 pp. 571–667.

[30] Brosse S, Giraudel JL, Lek S. Utilisation of non-supervised neural networks and principal component analysis to study fish assemblages. Ecol Model. 2001; 146: 159–166.

[31] Quinn GP, Keough MJ. Experimental Design and Data Analysis for Biologists. Cambridge University Press, Cambridge; 2002.

[32] Kohonen T. Self-organized formation of topologically correct feature maps. Biol Cyber. 1982; 43: 59–69.

[33] Lek S, Scardi M, Verdonschot PFM, Descy JP, Park YS. Modelling community structure in freshwater ecosystems Springer, Berlin; 2005.

[34] Mankiewicz-Boczek J, Izydorczyk K, Romanowska-Duda Z, Jurczak T, Stefaniak K, Kokocinski M. Detection and monitoring toxigenicity of cyanobacteria by application of molecular methods. Env Toxicol. 2006; 21: 380–387. 16841323 10.1002/tox.20200

[35] Izydorczyk K, Skowron A, Wojtal A, Jurczak T. The stream inlet to a shallow bay of a drinking water reservoir, a “hot-spot” for *Microcystis* blooms initiation. Int Rev Hydrobiol. 2008; 93: 257–268.

[36] Wojtal A. Analysis of the trophic interactions connected with cascading effect in littoral and pelagic zone of the Sulejow Reservoir. Ph.D. Thesis (in Polish), University of Lodz, Poland. 2000.

[37] Frankiewicz P, Dąbrowski K, Martyniak A, Zalewski M. Cannibalism as a regulatory force of pikeperch, *Stizostedion lucioperca* (L.), population dynamics in the lowland Sulejow Reservoir (Central Poland). Hydrobiologia. 1999; 408/409: 47–55.

[38] Komarkowa J, Vyhnalek V, Kubecka J. Impact of fishstock manipulation on the composition of net phytoplankton in the Rimov Reservoir (Czech Republic). Wat Scien Technol. 1995; 32: 207–216.

[39] Wojtal A, Frankiewicz P, Zalewski M. The role of the invertebrate predator *Leptodora kindti* in the trophic cascade of a lowland reservoir. Hydrobiologia. 1999; 416: 215–223.

[40] Benzie JAH. The genus *Daphnia* (including *Daphniopsis*) (Anomopoda: Daphniidae) In: Dumont HJF, editor. Guides to the Identification of the Macroinvertebrates of the Continental Waters of the World Kenobi Productions, Ghent; Backhuys Publishers, Leiden; 2005 pp. 1–374.

[41] Lawton L, Marsalek B, Padisak J, Chorus I. Determination of Cyanobacteria in the laboratory In: Chorus I, Bartram J, editors. Toxic Cyanobacteria in water. A guide to their public health consequences, monitoring and management. E&FN Spon; 1999 pp. 347–367.

[42] Vesanto J, Himberg J, Alhoniemi E, Parhankangas J. SOM Toolbox for Matlab 5. Report A57 [Internet]. Helsinki University of Technology, Helsinki, Finland 2000 Available: http://www.cis.hut.fi/somtoolbox/package/papers/techrep.pdf

[43] Céréghino R, Park YS. Review of the Self-Organizing Map (SOM) approach in water resources: Commentary. Environ Modell Softw. 2009; 24: 945–947.

[44] Kohonen T. Self-organizing maps, 3rd ed. Springer, Berlin; 2001.

[45] Vesanto J, Alhoniemi E. Clustering of the self-organizing map. IEEE T Neural Networ. 2000; 11: 586–600. 10.1109/72.846731 18249787

[46] Zar JH. Biostatistical Analysis. Prentice-Hall, Inc., Englewood Cliffs, New Jersey; 1984.

[47] Sommer U, Gliwicz ZM, Lampert W, Dunkan A. The PEG-model of seasonal succession of planktonic events in fresh waters. Arch Hydrobiol. 1986; 106: 433–471.

[48] Lehman JT, Platte RA, Ferris JA. Role of hydrology in development of a vernal clear water phase in an urban impoundment. Freshwat Biol. 2007; 52: 1773–1781.

[49] Wolfinbarger WC. Influence of biotic and abiotic factors on seasonal succession of zooplankton in Hugo Reservoir, Oklahoma, U.S.A. Hydrobiologia. 1999; 400: 13–31.

[50] Kimmel BL, Lind OT, Paulson LJ. Reservoir Primary Production In: Thornton KW, Kimmel BL, Payne FE, editors. Reservoir Limnology: Ecological Perspectives. John Wiley & Sons, New York; 1990 pp. 133–193.

[51] Naselli-Flores L, Barone R. Importance of water-level fluctuation on population dynamics of cladocerans in a hypertrophic reservoir (Lake Arancio, south-west Sicily, Italy). Hydrobiologia. 1997; 360: 223–232.

[52] Godlewska M, Mazurkiewicz-Boroń G, Pociecha A, Wilk-Woźniak E, Jelonek M. Effects of flood on the functioning of the Dobczyce reservoir ecosystem. Hydrobiologia. 2003; 504: 305–313.

[53] Sommer U, Adrian R, De Senerpont Domis L, Elser JJ, Gaedke U, Ibelings B, et al Beyond the Plankton Ecology Group (PEG) model: mechanisms driving plankton succession. Annu Rev Ecol Evol Syst. 2012; 43: 429–448.

[54] Straile D . Zooplankton biomass dynamics in oligotrophic versus eutrophic conditions: a test of the PEG model Freshwat Biol. 2015; 60: 174–183.

[55] Schalau K, Rinke K, Straile D, Peeters F. Temperature is the key factor explaining interannual variability of *Daphnia* development in spring: a modeling study. Oecologia. 2008; 157: 531–543. 10.1007/s00442-008-1081-3 18574598

[56] Wojtal-Frankiewicz A. The effects of global warming on *Daphnia* spp. population dynamics: a review. Aquat Ecol. 2012; 46: 37–53.

[57] Verbitsky VB, Verbitskaya TI. Effects of constant and stepwise changes in temperature on the species abundance dynamics of four Cladocera species. Knowl Manag Aquat Ec. 2011; 402: 03 Available: http://www.kmae-journal.org/articles/kmae/pdf/2011/03/kmae100081.pdf

[58] Talling JF. Phytoplankton–zooplankton seasonal timing and the ‘clear-water phase’ in some English lakes. Freshwat Biol. 2003; 48: 39–52.

[59] Dröscher I, Finlay K, Patoine A, Leavitt PR. *Daphnia* control of the spring clear-water phase in six polymictic lakes of varying productivity and size. Verh Internat Verein Limnol. 2008; 30: 186–190.

[60] Elliott JA. The seasonal sensitivity of Cyanobacteria and other phytoplankton to changes in flushing rate and water temperature. Glob Change Biol. 2010; 16: 864–876.

[61] Roelke DL, Pierce RH. Effects of inflow on harmful algal blooms: some considerations. J Plankton Res. 2011; 33: 205–209.

[62] Paerl H. Nutrient and other environmental controls of harmful Cyanobacterial blooms along the freshwater-marine continuum. In: Hudnell KE, editor. Cyanobacterial harmful algal blooms: State of science and research needs. Chapter 10. Adv Exp Med Biol. 2008; 619: 217–237. 10.1007/978-0-387-75865-7_10 18461771

[63] Rohrlack T, Henning M, Khol JG. Mechanisms of the inhibitory effect of the cyanobacterium *Microcystis aeruginosa* on *Daphnia galeata*’s ingestion rate. J Plankton Res. 1999; 21: 1489–1500.

[64] Müller-Navarra DC, Brett MT, Liston AM, Goldman CR. A high unsaturated fatty acid predicts carbon transfer between primary producers and consumers. Nature. 2000; 403: 74–76. 10638754 10.1038/47469

[65] Ferrão-Filho AS, Domingos P, Azevedo SMFO. Influences of a *Microcystis aeruginosa* Kützing bloom on zooplankton populations in Jacarepaguá Lagoon (Rio de Janeiro, Brazil). Limnologica. 2002; 32: 295–308.

[66] Reichwaldt ES, Song H, Ghadouani A. Effects of the distribution of a toxic *Microcystis* bloom on the small scale patchiness of zooplankton. PLoS ONE 2013; 8(6), e66674 10.1371/journal.pone.0066674 23840516 PMC3686710

[67] Bednarska A, Pietrzak B, Pijanowska J. Effect of poor manageability and low nutritional value of cyanobacteria on *Daphnia magna* life history performance. J Plankton Res. 2014; 36: 838–847.

[68] Lunte CC, Luecke C. Trophic Interactions of *Leptodora* in Lake Mendota. Limnol Oceanogr. 1990; 35: 1091–1100.

[69] Zettler ER, Carter JCH. Zooplankton community and species responses to a natural turbidity gradient in Lake Temiskaming, Ontario–Quebec. Can J Fish Aquat Sci. 1986; 43: 665–673.

[70] Straškrábová V, Šimek K, Vrba J. Long-term development of reservoir ecosystems—changes in pelagic food webs and their microbial component. Limnetica. 2005; 24: 9–20.

[71] Branstrator DK, Lehman JT. Invertebrate predation in Lake Michigan: regulation of *Bosmina longirostris* by *Leptodora kindtii* . Limnol Oceanogr. 1991; 36: 483–495.

[72] Lindenmayer DB, Likens GE, Andersen A, Bosman D, Bull CM, Burns E, et al Value of long-term ecological studies. Austral Ecol. 2012; 37: 745–757.

