# Supplementary figures and images for: Long-Term Patterns in the Population Dynamics of Daphnia longispina, Leptodora kindtii and Cyanobacteria in a Shallow Reservoir: A Self-Organising Map (SOM) Approach
Source: PLoS One. 2015 Dec 3;10(12):e0144109. doi: 10.1371/journal.pone.0144109 (PMC4669109; doi:10.1371/journal.pone.0144109)

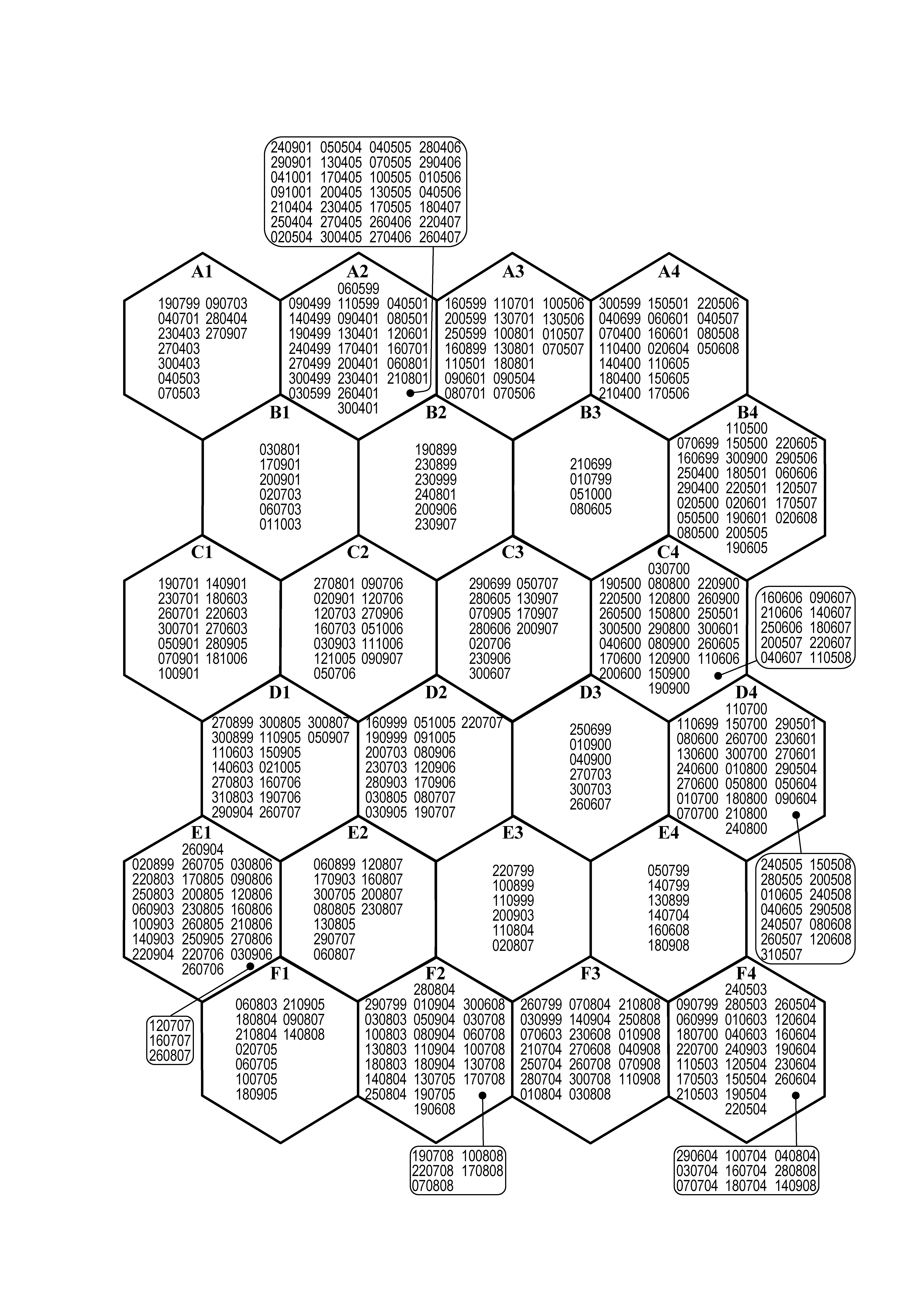

Supplement: S1 Fig — The code for each term consists of two digits for the day, two digits for the month and two digits for the year of sampling, e.g., 190799 = 19th of July 1999. (TIF) [file pone.0144109.s001.tif]
