# Supplementary material for: Long-Term Patterns in the Population Dynamics of Daphnia longispina, Leptodora kindtii and Cyanobacteria in a Shallow Reservoir: A Self-Organising Map (SOM) Approach
Source: PLoS One. 2015 Dec 3;10(12):e0144109. doi: 10.1371/journal.pone.0144109 (PMC4669109; doi:10.1371/journal.pone.0144109)
Supplement: S1 Table — (DOCX) [file pone.0144109.s002.docx]

**S1 Table. Data base of SOM.**

| Subcl | Code of date | Inflow | Ret time | TRtem | BRtem | ZAtem | TRchl | BRchl | ZAchl | TRcya | BRcya | ZAcya | TRdap | BRdap | ZAdap | TRlep | BRlep | ZAlep |
| --- | --- | --- | --- | --- | --- | --- | --- | --- | --- | --- | --- | --- | --- | --- | --- | --- | --- | --- |
| 1 | 90499 | 26.80 | 32.84 | 11.74 | 10.66 | 11.28 | 5.13 | 54.98 | 13.60 | 0.19 | 0.77 | 0.31 | 5.00 | 1.20 | 0.00 | 0.00 | 0.00 | 0.00 |
| 1 | 140499 | 27.90 | 31.54 | 11.22 | 10.24 | 10.58 | 4.10 | 43.37 | 12.35 | 0.15 | 0.68 | 0.28 | 4.25 | 0.62 | 0.00 | 0.00 | 0.00 | 0.00 |
| 1 | 190499 | 27.00 | 32.60 | 10.19 | 9.41 | 9.19 | 2.10 | 20.17 | 9.85 | 0.07 | 0.50 | 0.22 | 2.75 | 0.31 | 0.00 | 0.00 | 0.00 | 0.00 |
| 1 | 240499 | 36.80 | 23.92 | 9.68 | 9.00 | 8.50 | 1.08 | 8.57 | 8.60 | 0.04 | 0.41 | 0.19 | 2.00 | 0.00 | 0.00 | 0.00 | 0.00 | 0.00 |
| 1 | 270499 | 35.80 | 24.58 | 10.84 | 10.50 | 10.25 | 5.95 | 14.75 | 17.20 | 0.22 | 0.26 | 0.38 | 1.35 | 0.85 | 0.00 | 0.00 | 0.00 | 0.00 |
| 1 | 300499 | 32.90 | 26.75 | 12.00 | 12.00 | 12.00 | 10.84 | 20.93 | 25.79 | 0.40 | 0.12 | 0.57 | 0.70 | 1.70 | 0.00 | 0.00 | 0.00 | 0.00 |
| 1 | 30599 | 28.90 | 30.45 | 12.39 | 12.89 | 12.75 | 10.90 | 18.49 | 34.38 | 0.21 | 0.11 | 0.29 | 2.60 | 1.48 | 0.17 | 0.00 | 0.00 | 0.00 |
| 1 | 60599 | 23.40 | 37.61 | 12.78 | 13.78 | 13.50 | 11.02 | 16.06 | 42.96 | 0.01 | 0.09 | 0.00 | 4.50 | 1.25 | 0.33 | 0.00 | 0.00 | 0.00 |
| 1 | 110599 | 22.90 | 38.43 | 13.52 | 14.37 | 13.98 | 11.05 | 14.84 | 47.25 | 0.33 | 0.07 | 0.01 | 5.08 | 3.52 | 0.49 | 0.00 | 0.00 | 0.00 |
| 2 | 160599 | 26.30 | 33.46 | 15.00 | 15.55 | 14.95 | 11.10 | 12.41 | 55.83 | 0.97 | 0.03 | 0.02 | 6.26 | 8.08 | 0.83 | 0.00 | 0.00 | 0.00 |
| 2 | 200599 | 26.70 | 32.96 | 15.74 | 16.14 | 15.44 | 11.20 | 11.20 | 60.13 | 1.30 | 0.01 | 0.03 | 6.85 | 10.36 | 1.00 | 0.00 | 0.00 | 0.00 |
| 2 | 250599 | 17.90 | 49.17 | 17.51 | 17.54 | 17.02 | 11.47 | 12.23 | 40.01 | 1.08 | 0.01 | 0.04 | 9.26 | 8.67 | 2.06 | 0.00 | 0.00 | 0.00 |
| 2 | 300599 | 18.20 | 48.36 | 21.05 | 20.35 | 20.19 | 12.01 | 14.30 | 17.87 | 0.64 | 0.00 | 0.06 | 14.08 | 5.29 | 4.18 | 0.00 | 0.00 | 1.00 |
| 2 | 40699 | 19.40 | 45.37 | 22.82 | 21.76 | 21.78 | 12.28 | 15.34 | 3.84 | 0.43 | 0.00 | 0.07 | 16.50 | 3.60 | 5.25 | 0.00 | 0.00 | 2.00 |
| 4 | 70699 | 21.30 | 41.32 | 22.32 | 22.27 | 21.84 | 14.02 | 17.42 | 9.59 | 0.37 | 0.03 | 0.71 | 19.75 | 6.55 | 7.37 | 0.00 | 2.00 | 3.00 |
| 4 | 110699 | 21.10 | 41.71 | 21.82 | 22.78 | 21.90 | 15.76 | 19.49 | 15.34 | 0.30 | 0.06 | 1.36 | 23.00 | 9.50 | 9.50 | 0.00 | 4.00 | 4.00 |
| 4 | 160699 | 26.80 | 32.84 | 21.67 | 21.64 | 21.02 | 20.11 | 14.72 | 41.88 | 0.27 | 0.13 | 1.03 | 13.72 | 31.63 | 18.19 | 0.00 | 2.00 | 2.00 |
| 2 | 210699 | 57.00 | 15.44 | 21.52 | 20.50 | 20.14 | 24.47 | 9.95 | 68.42 | 0.25 | 0.21 | 0.70 | 4.45 | 53.75 | 26.88 | 0.00 | 0.00 | 0.00 |
| 4 | 250699 | 160.80 | 5.47 | 19.42 | 16.30 | 15.90 | 16.59 | 16.17 | 20.32 | 2.54 | 0.09 | 0.77 | 1.80 | 17.82 | 20.38 | 0.00 | 3.00 | 3.00 |
| 4 | 290699 | 108.30 | 8.13 | 21.13 | 19.32 | 19.30 | 13.74 | 31.93 | 33.59 | 2.19 | 0.99 | 0.81 | 1.50 | 83.24 | 13.66 | 0.00 | 1.50 | 1.50 |
| 2 | 10799 | 78.90 | 11.15 | 22.84 | 22.34 | 22.70 | 10.89 | 47.69 | 46.86 | 1.85 | 1.88 | 0.85 | 1.20 | 148.67 | 6.94 | 0.00 | 0.00 | 0.00 |
| 6 | 50799 | 32.60 | 27.00 | 23.07 | 22.66 | 22.54 | 26.75 | 41.93 | 49.96 | 6.45 | 1.17 | 0.53 | 26.10 | 126.83 | 43.52 | 0.30 | 1.00 | 8.50 |
| 6 | 90799 | 54.20 | 16.24 | 23.30 | 22.98 | 22.38 | 42.61 | 36.18 | 53.07 | 11.05 | 0.46 | 0.20 | 51.00 | 105.00 | 80.10 | 0.60 | 2.00 | 17.00 |
| 6 | 140799 | 40.60 | 21.68 | 23.32 | 22.61 | 22.49 | 58.47 | 30.43 | 56.18 | 12.03 | 0.86 | 1.25 | 25.50 | 52.50 | 40.05 | 0.30 | 1.00 | 10.50 |
| 1 | 190799 | 37.90 | 23.22 | 23.34 | 22.24 | 22.60 | 74.33 | 24.67 | 59.28 | 13.00 | 1.25 | 2.30 | 0.00 | 0.00 | 0.00 | 0.00 | 0.00 | 4.00 |
| 5 | 220799 | 28.80 | 30.56 | 23.28 | 22.47 | 22.47 | 56.24 | 68.73 | 99.31 | 15.19 | 7.83 | 1.79 | 15.60 | 31.00 | 48.00 | 0.05 | 1.00 | 5.50 |
| 6 | 260799 | 27.50 | 32.00 | 23.22 | 22.77 | 22.35 | 38.15 | 112.79 | 139.33 | 17.39 | 14.42 | 1.28 | 31.20 | 62.00 | 96.00 | 0.10 | 2.00 | 7.00 |
| 5 | 290799 | 25.40 | 34.65 | 23.08 | 23.03 | 22.22 | 27.07 | 68.89 | 89.85 | 23.89 | 16.11 | 10.89 | 16.53 | 33.87 | 48.83 | 0.05 | 1.00 | 3.50 |
| 5 | 20899 | 21.30 | 41.32 | 22.94 | 23.30 | 22.10 | 16.00 | 24.99 | 40.37 | 30.39 | 17.81 | 20.50 | 1.87 | 5.74 | 1.67 | 0.00 | 0.00 | 0.00 |
| 5 | 60899 | 20.10 | 43.79 | 23.24 | 23.10 | 22.21 | 13.44 | 21.41 | 27.55 | 21.19 | 11.97 | 12.01 | 5.27 | 34.60 | 10.14 | 0.00 | 0.00 | 8.00 |
| 5 | 100899 | 19.30 | 45.60 | 23.54 | 22.90 | 22.32 | 10.89 | 17.84 | 14.74 | 11.99 | 6.13 | 3.51 | 8.67 | 63.46 | 18.62 | 0.00 | 0.00 | 16.00 |
| 6 | 130899 | 18.30 | 48.09 | 23.22 | 22.03 | 21.42 | 9.13 | 12.92 | 10.89 | 6.46 | 3.26 | 1.83 | 5.47 | 39.73 | 11.73 | 0.00 | 1.50 | 8.00 |
| 2 | 160899 | 17.10 | 51.47 | 22.90 | 21.16 | 20.52 | 7.37 | 8.01 | 7.05 | 0.93 | 0.38 | 0.14 | 2.28 | 16.00 | 4.84 | 0.00 | 3.00 | 0.00 |
| 2 | 190899 | 17.40 | 50.58 | 21.66 | 20.25 | 19.72 | 14.90 | 8.81 | 10.09 | 2.24 | 0.42 | 0.13 | 4.39 | 14.50 | 3.17 | 0.00 | 1.50 | 0.50 |
| 2 | 230899 | 19.80 | 44.45 | 20.42 | 19.34 | 18.92 | 22.43 | 9.61 | 13.14 | 3.55 | 0.46 | 0.11 | 6.50 | 13.00 | 1.50 | 0.00 | 0.00 | 1.00 |
| 3 | 270899 | 18.80 | 46.81 | 20.51 | 19.57 | 19.21 | 27.07 | 18.58 | 13.94 | 95.23 | 3.28 | 0.26 | 21.61 | 12.62 | 3.09 | 0.00 | 1.50 | 2.00 |
| 3 | 300899 | 18.40 | 47.83 | 20.60 | 19.80 | 19.50 | 31.72 | 27.55 | 14.74 | 186.91 | 6.09 | 0.41 | 36.72 | 12.24 | 4.68 | 0.00 | 3.00 | 3.00 |
| 6 | 30999 | 19.10 | 46.08 | 19.42 | 19.59 | 19.08 | 27.55 | 40.05 | 13.30 | 98.36 | 8.33 | 0.50 | 33.36 | 31.06 | 9.49 | 0.00 | 4.50 | 4.50 |
| 6 | 60999 | 18.30 | 48.09 | 18.24 | 19.38 | 18.66 | 23.39 | 52.55 | 11.85 | 9.82 | 10.56 | 0.58 | 30.00 | 49.88 | 14.30 | 0.00 | 6.00 | 6.00 |
| 5 | 110999 | 16.50 | 53.34 | 17.87 | 18.75 | 18.27 | 20.82 | 96.76 | 8.97 | 5.57 | 37.56 | 0.38 | 18.84 | 39.94 | 15.65 | 0.00 | 4.50 | 3.00 |
| 3 | 160999 | 15.60 | 56.42 | 17.50 | 18.12 | 17.88 | 18.26 | 140.98 | 6.09 | 1.32 | 64.57 | 0.18 | 7.68 | 30.00 | 17.00 | 0.00 | 3.00 | 0.00 |
| 3 | 190999 | 16.40 | 53.66 | 18.06 | 18.02 | 18.05 | 23.06 | 79.78 | 6.57 | 1.57 | 33.25 | 0.17 | 4.52 | 16.80 | 20.00 | 0.00 | 1.50 | 0.00 |
| 2 | 230999 | 17.00 | 51.77 | 18.62 | 17.92 | 18.22 | 27.87 | 18.58 | 7.05 | 1.82 | 1.94 | 0.16 | 1.35 | 3.60 | 23.00 | 0.00 | 0.00 | 0.00 |
| 2 | 70400 | 62.20 | 15.21 | 5.00 | 5.00 | 5.00 | 9.24 | 10.30 | 10.40 | 0.00 | 0.02 | 0.00 | 8.25 | 4.00 | 5.83 | 0.00 | 3.50 | 0.00 |
| 2 | 110400 | 62.20 | 15.21 | 7.07 | 7.37 | 7.45 | 9.19 | 8.64 | 10.40 | 0.00 | 0.03 | 0.00 | 8.63 | 4.47 | 7.29 | 0.00 | 2.63 | 0.00 |
| 2 | 140400 | 50.20 | 18.88 | 11.22 | 12.12 | 12.35 | 9.09 | 5.32 | 10.40 | 0.00 | 0.07 | 0.00 | 9.39 | 5.43 | 10.21 | 0.00 | 0.88 | 0.00 |
| 2 | 180400 | 42.50 | 22.00 | 13.30 | 14.50 | 14.80 | 9.05 | 3.66 | 10.40 | 0.00 | 0.09 | 0.00 | 9.77 | 5.91 | 11.67 | 0.00 | 0.00 | 0.00 |
| 2 | 210400 | 42.30 | 21.52 | 15.90 | 16.95 | 17.00 | 8.96 | 10.21 | 30.04 | 0.02 | 0.08 | 0.14 | 11.51 | 8.83 | 21.77 | 0.00 | 0.00 | 0.00 |
| 4 | 250400 | 25.90 | 36.49 | 18.50 | 19.40 | 19.20 | 8.86 | 16.75 | 49.67 | 0.05 | 0.06 | 0.27 | 13.25 | 11.75 | 31.88 | 0.00 | 0.00 | 0.00 |
| 4 | 290400 | 34.00 | 27.17 | 17.25 | 19.20 | 18.60 | 8.48 | 10.01 | 32.73 | 0.04 | 0.05 | 1.27 | 19.13 | 16.83 | 36.69 | 0.00 | 0.00 | 0.00 |
| 4 | 20500 | 18.00 | 56.03 | 16.00 | 19.00 | 18.00 | 8.09 | 3.27 | 15.79 | 0.04 | 0.04 | 2.27 | 25.00 | 21.90 | 41.50 | 0.00 | 0.00 | 0.00 |
| 4 | 50500 | 26.10 | 37.12 | 18.00 | 19.50 | 19.50 | 4.82 | 3.27 | 9.44 | 0.03 | 0.03 | 1.20 | 24.00 | 23.85 | 43.03 | 0.00 | 0.00 | 0.00 |
| 4 | 80500 | 22.20 | 44.54 | 20.00 | 20.00 | 21.00 | 1.54 | 3.27 | 3.08 | 0.02 | 0.02 | 0.12 | 23.00 | 25.80 | 44.55 | 0.00 | 0.00 | 0.00 |
| 4 | 110500 | 22.50 | 44.33 | 20.00 | 20.00 | 20.50 | 1.64 | 2.22 | 2.60 | 0.01 | 0.03 | 0.06 | 34.10 | 42.28 | 46.08 | 0.00 | 0.00 | 0.00 |
| 4 | 150500 | 22.30 | 44.00 | 20.00 | 20.00 | 20.00 | 1.73 | 1.16 | 2.12 | 0.00 | 0.03 | 0.00 | 45.20 | 58.75 | 47.60 | 0.00 | 0.00 | 0.00 |
| 4 | 190500 | 13.40 | 80.79 | 20.00 | 19.45 | 19.40 | 2.50 | 1.55 | 5.20 | 0.11 | 0.05 | 0.17 | 58.35 | 74.75 | 76.93 | 0.00 | 0.13 | 0.00 |
| 4 | 220500 | 13.10 | 82.37 | 20.00 | 18.90 | 18.80 | 3.27 | 1.93 | 8.28 | 0.21 | 0.07 | 0.34 | 71.50 | 90.75 | 106.25 | 0.00 | 0.25 | 0.00 |
| 4 | 260500 | 22.40 | 45.63 | 20.50 | 19.75 | 19.50 | 5.85 | 1.65 | 10.05 | 0.30 | 0.06 | 0.30 | 90.22 | 104.62 | 114.65 | 0.05 | 0.25 | 0.13 |
| 4 | 300500 | 22.70 | 45.63 | 21.50 | 21.45 | 20.90 | 11.03 | 1.09 | 13.59 | 0.49 | 0.04 | 0.23 | 127.67 | 132.37 | 131.47 | 0.15 | 0.25 | 0.38 |
| 4 | 40600 | 17.30 | 60.12 | 22.00 | 22.30 | 21.60 | 13.62 | 0.81 | 15.36 | 0.59 | 0.03 | 0.20 | 146.40 | 146.25 | 139.88 | 0.20 | 0.25 | 0.50 |
| 4 | 80600 | 17.30 | 60.40 | 23.20 | 23.25 | 23.00 | 18.80 | 0.95 | 18.89 | 0.74 | 0.03 | 0.13 | 157.20 | 185.55 | 156.69 | 0.27 | 0.27 | 0.75 |
| 4 | 130600 | 20.60 | 49.73 | 24.40 | 24.20 | 24.40 | 23.97 | 1.09 | 22.43 | 0.88 | 0.04 | 0.07 | 168.00 | 224.86 | 173.50 | 0.33 | 0.29 | 1.00 |
| 4 | 170600 | 17.20 | 58.81 | 20.20 | 21.90 | 23.00 | 13.24 | 2.14 | 31.29 | 1.16 | 0.05 | 0.01 | 150.93 | 152.75 | 163.50 | 0.41 | 0.00 | 0.63 |
| 4 | 200600 | 13.60 | 78.58 | 19.10 | 19.60 | 18.40 | 2.50 | 2.98 | 40.14 | 0.29 | 0.07 | 0.01 | 116.81 | 111.78 | 153.50 | 0.57 | 0.44 | 0.25 |
| 4 | 240600 | 13.30 | 80.20 | 19.20 | 19.70 | 18.55 | 1.64 | 2.36 | 21.23 | 0.55 | 0.14 | 0.02 | 99.75 | 102.64 | 116.88 | 0.65 | 0.47 | 0.50 |
| 4 | 270600 | 13.30 | 80.20 | 19.30 | 19.80 | 18.70 | 0.77 | 1.73 | 2.31 | 0.82 | 0.21 | 0.03 | 82.68 | 93.50 | 80.25 | 0.74 | 0.50 | 0.75 |
| 4 | 10700 | 13.30 | 80.20 | 19.30 | 19.55 | 19.00 | 3.80 | 2.79 | 3.90 | 0.69 | 0.19 | 0.05 | 48.56 | 59.25 | 50.23 | 0.91 | 0.75 | 0.68 |
| 4 | 30700 | 13.10 | 80.20 | 19.30 | 19.30 | 19.30 | 6.83 | 3.85 | 5.49 | 0.56 | 0.17 | 0.06 | 31.50 | 25.00 | 20.20 | 1.00 | 1.00 | 0.60 |
| 4 | 70700 | 13.10 | 80.60 | 19.65 | 20.65 | 20.65 | 4.28 | 5.10 | 14.30 | 1.10 | 0.12 | 0.09 | 29.34 | 23.17 | 33.43 | 2.04 | 1.00 | 1.97 |
| 4 | 110700 | 13.10 | 82.37 | 20.00 | 22.00 | 22.00 | 1.73 | 6.35 | 23.10 | 1.64 | 0.07 | 0.13 | 27.18 | 21.33 | 46.67 | 3.07 | 1.00 | 3.33 |
| 4 | 150700 | 32.20 | 29.55 | 20.00 | 21.00 | 22.00 | 2.22 | 4.04 | 13.38 | 1.87 | 0.20 | 0.11 | 25.02 | 19.92 | 46.50 | 4.11 | 1.63 | 3.50 |
| 6 | 180700 | 32.10 | 28.55 | 20.00 | 20.00 | 22.00 | 2.70 | 1.73 | 3.66 | 2.10 | 0.32 | 0.09 | 22.86 | 18.50 | 46.33 | 5.14 | 2.25 | 3.67 |
| 6 | 220700 | 32.30 | 27.98 | 20.25 | 19.75 | 20.75 | 8.90 | 4.13 | 3.94 | 1.89 | 0.28 | 0.14 | 22.31 | 18.92 | 46.24 | 4.36 | 1.79 | 3.75 |
| 4 | 260700 | 26.10 | 34.80 | 20.75 | 19.25 | 19.75 | 21.32 | 8.95 | 4.52 | 1.47 | 0.22 | 0.24 | 21.21 | 19.77 | 46.08 | 2.79 | 0.86 | 3.92 |
| 4 | 300700 | 31.60 | 28.89 | 21.00 | 19.00 | 19.00 | 27.53 | 11.36 | 4.81 | 1.27 | 0.19 | 0.29 | 20.67 | 20.20 | 46.00 | 2.00 | 0.40 | 4.00 |
| 4 | 10800 | 82.00 | 11.31 | 20.70 | 19.65 | 19.65 | 15.60 | 6.16 | 2.99 | 3.28 | 0.00 | 0.22 | 29.17 | 16.60 | 42.00 | 1.50 | 0.60 | 2.17 |
| 4 | 50800 | 100.60 | 9.51 | 20.55 | 19.98 | 19.98 | 9.63 | 3.56 | 2.07 | 3.04 | 0.64 | 0.15 | 33.42 | 16.95 | 40.00 | 1.25 | 0.58 | 1.25 |
| 4 | 80800 | 74.80 | 12.84 | 20.40 | 20.30 | 20.30 | 3.66 | 0.96 | 1.16 | 2.81 | 1.28 | 0.07 | 37.67 | 17.30 | 38.00 | 1.00 | 0.55 | 0.33 |
| 4 | 120800 | 69.00 | 14.44 | 22.70 | 21.75 | 22.15 | 4.34 | 1.64 | 3.66 | 3.97 | 0.75 | 0.33 | 47.20 | 17.65 | 40.10 | 0.95 | 0.53 | 0.27 |
| 4 | 150800 | 68.90 | 14.22 | 25.00 | 23.20 | 24.00 | 5.01 | 2.31 | 6.16 | 5.13 | 0.22 | 0.59 | 56.73 | 18.00 | 42.20 | 0.90 | 0.50 | 0.20 |
| 4 | 180800 | 50.20 | 19.35 | 24.85 | 23.85 | 24.00 | 7.22 | 2.41 | 7.03 | 2.82 | 0.71 | 0.62 | 66.27 | 32.75 | 54.35 | 0.85 | 0.88 | 0.35 |
| 4 | 210800 | 32.10 | 31.31 | 24.70 | 24.50 | 24.00 | 9.43 | 2.50 | 7.89 | 0.52 | 1.21 | 0.64 | 75.80 | 47.50 | 66.50 | 0.80 | 1.25 | 0.50 |
| 4 | 240800 | 42.20 | 23.35 | 23.60 | 23.10 | 22.00 | 9.77 | 2.41 | 7.59 | 0.36 | 3.91 | 0.37 | 67.54 | 46.65 | 75.08 | 0.54 | 0.93 | 0.55 |
| 4 | 290800 | 42.30 | 21.73 | 22.50 | 21.70 | 20.00 | 10.11 | 2.31 | 7.29 | 0.21 | 6.62 | 0.10 | 59.29 | 45.80 | 83.67 | 0.29 | 0.60 | 0.60 |
| 4 | 10900 | 42.40 | 20.51 | 20.85 | 20.30 | 19.35 | 10.44 | 2.22 | 4.42 | 1.99 | 5.38 | 0.37 | 57.46 | 62.07 | 76.67 | 0.29 | 0.30 | 0.63 |
| 4 | 40900 | 32.00 | 26.51 | 19.20 | 18.90 | 18.70 | 10.78 | 2.12 | 1.54 | 3.76 | 4.15 | 0.64 | 55.64 | 78.33 | 69.67 | 0.29 | 0.00 | 0.67 |
| 4 | 80900 | 26.20 | 32.53 | 19.90 | 18.45 | 18.00 | 7.99 | 3.08 | 2.02 | 4.07 | 2.22 | 0.53 | 53.82 | 63.92 | 58.83 | 0.29 | 0.00 | 0.58 |
| 4 | 120900 | 26.50 | 32.53 | 20.60 | 18.00 | 17.30 | 5.20 | 4.04 | 2.50 | 4.38 | 0.30 | 0.42 | 52.00 | 49.50 | 48.00 | 0.29 | 0.00 | 0.50 |
| 4 | 150900 | 22.30 | 38.93 | 17.55 | 16.05 | 15.15 | 5.11 | 2.60 | 1.54 | 2.76 | 1.20 | 0.58 | 53.63 | 43.00 | 41.88 | 0.27 | 0.25 | 0.63 |
| 4 | 190900 | 26.10 | 33.40 | 14.50 | 14.10 | 13.00 | 5.01 | 1.16 | 0.58 | 1.13 | 2.10 | 0.74 | 55.25 | 36.50 | 35.75 | 0.25 | 0.50 | 0.75 |
| 4 | 220900 | 22.00 | 41.11 | 13.55 | 13.80 | 13.05 | 3.28 | 1.55 | 1.16 | 0.97 | 1.80 | 0.62 | 42.88 | 33.13 | 28.38 | 0.13 | 0.25 | 0.63 |
| 4 | 260900 | 25.90 | 36.44 | 12.60 | 13.50 | 13.10 | 1.54 | 1.93 | 1.73 | 0.80 | 1.50 | 0.50 | 30.50 | 29.75 | 21.00 | 0.00 | 0.00 | 0.50 |
| 4 | 300900 | 26.30 | 36.22 | 11.65 | 13.20 | 13.05 | 1.45 | 1.35 | 1.73 | 0.77 | 1.07 | 0.50 | 23.75 | 21.58 | 21.50 | 0.00 | 0.00 | 0.25 |
| 2 | 51000 | 21.00 | 42.20 | 10.70 | 12.90 | 13.00 | 1.35 | 0.77 | 1.73 | 0.75 | 0.63 | 0.50 | 17.00 | 13.40 | 22.00 | 0.00 | 0.00 | 0.00 |
| 1 | 90401 | 25.90 | 34.80 | 9.00 | 6.93 | 7.96 | 17.42 | 1.67 | 9.56 | 0.00 | 0.04 | 0.00 | 0.00 | 0.00 | 0.00 | 0.00 | 0.00 | 0.00 |
| 1 | 130401 | 44.60 | 20.60 | 8.73 | 7.14 | 7.93 | 9.95 | 4.57 | 7.27 | 0.02 | 0.03 | 0.00 | 0.20 | 0.00 | 0.00 | 0.00 | 0.00 | 0.00 |
| 1 | 170401 | 44.10 | 19.98 | 8.45 | 7.35 | 7.90 | 2.49 | 7.46 | 4.97 | 0.04 | 0.01 | 0.00 | 0.40 | 0.00 | 0.00 | 0.00 | 0.00 | 0.00 |
| 1 | 200401 | 44.50 | 20.60 | 7.92 | 7.56 | 7.70 | 4.35 | 6.63 | 8.08 | 0.06 | 0.00 | 0.00 | 0.20 | 0.00 | 0.00 | 0.00 | 0.00 | 0.00 |
| 1 | 230401 | 72.50 | 12.93 | 7.38 | 7.77 | 7.50 | 6.22 | 5.81 | 11.20 | 0.08 | 0.00 | 0.00 | 0.00 | 0.00 | 0.00 | 0.00 | 0.00 | 0.00 |
| 1 | 260401 | 158.30 | 6.54 | 8.66 | 9.51 | 10.52 | 5.18 | 5.75 | 7.93 | 0.05 | 0.01 | 0.01 | 0.00 | 0.00 | 0.00 | 0.00 | 0.00 | 0.00 |
| 1 | 300401 | 152.10 | 6.70 | 9.93 | 11.25 | 13.53 | 4.15 | 5.70 | 4.67 | 0.01 | 0.01 | 0.02 | 0.00 | 0.00 | 0.00 | 0.00 | 0.00 | 0.00 |
| 1 | 40501 | 96.60 | 9.94 | 13.50 | 14.54 | 15.59 | 8.09 | 12.60 | 21.62 | 0.01 | 0.03 | 0.01 | 2.00 | 0.75 | 0.00 | 0.00 | 0.00 | 0.00 |
| 1 | 80501 | 57.60 | 16.29 | 17.06 | 17.83 | 17.64 | 12.03 | 19.49 | 38.57 | 0.00 | 0.05 | 0.00 | 4.00 | 1.50 | 0.00 | 0.00 | 0.00 | 0.00 |
| 2 | 110501 | 51.70 | 18.35 | 17.47 | 17.51 | 17.24 | 9.67 | 14.94 | 26.60 | 0.00 | 0.02 | 0.00 | 10.00 | 9.08 | 1.33 | 0.00 | 0.00 | 0.00 |
| 2 | 150501 | 50.30 | 19.46 | 17.89 | 17.20 | 16.84 | 7.32 | 10.40 | 14.63 | 0.00 | 0.00 | 0.00 | 16.00 | 16.67 | 2.67 | 0.00 | 0.00 | 0.00 |
| 4 | 180501 | 47.10 | 20.08 | 17.89 | 17.19 | 16.70 | 5.58 | 6.16 | 13.86 | 0.00 | 0.00 | 0.00 | 90.00 | 58.33 | 4.33 | 0.25 | 0.00 | 0.00 |
| 4 | 220501 | 42.10 | 21.79 | 17.90 | 17.18 | 16.56 | 3.85 | 1.93 | 13.09 | 0.00 | 0.00 | 0.00 | 164.00 | 100.00 | 6.00 | 0.50 | 0.00 | 0.00 |
| 4 | 250501 | 25.70 | 38.25 | 17.97 | 17.49 | 17.12 | 4.24 | 4.81 | 14.63 | 0.03 | 0.03 | 0.00 | 105.50 | 84.33 | 8.33 | 0.75 | 0.33 | 0.67 |
| 4 | 290501 | 31.20 | 31.09 | 18.04 | 17.80 | 17.67 | 4.62 | 7.70 | 16.17 | 0.05 | 0.05 | 0.00 | 47.00 | 68.67 | 10.67 | 1.00 | 0.67 | 1.33 |
| 4 | 20601 | 25.60 | 38.62 | 18.02 | 16.87 | 16.78 | 3.08 | 5.58 | 20.22 | 0.12 | 0.08 | 0.08 | 32.50 | 36.33 | 11.33 | 0.50 | 0.33 | 0.67 |
| 2 | 60601 | 43.30 | 21.94 | 18.00 | 15.93 | 15.88 | 1.54 | 3.47 | 24.26 | 0.19 | 0.10 | 0.15 | 18.00 | 4.00 | 12.00 | 0.00 | 0.00 | 0.00 |
| 2 | 90601 | 26.30 | 37.12 | 17.21 | 16.27 | 16.14 | 5.97 | 10.59 | 26.38 | 0.10 | 0.06 | 0.15 | 9.00 | 2.00 | 10.33 | 0.00 | 0.00 | 0.00 |
| 1 | 120601 | 31.20 | 30.91 | 16.42 | 16.60 | 16.40 | 10.40 | 17.71 | 28.49 | 0.00 | 0.02 | 0.14 | 0.00 | 0.00 | 8.67 | 0.00 | 0.00 | 0.00 |
| 2 | 160601 | 26.20 | 37.84 | 17.45 | 16.99 | 16.86 | 14.82 | 17.91 | 35.23 | 0.01 | 0.04 | 0.39 | 2.00 | 12.60 | 27.83 | 0.00 | 0.40 | 0.75 |
| 4 | 190601 | 36.10 | 26.84 | 18.48 | 17.39 | 17.31 | 19.25 | 18.10 | 41.97 | 0.01 | 0.06 | 0.63 | 4.00 | 25.20 | 47.00 | 0.00 | 0.80 | 1.50 |
| 4 | 230601 | 38.20 | 24.50 | 19.04 | 18.17 | 18.48 | 10.78 | 10.40 | 37.74 | 0.02 | 0.07 | 0.32 | 65.25 | 46.60 | 49.50 | 1.50 | 1.07 | 0.75 |
| 4 | 270601 | 50.20 | 17.66 | 19.60 | 18.95 | 19.65 | 2.31 | 2.70 | 33.50 | 0.03 | 0.09 | 0.00 | 126.50 | 68.00 | 52.00 | 3.00 | 1.33 | 0.00 |
| 4 | 300601 | 50.30 | 17.27 | 20.56 | 19.38 | 20.11 | 6.55 | 5.01 | 39.47 | 0.18 | 0.18 | 0.04 | 63.25 | 34.00 | 26.00 | 1.50 | 0.67 | 0.00 |
| 1 | 40701 | 50.10 | 17.35 | 21.53 | 19.82 | 20.58 | 10.78 | 7.32 | 45.44 | 0.32 | 0.27 | 0.07 | 0.00 | 0.00 | 0.00 | 0.00 | 0.00 | 0.00 |
| 2 | 80701 | 50.20 | 16.87 | 21.77 | 20.55 | 20.96 | 10.11 | 7.32 | 30.42 | 0.22 | 0.27 | 0.10 | 0.00 | 24.00 | 2.33 | 0.00 | 0.50 | 0.00 |
| 2 | 110701 | 28.10 | 30.75 | 22.02 | 21.28 | 21.34 | 9.43 | 7.32 | 15.40 | 0.11 | 0.26 | 0.13 | 0.00 | 48.00 | 4.67 | 0.00 | 1.00 | 0.00 |
| 2 | 130701 | 26.10 | 33.66 | 22.48 | 22.32 | 22.05 | 8.76 | 17.14 | 27.92 | 0.10 | 0.14 | 0.09 | 2.00 | 25.00 | 3.33 | 0.00 | 0.50 | 0.00 |
| 1 | 160701 | 26.10 | 34.18 | 22.94 | 23.35 | 22.75 | 8.09 | 26.95 | 40.43 | 0.08 | 0.02 | 0.05 | 4.00 | 2.00 | 2.00 | 0.00 | 0.00 | 0.00 |
| 3 | 190701 | 28.10 | 31.80 | 22.35 | 22.52 | 21.41 | 11.17 | 31.96 | 29.46 | 62.34 | 0.20 | 0.03 | 3.67 | 10.00 | 1.00 | 0.00 | 0.00 | 0.00 |
| 3 | 230701 | 50.10 | 17.58 | 21.75 | 21.68 | 20.07 | 14.25 | 36.97 | 18.48 | 124.60 | 0.37 | 0.00 | 3.33 | 18.00 | 0.00 | 0.00 | 0.00 | 0.00 |
| 3 | 260701 | 91.00 | 9.72 | 23.21 | 21.58 | 21.38 | 11.94 | 20.99 | 12.90 | 100.71 | 0.23 | 0.01 | 4.07 | 12.00 | 1.00 | 0.00 | 0.00 | 0.00 |
| 3 | 300701 | 102.80 | 0.89 | 24.67 | 21.47 | 22.70 | 9.63 | 5.01 | 7.32 | 76.83 | 0.10 | 0.03 | 4.80 | 6.00 | 2.00 | 0.00 | 0.00 | 0.00 |
| 1 | 30801 | 101.80 | 9.29 | 23.71 | 21.60 | 22.21 | 6.74 | 5.39 | 7.51 | 38.52 | 0.06 | 0.03 | 5.20 | 6.40 | 1.00 | 0.00 | 0.00 | 0.00 |
| 1 | 60801 | 89.60 | 10.31 | 22.75 | 21.73 | 21.72 | 3.85 | 5.78 | 7.70 | 0.21 | 0.01 | 0.03 | 5.60 | 6.80 | 0.00 | 0.00 | 0.00 | 0.00 |
| 2 | 100801 | 69.30 | 13.16 | 22.43 | 21.07 | 21.46 | 3.47 | 5.97 | 8.09 | 0.31 | 0.08 | 0.09 | 5.80 | 7.40 | 2.00 | 1.00 | 0.00 | 0.00 |
| 2 | 130801 | 57.80 | 15.77 | 22.10 | 20.41 | 21.20 | 3.08 | 6.16 | 8.47 | 0.41 | 0.15 | 0.15 | 6.00 | 8.00 | 4.00 | 2.00 | 0.00 | 0.00 |
| 2 | 180801 | 66.40 | 13.65 | 22.82 | 22.09 | 22.46 | 21.76 | 16.17 | 34.27 | 0.60 | 0.40 | 0.60 | 3.75 | 6.00 | 4.00 | 1.00 | 0.00 | 0.00 |
| 1 | 210801 | 45.10 | 19.82 | 23.53 | 23.77 | 23.72 | 40.43 | 26.18 | 60.07 | 0.80 | 0.66 | 1.05 | 1.50 | 4.00 | 4.00 | 0.00 | 0.00 | 0.00 |
| 2 | 240801 | 35.90 | 25.21 | 23.87 | 23.01 | 23.18 | 25.80 | 16.17 | 46.21 | 3.65 | 0.81 | 0.89 | 0.75 | 18.67 | 22.50 | 0.00 | 0.00 | 0.00 |
| 3 | 270801 | 26.10 | 35.12 | 24.20 | 22.25 | 22.64 | 11.17 | 6.16 | 32.35 | 6.50 | 0.96 | 0.73 | 0.00 | 33.33 | 41.00 | 0.00 | 0.00 | 0.00 |
| 3 | 20901 | 36.30 | 24.97 | 21.35 | 19.98 | 19.52 | 26.38 | 12.13 | 22.91 | 10.71 | 5.47 | 0.66 | 2.00 | 19.17 | 24.50 | 0.25 | 0.00 | 0.00 |
| 3 | 50901 | 34.50 | 26.10 | 18.50 | 17.70 | 16.40 | 41.59 | 18.10 | 13.48 | 14.91 | 9.98 | 0.59 | 4.00 | 5.00 | 8.00 | 0.50 | 0.00 | 0.00 |
| 3 | 70901 | 34.20 | 26.26 | 17.33 | 16.93 | 16.28 | 27.12 | 12.62 | 9.49 | 13.05 | 7.00 | 0.37 | 3.50 | 4.50 | 10.50 | 0.25 | 0.00 | 0.00 |
| 3 | 100901 | 34.40 | 26.17 | 16.15 | 16.15 | 16.15 | 12.65 | 7.15 | 5.50 | 11.19 | 4.03 | 0.15 | 3.00 | 4.00 | 13.00 | 0.00 | 0.00 | 0.00 |
| 3 | 140901 | 34.00 | 26.36 | 15.35 | 15.14 | 14.96 | 8.06 | 5.50 | 6.60 | 16.64 | 2.63 | 0.08 | 2.50 | 4.00 | 7.00 | 0.00 | 0.00 | 0.00 |
| 1 | 170901 | 26.00 | 36.58 | 14.54 | 14.12 | 13.78 | 3.47 | 3.85 | 7.70 | 22.10 | 1.24 | 0.00 | 2.00 | 4.00 | 1.00 | 0.00 | 0.00 | 0.00 |
| 1 | 200901 | 55.50 | 16.89 | 14.55 | 14.36 | 13.99 | 1.74 | 2.27 | 4.03 | 11.37 | 0.73 | 0.01 | 2.36 | 2.50 | 3.63 | 0.00 | 0.00 | 0.00 |
| 1 | 240901 | 65.40 | 13.96 | 14.55 | 14.60 | 14.20 | 0.01 | 0.69 | 0.35 | 0.65 | 0.22 | 0.01 | 2.73 | 1.00 | 6.25 | 0.00 | 0.00 | 0.00 |
| 1 | 290901 | 54.70 | 17.01 | 14.55 | 14.57 | 14.37 | 0.07 | 0.04 | 0.01 | 0.51 | 0.16 | 0.01 | 2.54 | 1.41 | 4.68 | 0.00 | 0.00 | 0.00 |
| 1 | 41001 | 50.50 | 18.90 | 14.54 | 14.51 | 14.43 | 0.04 | 0.01 | 0.01 | 0.10 | 0.05 | 0.00 | 2.18 | 2.25 | 1.56 | 0.00 | 0.00 | 0.00 |
| 1 | 91001 | 47.00 | 20.70 | 14.54 | 14.48 | 14.51 | 0.01 | 0.01 | 0.00 | 0.05 | 0.03 | 0.00 | 2.00 | 2.67 | 0.00 | 0.00 | 0.00 | 0.00 |
| 1 | 230403 | 41.84 | 19.51 | 12.30 | 12.60 | 13.00 | 7.81 | 53.75 | 8.94 | 0.31 | 0.45 | 0.58 | 0.00 | 0.00 | 0.00 | 0.00 | 0.00 | 0.00 |
| 1 | 270403 | 39.47 | 21.97 | 12.81 | 13.18 | 13.94 | 10.47 | 37.61 | 10.88 | 0.42 | 0.32 | 0.70 | 0.00 | 0.00 | 0.00 | 0.00 | 0.00 | 0.00 |
| 1 | 300403 | 40.40 | 22.42 | 13.31 | 13.77 | 14.88 | 13.14 | 21.47 | 12.82 | 0.52 | 0.18 | 0.83 | 0.00 | 0.00 | 0.00 | 0.00 | 0.00 | 0.00 |
| 1 | 40503 | 35.72 | 25.55 | 14.93 | 15.06 | 15.93 | 18.96 | 22.59 | 29.27 | 0.36 | 3.35 | 0.88 | 0.71 | 1.75 | 0.00 | 0.00 | 0.00 | 0.00 |
| 1 | 70503 | 34.61 | 26.37 | 16.55 | 16.36 | 16.98 | 24.78 | 23.71 | 45.73 | 0.19 | 6.51 | 0.93 | 1.43 | 3.50 | 0.00 | 0.00 | 0.00 | 0.00 |
| 6 | 110503 | 33.36 | 27.36 | 16.69 | 17.00 | 17.23 | 15.19 | 26.84 | 48.45 | 0.93 | 3.89 | 1.45 | 16.14 | 8.50 | 2.67 | 0.57 | 6.00 | 10.33 |
| 6 | 170503 | 52.73 | 17.22 | 16.84 | 17.64 | 17.48 | 5.61 | 29.97 | 51.18 | 1.66 | 1.26 | 1.97 | 30.86 | 13.50 | 5.33 | 1.14 | 12.00 | 18.67 |
| 6 | 210503 | 38.76 | 22.88 | 17.13 | 16.82 | 16.30 | 7.85 | 13.78 | 29.80 | 0.67 | 0.18 | 3.02 | 22.62 | 15.26 | 12.86 | 1.51 | 7.37 | 19.57 |
| 6 | 240503 | 54.91 | 16.46 | 18.26 | 19.26 | 19.00 | 8.89 | 12.10 | 30.38 | 1.18 | 1.18 | 2.78 | 18.50 | 17.20 | 105.43 | 1.69 | 6.53 | 18.29 |
| 6 | 280503 | 39.68 | 23.27 | 19.39 | 21.70 | 21.70 | 9.93 | 10.41 | 30.97 | 1.68 | 2.18 | 2.55 | 14.38 | 19.13 | 198.00 | 1.88 | 5.68 | 17.00 |
| 6 | 10603 | 35.12 | 25.68 | 20.37 | 22.40 | 22.25 | 9.13 | 8.17 | 25.82 | 10.01 | 3.87 | 2.03 | 13.37 | 21.07 | 102.67 | 1.58 | 4.84 | 11.50 |
| 6 | 40603 | 32.84 | 27.12 | 21.34 | 23.10 | 22.80 | 8.33 | 5.93 | 20.67 | 18.34 | 5.56 | 1.51 | 12.36 | 23.00 | 7.33 | 1.28 | 4.00 | 6.00 |
| 6 | 70603 | 30.23 | 29.40 | 22.87 | 23.25 | 23.40 | 20.35 | 19.22 | 46.00 | 26.30 | 9.16 | 1.28 | 11.35 | 17.82 | 8.31 | 0.99 | 4.63 | 3.00 |
| 3 | 110603 | 25.49 | 34.44 | 24.40 | 23.40 | 24.00 | 32.36 | 32.52 | 71.34 | 34.26 | 12.76 | 1.04 | 10.34 | 12.63 | 9.29 | 0.69 | 5.26 | 0.00 |
| 3 | 140603 | 25.83 | 33.83 | 23.15 | 22.95 | 22.96 | 34.60 | 37.25 | 56.82 | 29.08 | 10.12 | 1.97 | 6.51 | 8.07 | 5.98 | 0.34 | 2.88 | 0.00 |
| 3 | 180603 | 23.77 | 36.66 | 21.90 | 22.50 | 21.93 | 36.85 | 41.97 | 42.29 | 23.90 | 7.49 | 2.89 | 2.67 | 3.50 | 2.67 | 0.00 | 0.50 | 0.00 |
| 3 | 220603 | 23.96 | 36.46 | 20.77 | 20.89 | 20.30 | 32.60 | 39.94 | 49.13 | 17.36 | 6.61 | 2.70 | 2.33 | 3.25 | 3.33 | 0.00 | 0.63 | 0.00 |
| 3 | 270603 | 20.85 | 41.39 | 19.64 | 19.28 | 18.68 | 28.36 | 37.91 | 55.96 | 10.83 | 5.73 | 2.52 | 1.99 | 3.00 | 4.00 | 0.00 | 0.75 | 0.00 |
| 1 | 20703 | 20.85 | 41.39 | 20.84 | 20.88 | 20.80 | 17.62 | 26.91 | 44.86 | 2.62 | 3.97 | 2.16 | 2.00 | 2.50 | 5.33 | 0.00 | 1.00 | 0.00 |
| 1 | 60703 | 26.82 | 32.57 | 20.25 | 20.38 | 20.27 | 21.55 | 32.04 | 53.48 | 1.97 | 4.05 | 1.73 | 3.00 | 1.75 | 4.00 | 0.00 | 0.50 | 0.00 |
| 1 | 90703 | 25.54 | 34.54 | 19.66 | 19.87 | 19.75 | 25.47 | 37.17 | 62.11 | 1.32 | 4.12 | 1.30 | 4.00 | 1.00 | 2.67 | 0.00 | 0.00 | 0.00 |
| 3 | 120703 | 24.83 | 35.02 | 20.11 | 21.11 | 21.03 | 18.42 | 26.35 | 46.99 | 1.75 | 6.54 | 1.66 | 5.83 | 11.25 | 9.33 | 0.00 | 0.00 | 0.00 |
| 3 | 160703 | 21.60 | 39.65 | 20.56 | 22.34 | 22.30 | 11.37 | 15.54 | 31.88 | 2.18 | 8.97 | 2.02 | 7.67 | 21.50 | 16.00 | 0.00 | 0.00 | 0.00 |
| 3 | 200703 | 20.20 | 41.13 | 21.43 | 22.60 | 23.19 | 17.22 | 19.38 | 43.27 | 3.68 | 6.54 | 4.36 | 6.83 | 14.50 | 23.00 | 0.14 | 0.00 | 0.33 |
| 3 | 230703 | 18.28 | 45.22 | 22.31 | 22.86 | 24.08 | 23.07 | 23.23 | 54.67 | 5.18 | 4.12 | 6.70 | 6.00 | 7.50 | 30.00 | 0.29 | 0.00 | 0.67 |
| 4 | 270703 | 18.33 | 44.86 | 22.53 | 22.92 | 23.53 | 14.66 | 16.74 | 44.86 | 4.49 | 5.74 | 4.13 | 16.50 | 18.75 | 56.67 | 0.64 | 0.00 | 1.67 |
| 4 | 300703 | 25.28 | 32.95 | 22.76 | 22.98 | 22.98 | 6.25 | 10.25 | 35.04 | 3.80 | 7.36 | 1.56 | 27.00 | 30.00 | 83.33 | 1.00 | 0.00 | 2.67 |
| 5 | 30803 | 34.81 | 24.85 | 23.34 | 23.59 | 23.64 | 17.22 | 18.50 | 44.30 | 9.02 | 21.13 | 15.64 | 20.67 | 27.25 | 46.33 | 0.67 | 1.00 | 2.00 |
| 5 | 60803 | 25.71 | 34.06 | 23.91 | 24.20 | 24.30 | 28.20 | 26.75 | 53.55 | 14.24 | 34.89 | 29.72 | 14.33 | 24.50 | 9.33 | 0.33 | 2.00 | 1.33 |
| 5 | 100803 | 20.95 | 41.90 | 23.39 | 23.64 | 23.54 | 28.36 | 31.24 | 50.09 | 15.49 | 41.59 | 26.63 | 12.31 | 57.58 | 25.00 | 0.31 | 3.67 | 2.00 |
| 5 | 130803 | 19.72 | 44.63 | 22.87 | 23.08 | 22.78 | 28.52 | 35.72 | 46.62 | 16.75 | 48.28 | 23.55 | 10.29 | 90.67 | 40.67 | 0.29 | 5.33 | 2.67 |
| 5 | 180803 | 18.92 | 46.40 | 22.36 | 22.44 | 22.26 | 31.75 | 28.79 | 49.31 | 23.02 | 31.60 | 21.80 | 7.50 | 51.39 | 24.26 | 0.14 | 3.72 | 2.05 |
| 5 | 220803 | 19.25 | 45.72 | 21.86 | 21.80 | 21.75 | 34.98 | 21.85 | 52.00 | 29.30 | 14.92 | 20.04 | 4.71 | 12.11 | 7.86 | 0.00 | 2.11 | 1.43 |
| 5 | 250803 | 18.55 | 47.54 | 20.80 | 20.71 | 20.51 | 45.20 | 23.90 | 38.50 | 28.23 | 15.23 | 12.21 | 4.19 | 8.30 | 6.43 | 0.00 | 1.80 | 1.71 |
| 3 | 270803 | 18.44 | 47.72 | 19.73 | 19.62 | 19.27 | 55.43 | 25.95 | 24.99 | 27.17 | 15.53 | 4.38 | 3.67 | 4.50 | 5.00 | 0.00 | 1.50 | 2.00 |
| 3 | 310803 | 19.19 | 45.74 | 18.74 | 18.54 | 18.18 | 43.93 | 25.67 | 31.64 | 15.48 | 16.12 | 3.10 | 3.90 | 4.62 | 8.61 | 0.17 | 0.75 | 1.56 |
| 3 | 30903 | 19.89 | 44.03 | 17.74 | 17.46 | 17.10 | 32.44 | 25.39 | 38.29 | 3.79 | 16.70 | 1.81 | 4.14 | 4.74 | 12.22 | 0.34 | 0.00 | 1.11 |
| 5 | 60903 | 21.24 | 41.24 | 18.49 | 19.55 | 18.75 | 20.95 | 25.11 | 44.94 | 7.14 | 18.78 | 8.97 | 3.28 | 7.11 | 10.00 | 0.17 | 0.53 | 1.11 |
| 5 | 100903 | 20.53 | 42.34 | 19.24 | 21.64 | 20.40 | 9.45 | 24.83 | 51.58 | 10.50 | 20.86 | 16.12 | 2.41 | 9.47 | 7.78 | 0.00 | 1.05 | 1.11 |
| 5 | 140903 | 19.89 | 43.81 | 18.06 | 19.26 | 18.65 | 12.74 | 18.54 | 50.92 | 8.98 | 15.92 | 11.05 | 5.52 | 19.21 | 12.22 | 0.00 | 1.32 | 1.67 |
| 5 | 170903 | 20.53 | 42.55 | 16.89 | 16.88 | 16.90 | 16.02 | 12.25 | 50.26 | 7.47 | 10.99 | 5.98 | 8.62 | 28.95 | 16.67 | 0.00 | 1.58 | 2.22 |
| 5 | 200903 | 19.14 | 45.65 | 17.26 | 17.13 | 17.25 | 21.95 | 10.93 | 35.22 | 8.73 | 9.78 | 3.20 | 12.99 | 24.21 | 31.67 | 0.29 | 2.11 | 4.44 |
| 6 | 240903 | 17.98 | 48.71 | 17.63 | 17.38 | 17.60 | 27.87 | 9.61 | 20.19 | 9.98 | 8.58 | 0.42 | 17.35 | 19.47 | 46.67 | 0.59 | 2.63 | 6.67 |
| 3 | 280903 | 19.89 | 43.38 | 17.23 | 17.13 | 17.00 | 22.99 | 9.05 | 16.34 | 8.23 | 8.09 | 0.34 | 9.12 | 10.79 | 26.11 | 0.29 | 1.32 | 3.33 |
| 1 | 11003 | 23.43 | 37.10 | 16.83 | 16.88 | 16.40 | 18.10 | 8.49 | 12.50 | 6.47 | 7.60 | 0.26 | 0.88 | 2.11 | 5.56 | 0.00 | 0.00 | 0.00 |
| 1 | 210404 | 51.05 | 8.99 | 12.87 | 14.13 | 14.60 | 14.25 | 1.14 | 15.87 | 0.16 | 0.16 | 0.16 | 0.00 | 0.00 | 4.00 | 0.00 | 0.00 | 0.00 |
| 1 | 250404 | 56.09 | 9.70 | 12.83 | 14.09 | 13.75 | 10.58 | 4.90 | 18.66 | 0.12 | 0.12 | 0.12 | 0.00 | 0.00 | 2.00 | 0.00 | 0.00 | 0.00 |
| 1 | 280404 | 72.87 | 8.93 | 12.80 | 14.05 | 12.90 | 6.92 | 8.65 | 21.45 | 0.08 | 0.08 | 0.08 | 0.00 | 0.00 | 0.00 | 0.00 | 0.00 | 0.00 |
| 1 | 20504 | 50.48 | 14.73 | 14.13 | 15.00 | 14.60 | 12.71 | 15.64 | 24.24 | 0.14 | 0.14 | 0.14 | 0.83 | 0.75 | 0.00 | 0.33 | 0.00 | 0.00 |
| 1 | 50504 | 48.31 | 16.42 | 15.46 | 15.94 | 16.30 | 18.50 | 22.63 | 27.03 | 0.21 | 0.21 | 0.21 | 1.67 | 1.50 | 0.00 | 0.67 | 0.00 | 0.00 |
| 2 | 90504 | 41.47 | 20.55 | 15.51 | 15.73 | 15.99 | 16.82 | 17.32 | 22.97 | 0.19 | 0.19 | 0.19 | 4.83 | 6.42 | 0.33 | 1.50 | 4.67 | 1.33 |
| 6 | 120504 | 44.24 | 19.89 | 15.56 | 15.52 | 15.68 | 15.14 | 12.02 | 18.90 | 0.17 | 0.17 | 0.17 | 8.00 | 11.33 | 0.67 | 2.33 | 9.33 | 2.67 |
| 6 | 150504 | 45.37 | 19.92 | 17.69 | 15.97 | 15.95 | 10.65 | 12.10 | 21.59 | 0.12 | 0.12 | 0.12 | 13.42 | 14.42 | 2.33 | 2.92 | 7.67 | 3.33 |
| 6 | 190504 | 46.28 | 19.72 | 19.82 | 16.42 | 16.23 | 6.17 | 12.18 | 24.27 | 0.07 | 0.07 | 0.07 | 18.83 | 17.50 | 4.00 | 3.50 | 6.00 | 4.00 |
| 6 | 220504 | 39.82 | 22.64 | 17.07 | 15.36 | 15.15 | 4.49 | 10.09 | 26.95 | 0.05 | 0.05 | 0.05 | 24.25 | 11.75 | 5.67 | 4.08 | 3.25 | 11.00 |
| 6 | 260504 | 34.19 | 25.87 | 14.31 | 14.30 | 14.08 | 2.80 | 8.01 | 29.64 | 0.03 | 0.03 | 0.03 | 29.67 | 6.00 | 7.33 | 4.67 | 0.50 | 18.00 |
| 4 | 290504 | 32.67 | 26.87 | 15.21 | 15.99 | 16.37 | 4.49 | 5.37 | 23.23 | 0.08 | 0.08 | 0.08 | 17.40 | 5.00 | 10.67 | 2.40 | 0.25 | 9.50 |
| 2 | 20604 | 29.55 | 29.71 | 16.10 | 17.68 | 18.67 | 6.17 | 2.72 | 16.82 | 0.12 | 0.12 | 0.12 | 5.13 | 4.00 | 14.00 | 0.14 | 0.00 | 1.00 |
| 4 | 50604 | 27.95 | 31.41 | 17.32 | 18.29 | 19.03 | 4.75 | 10.25 | 14.74 | 0.16 | 0.16 | 0.16 | 35.42 | 20.50 | 8.33 | 1.36 | 2.50 | 0.83 |
| 4 | 90604 | 28.07 | 31.20 | 18.54 | 18.90 | 19.40 | 3.32 | 17.78 | 12.66 | 0.21 | 0.21 | 0.21 | 65.71 | 37.00 | 2.67 | 2.57 | 5.00 | 0.67 |
| 6 | 120604 | 27.46 | 32.12 | 18.62 | 18.94 | 19.09 | 2.97 | 13.78 | 23.39 | 0.54 | 0.54 | 0.54 | 48.14 | 39.50 | 21.33 | 2.14 | 5.75 | 5.33 |
| 6 | 160604 | 27.65 | 32.29 | 18.70 | 18.98 | 18.78 | 2.62 | 9.77 | 34.12 | 0.87 | 0.87 | 0.87 | 30.57 | 42.00 | 40.00 | 1.71 | 6.50 | 10.00 |
| 6 | 190604 | 26.13 | 34.18 | 18.70 | 18.75 | 18.80 | 3.36 | 9.45 | 25.23 | 0.56 | 0.56 | 0.56 | 22.57 | 30.00 | 28.18 | 2.43 | 6.25 | 7.50 |
| 6 | 230604 | 26.00 | 7.55 | 18.70 | 18.52 | 18.83 | 4.11 | 9.13 | 16.34 | 0.24 | 0.24 | 0.24 | 14.57 | 18.00 | 16.35 | 3.14 | 6.00 | 5.00 |
| 6 | 260604 | 24.44 | 35.92 | 18.98 | 18.76 | 18.95 | 9.91 | 15.22 | 11.13 | 0.65 | 0.65 | 0.65 | 23.86 | 19.50 | 46.68 | 3.29 | 7.00 | 5.00 |
| 6 | 290604 | 23.31 | 37.30 | 19.25 | 19.00 | 19.07 | 15.70 | 21.31 | 5.93 | 1.07 | 1.07 | 1.07 | 33.14 | 21.00 | 77.00 | 3.43 | 8.00 | 5.00 |
| 6 | 30704 | 22.48 | 38.58 | 19.36 | 19.19 | 19.36 | 11.59 | 18.36 | 8.49 | 1.12 | 1.12 | 1.12 | 34.14 | 22.50 | 68.00 | 2.71 | 7.25 | 3.75 |
| 6 | 70704 | 22.65 | 37.90 | 19.47 | 19.38 | 19.66 | 7.48 | 15.42 | 11.05 | 1.17 | 1.17 | 1.17 | 35.14 | 24.00 | 59.00 | 2.00 | 6.50 | 2.50 |
| 6 | 100704 | 20.90 | 41.19 | 19.58 | 19.57 | 19.95 | 6.07 | 12.12 | 13.62 | 1.48 | 1.48 | 1.48 | 28.14 | 17.50 | 46.75 | 1.43 | 3.75 | 5.75 |
| 6 | 140704 | 20.95 | 41.38 | 19.69 | 19.76 | 20.25 | 4.67 | 8.81 | 16.18 | 1.79 | 1.79 | 1.79 | 21.14 | 11.00 | 34.50 | 0.86 | 1.00 | 9.00 |
| 6 | 160704 | 21.29 | 40.82 | 19.47 | 19.62 | 19.83 | 5.79 | 8.09 | 17.14 | 2.85 | 2.85 | 2.85 | 19.77 | 18.00 | 27.58 | 2.23 | 0.75 | 9.17 |
| 6 | 180704 | 20.65 | 42.20 | 19.25 | 19.48 | 19.40 | 6.92 | 7.37 | 18.10 | 3.90 | 3.90 | 3.90 | 18.40 | 25.00 | 20.67 | 3.60 | 0.50 | 9.33 |
| 6 | 210704 | 26.78 | 32.62 | 20.89 | 22.52 | 22.08 | 17.99 | 11.85 | 11.21 | 19.74 | 19.74 | 19.74 | 15.33 | 35.00 | 15.50 | 2.33 | 3.50 | 8.50 |
| 6 | 250704 | 21.73 | 40.90 | 21.21 | 22.21 | 22.06 | 17.69 | 14.10 | 16.02 | 16.74 | 16.74 | 16.74 | 24.83 | 34.75 | 50.42 | 1.50 | 2.50 | 7.25 |
| 6 | 280704 | 21.48 | 41.28 | 21.53 | 21.90 | 22.05 | 17.38 | 16.34 | 20.83 | 13.74 | 13.74 | 13.74 | 34.33 | 34.50 | 85.33 | 0.67 | 1.50 | 6.00 |
| 6 | 10804 | 21.01 | 41.58 | 21.28 | 21.73 | 21.73 | 18.69 | 20.83 | 24.23 | 10.74 | 10.74 | 10.74 | 30.88 | 32.00 | 63.00 | 0.90 | 2.50 | 9.00 |
| 6 | 40804 | 21.77 | 40.03 | 21.04 | 21.56 | 21.40 | 20.00 | 25.31 | 27.63 | 7.74 | 7.74 | 7.74 | 27.43 | 29.50 | 40.67 | 1.14 | 3.50 | 12.00 |
| 6 | 70804 | 20.20 | 43.14 | 21.48 | 22.20 | 22.23 | 19.81 | 17.70 | 31.04 | 6.18 | 6.18 | 6.18 | 23.05 | 38.50 | 34.33 | 1.07 | 2.00 | 7.33 |
| 5 | 110804 | 19.78 | 43.53 | 21.93 | 22.84 | 23.05 | 19.62 | 10.09 | 34.44 | 4.62 | 4.62 | 4.62 | 18.67 | 47.50 | 28.00 | 1.00 | 0.50 | 2.67 |
| 5 | 140804 | 23.09 | 37.18 | 21.86 | 22.23 | 22.41 | 27.75 | 11.37 | 24.67 | 18.74 | 18.74 | 18.74 | 17.00 | 36.25 | 21.33 | 0.50 | 1.50 | 2.00 |
| 5 | 180804 | 21.88 | 39.33 | 21.80 | 21.62 | 21.78 | 35.88 | 12.66 | 14.90 | 32.87 | 32.87 | 32.87 | 15.33 | 25.00 | 14.67 | 0.00 | 2.50 | 1.33 |
| 5 | 210804 | 20.48 | 42.04 | 21.69 | 21.41 | 21.41 | 38.13 | 33.56 | 14.42 | 26.11 | 26.11 | 26.11 | 27.10 | 85.50 | 20.08 | 0.00 | 2.25 | 1.67 |
| 5 | 250804 | 19.95 | 42.83 | 21.58 | 21.20 | 21.04 | 40.37 | 54.47 | 13.94 | 19.35 | 19.35 | 19.35 | 38.86 | 146.00 | 25.50 | 0.00 | 2.00 | 2.00 |
| 5 | 280804 | 18.55 | 45.70 | 20.75 | 20.48 | 20.26 | 33.36 | 42.01 | 11.29 | 24.55 | 24.55 | 24.55 | 30.93 | 82.00 | 28.08 | 0.33 | 1.50 | 3.33 |
| 5 | 10904 | 19.89 | 42.20 | 19.93 | 19.75 | 19.48 | 26.35 | 29.56 | 8.65 | 29.76 | 29.76 | 29.76 | 23.00 | 18.00 | 30.67 | 0.67 | 1.00 | 4.67 |
| 5 | 50904 | 17.92 | 46.14 | 20.24 | 19.88 | 19.74 | 27.94 | 17.10 | 5.77 | 22.93 | 22.93 | 22.93 | 34.30 | 31.00 | 30.83 | 0.33 | 1.75 | 4.33 |
| 5 | 80904 | 17.92 | 45.54 | 20.55 | 20.00 | 20.00 | 29.53 | 4.65 | 2.88 | 16.10 | 16.10 | 16.10 | 45.60 | 44.00 | 31.00 | 0.00 | 2.50 | 4.00 |
| 5 | 110904 | 17.75 | 44.91 | 19.32 | 18.88 | 18.77 | 31.59 | 4.01 | 4.49 | 17.59 | 17.59 | 17.59 | 36.13 | 44.00 | 33.17 | 0.33 | 2.50 | 6.00 |
| 6 | 140904 | 17.01 | 44.80 | 18.09 | 17.76 | 17.53 | 33.64 | 3.36 | 6.09 | 19.08 | 19.08 | 19.08 | 26.67 | 44.00 | 35.33 | 0.67 | 2.50 | 8.00 |
| 5 | 180904 | 17.65 | 40.32 | 16.94 | 16.73 | 16.07 | 27.10 | 5.37 | 5.82 | 14.48 | 14.48 | 14.48 | 27.33 | 37.50 | 20.17 | 0.33 | 1.25 | 4.50 |
| 5 | 220904 | 17.07 | 40.29 | 15.80 | 15.70 | 14.60 | 20.56 | 7.37 | 5.55 | 9.88 | 9.88 | 9.88 | 28.00 | 31.00 | 5.00 | 0.00 | 0.00 | 1.00 |
| 5 | 260904 | 21.41 | 31.93 | 15.11 | 14.96 | 14.10 | 17.85 | 6.09 | 3.50 | 8.56 | 8.56 | 8.56 | 26.00 | 19.33 | 5.17 | 0.00 | 0.00 | 0.50 |
| 3 | 290904 | 22.00 | 31.25 | 14.43 | 14.23 | 13.60 | 15.14 | 4.81 | 1.44 | 7.24 | 7.24 | 7.24 | 24.00 | 7.67 | 5.33 | 0.00 | 0.00 | 0.00 |
| 1 | 130405 | 41.67 | 19.21 | 11.00 | 11.80 | 11.80 | 10.57 | 11.37 | 5.29 | 0.00 | 0.00 | 0.00 | 0.00 | 0.00 | 0.00 | 0.00 | 0.00 | 1.00 |
| 1 | 170405 | 37.42 | 21.25 | 11.01 | 12.80 | 12.80 | 10.89 | 16.26 | 3.28 | 0.00 | 0.00 | 0.00 | 0.17 | 0.00 | 0.00 | 0.00 | 0.00 | 0.50 |
| 1 | 200405 | 35.55 | 22.31 | 11.01 | 13.80 | 13.80 | 11.21 | 21.15 | 1.28 | 0.00 | 0.00 | 0.00 | 0.33 | 0.00 | 0.00 | 0.00 | 0.00 | 0.00 |
| 1 | 230405 | 33.43 | 23.79 | 11.52 | 13.09 | 13.09 | 15.70 | 17.30 | 1.60 | 0.00 | 0.00 | 0.00 | 1.67 | 1.25 | 0.00 | 0.00 | 0.00 | 0.00 |
| 1 | 270405 | 33.36 | 24.21 | 12.03 | 12.38 | 12.38 | 20.19 | 13.46 | 1.92 | 0.00 | 0.00 | 0.00 | 3.00 | 2.50 | 0.00 | 0.00 | 0.00 | 0.00 |
| 1 | 300405 | 37.34 | 22.45 | 13.26 | 13.57 | 13.57 | 16.50 | 16.66 | 10.25 | 0.00 | 0.00 | 0.00 | 5.67 | 1.75 | 0.00 | 0.50 | 0.75 | 0.25 |
| 1 | 40505 | 35.72 | 24.51 | 14.49 | 14.76 | 14.76 | 12.82 | 19.86 | 18.58 | 0.00 | 0.00 | 0.00 | 8.33 | 1.00 | 0.00 | 1.00 | 1.50 | 0.50 |
| 1 | 70505 | 107.38 | 8.50 | 13.56 | 13.58 | 13.58 | 10.73 | 18.66 | 10.01 | 0.00 | 0.00 | 0.00 | 4.17 | 0.50 | 0.00 | 0.50 | 0.75 | 0.25 |
| 1 | 100505 | 90.37 | 10.13 | 12.64 | 12.40 | 12.40 | 8.65 | 17.46 | 1.44 | 0.00 | 0.00 | 0.00 | 0.00 | 0.00 | 0.00 | 0.00 | 0.00 | 0.00 |
| 1 | 130505 | 78.69 | 11.49 | 13.18 | 13.18 | 13.18 | 7.05 | 14.30 | 4.65 | 0.00 | 0.00 | 0.00 | 0.00 | 0.00 | 0.00 | 0.00 | 0.00 | 0.00 |
| 1 | 170505 | 54.48 | 16.15 | 13.73 | 13.96 | 13.96 | 5.45 | 11.13 | 7.85 | 0.01 | 0.00 | 0.02 | 0.00 | 0.00 | 0.00 | 0.00 | 0.00 | 0.00 |
| 4 | 200505 | 56.82 | 15.64 | 15.99 | 14.74 | 14.74 | 6.81 | 7.97 | 31.73 | 0.40 | 0.01 | 0.03 | 47.60 | 27.50 | 7.00 | 0.40 | 0.25 | 1.33 |
| 4 | 240505 | 45.13 | 19.17 | 18.26 | 15.52 | 15.52 | 8.17 | 4.81 | 55.61 | 0.90 | 0.01 | 0.05 | 95.20 | 55.00 | 14.00 | 0.80 | 0.50 | 2.67 |
| 4 | 280505 | 37.20 | 22.97 | 19.14 | 16.76 | 16.76 | 5.29 | 2.72 | 29.09 | 1.00 | 0.30 | 0.07 | 67.00 | 137.50 | 65.00 | 1.20 | 1.58 | 3.00 |
| 4 | 10605 | 33.50 | 25.25 | 20.01 | 18.00 | 18.00 | 2.40 | 0.64 | 2.56 | 1.40 | 0.30 | 0.09 | 38.80 | 220.00 | 116.00 | 1.60 | 2.67 | 3.33 |
| 4 | 40605 | 30.66 | 27.51 | 19.11 | 17.89 | 17.89 | 3.36 | 1.12 | 3.04 | 1.75 | 0.16 | 0.10 | 24.40 | 115.33 | 62.50 | 1.20 | 1.67 | 2.17 |
| 2 | 80605 | 29.42 | 28.74 | 18.20 | 17.78 | 17.78 | 4.33 | 1.60 | 3.52 | 2.11 | 0.02 | 0.10 | 10.00 | 10.67 | 9.00 | 0.80 | 0.67 | 1.00 |
| 2 | 110605 | 28.62 | 28.88 | 21.01 | 19.20 | 19.20 | 9.93 | 7.41 | 11.77 | 1.14 | 0.04 | 0.05 | 9.80 | 14.83 | 8.83 | 0.60 | 0.33 | 0.83 |
| 2 | 150605 | 27.16 | 30.05 | 23.83 | 20.63 | 20.63 | 15.54 | 13.22 | 20.03 | 0.17 | 0.07 | 0.00 | 9.60 | 19.00 | 8.67 | 0.40 | 0.00 | 0.67 |
| 4 | 190605 | 25.71 | 32.40 | 22.02 | 20.78 | 20.78 | 9.61 | 8.13 | 16.34 | 0.17 | 0.11 | 0.21 | 19.66 | 38.00 | 13.00 | 0.20 | 0.00 | 0.67 |
| 4 | 220605 | 24.88 | 33.74 | 20.21 | 20.94 | 20.94 | 3.68 | 3.04 | 12.66 | 0.17 | 0.15 | 0.42 | 29.71 | 57.00 | 17.33 | 0.00 | 0.00 | 0.67 |
| 4 | 260605 | 22.14 | 37.90 | 21.10 | 21.09 | 21.09 | 5.61 | 2.16 | 8.81 | 2.56 | 0.69 | 0.24 | 35.16 | 36.56 | 44.44 | 0.00 | 0.00 | 0.33 |
| 4 | 280605 | 22.42 | 37.54 | 21.99 | 21.24 | 21.24 | 7.53 | 1.28 | 4.97 | 4.94 | 1.23 | 0.06 | 40.61 | 16.11 | 71.54 | 0.00 | 0.00 | 0.00 |
| 5 | 20705 | 19.67 | 42.67 | 21.89 | 22.27 | 22.27 | 8.49 | 6.41 | 30.12 | 5.04 | 5.87 | 56.30 | 62.02 | 14.06 | 47.77 | 0.00 | 0.00 | 0.00 |
| 5 | 60705 | 18.48 | 44.97 | 21.79 | 23.30 | 23.30 | 9.45 | 11.53 | 55.27 | 5.14 | 10.51 | 112.55 | 83.43 | 12.00 | 24.00 | 0.00 | 0.00 | 0.00 |
| 5 | 100705 | 18.18 | 45.48 | 22.89 | 23.25 | 23.25 | 19.70 | 14.58 | 51.66 | 21.09 | 25.39 | 91.49 | 64.57 | 61.00 | 21.33 | 1.14 | 1.00 | 1.33 |
| 5 | 130705 | 17.54 | 46.89 | 24.00 | 23.20 | 23.20 | 29.96 | 17.62 | 48.06 | 37.03 | 40.27 | 70.44 | 45.71 | 110.00 | 18.67 | 2.29 | 2.00 | 2.67 |
| 5 | 190705 | 19.61 | 41.72 | 23.80 | 23.48 | 23.48 | 37.57 | 18.10 | 26.35 | 45.89 | 37.05 | 38.43 | 27.86 | 72.00 | 13.33 | 1.64 | 1.00 | 1.33 |
| 5 | 260705 | 20.42 | 40.18 | 23.61 | 23.76 | 23.76 | 45.18 | 18.58 | 4.65 | 54.75 | 33.83 | 6.42 | 10.00 | 34.00 | 8.00 | 1.00 | 0.00 | 0.00 |
| 5 | 300705 | 18.97 | 43.24 | 23.41 | 24.04 | 24.04 | 28.52 | 14.82 | 11.29 | 43.16 | 22.88 | 3.54 | 32.14 | 45.00 | 13.33 | 0.50 | 1.00 | 0.33 |
| 3 | 30805 | 19.89 | 41.67 | 23.21 | 24.32 | 24.32 | 11.85 | 11.05 | 17.94 | 31.57 | 11.92 | 0.65 | 54.29 | 56.00 | 18.67 | 0.00 | 2.00 | 0.67 |
| 5 | 80805 | 21.48 | 38.99 | 21.97 | 22.82 | 22.82 | 12.13 | 11.61 | 17.34 | 25.63 | 12.68 | 1.72 | 43.29 | 44.50 | 18.00 | 0.00 | 2.50 | 0.50 |
| 5 | 130805 | 20.42 | 41.11 | 19.51 | 19.84 | 19.84 | 12.69 | 12.73 | 16.14 | 13.76 | 14.22 | 3.86 | 21.29 | 21.50 | 16.66 | 0.00 | 3.50 | 0.17 |
| 5 | 170805 | 19.14 | 43.86 | 18.28 | 18.35 | 18.35 | 12.98 | 13.30 | 15.54 | 7.83 | 14.99 | 4.94 | 10.29 | 10.00 | 16.00 | 0.00 | 4.00 | 0.00 |
| 5 | 200805 | 18.50 | 45.49 | 18.71 | 19.23 | 19.23 | 33.72 | 13.86 | 37.57 | 8.88 | 87.78 | 14.56 | 9.14 | 18.00 | 9.33 | 0.00 | 2.00 | 0.00 |
| 5 | 230805 | 17.70 | 47.31 | 19.14 | 20.10 | 20.10 | 54.47 | 14.42 | 59.59 | 9.94 | 160.56 | 24.17 | 8.00 | 26.00 | 2.67 | 0.00 | 0.00 | 0.00 |
| 5 | 260805 | 19.03 | 43.90 | 20.11 | 20.26 | 20.26 | 46.14 | 10.09 | 37.89 | 8.11 | 85.50 | 13.69 | 40.57 | 20.00 | 4.00 | 0.00 | 0.00 | 0.00 |
| 3 | 300805 | 20.36 | 41.12 | 21.08 | 20.42 | 20.42 | 37.81 | 5.77 | 16.18 | 6.27 | 10.43 | 3.20 | 73.14 | 14.00 | 5.33 | 0.00 | 0.00 | 0.00 |
| 3 | 30905 | 18.87 | 44.15 | 21.00 | 20.39 | 20.39 | 26.99 | 5.05 | 15.94 | 4.43 | 6.95 | 2.87 | 58.86 | 33.00 | 10.67 | 0.00 | 0.00 | 0.00 |
| 4 | 70905 | 15.85 | 52.29 | 20.93 | 20.36 | 20.36 | 16.18 | 4.33 | 15.70 | 2.59 | 3.47 | 2.54 | 44.57 | 52.00 | 16.00 | 0.00 | 0.00 | 0.00 |
| 3 | 110905 | 15.45 | 52.95 | 20.93 | 20.33 | 20.33 | 24.03 | 7.05 | 11.53 | 36.87 | 9.55 | 2.21 | 23.95 | 36.00 | 14.00 | 0.00 | 0.00 | 0.00 |
| 3 | 150905 | 17.01 | 47.84 | 20.93 | 20.30 | 20.30 | 31.88 | 9.77 | 7.37 | 71.15 | 15.63 | 1.88 | 3.33 | 20.00 | 12.00 | 0.00 | 0.00 | 0.00 |
| 5 | 180905 | 17.70 | 45.76 | 19.23 | 18.91 | 18.91 | 28.12 | 15.80 | 28.06 | 46.26 | 26.46 | 38.71 | 9.67 | 10.00 | 38.00 | 0.00 | 0.00 | 0.00 |
| 5 | 210905 | 18.87 | 42.71 | 17.54 | 17.52 | 17.52 | 24.35 | 21.83 | 48.75 | 21.36 | 37.30 | 75.54 | 16.00 | 0.00 | 64.00 | 0.00 | 0.00 | 0.00 |
| 5 | 250905 | 18.18 | 44.09 | 17.51 | 17.33 | 17.33 | 32.04 | 13.43 | 27.18 | 23.52 | 24.96 | 38.04 | 22.67 | 0.00 | 36.50 | 0.00 | 0.00 | 0.00 |
| 3 | 280905 | 18.08 | 44.11 | 17.47 | 17.14 | 17.14 | 39.73 | 5.03 | 5.61 | 25.68 | 12.62 | 0.54 | 29.33 | 0.00 | 9.00 | 0.00 | 0.00 | 0.00 |
| 3 | 21005 | 20.20 | 39.47 | 16.75 | 16.56 | 16.56 | 27.97 | 24.14 | 4.01 | 22.93 | 10.69 | 0.86 | 21.33 | 28.00 | 27.00 | 0.00 | 0.00 | 0.00 |
| 3 | 51005 | 20.31 | 39.26 | 16.03 | 15.98 | 15.98 | 16.22 | 43.25 | 2.40 | 20.18 | 8.76 | 1.18 | 13.33 | 56.00 | 45.00 | 0.00 | 0.00 | 0.00 |
| 3 | 91005 | 21.88 | 36.53 | 16.84 | 16.90 | 16.90 | 12.68 | 25.31 | 5.33 | 17.39 | 6.98 | 0.94 | 19.47 | 32.00 | 34.00 | 0.00 | 0.00 | 0.00 |
| 3 | 121005 | 22.59 | 35.57 | 17.64 | 17.82 | 17.82 | 9.13 | 7.37 | 8.25 | 14.60 | 5.20 | 0.70 | 25.60 | 8.00 | 23.00 | 0.00 | 0.00 | 0.00 |
| 1 | 260406 | 49.24 | 18.49 | 14.00 | 14.20 | 14.50 | 60.65 | 8.81 | 42.99 | 0.22 | 0.33 | 0.05 | 0.29 | 0.80 | 0.00 | 0.29 | 0.40 | 0.00 |
| 1 | 270406 | 47.83 | 18.54 | 14.00 | 14.50 | 14.70 | 18.77 | 10.53 | 15.06 | 0.22 | 0.33 | 0.05 | 0.57 | 4.00 | 0.00 | 0.00 | 0.00 | 0.00 |
| 1 | 280406 | 45.17 | 18.72 | 14.10 | 15.00 | 15.40 | 14.88 | 13.02 | 23.50 | 0.28 | 0.33 | 0.44 | 0.86 | 11.50 | 0.00 | 0.00 | 0.00 | 0.00 |
| 1 | 290406 | 44.51 | 18.48 | 14.10 | 16.08 | 16.50 | 9.84 | 10.07 | 59.27 | 0.22 | 0.73 | 0.09 | 0.00 | 12.00 | 0.00 | 0.00 | 0.00 | 0.00 |
| 1 | 10506 | 44.72 | 18.82 | 14.34 | 15.20 | 15.18 | 11.21 | 4.01 | 17.62 | 0.04 | 0.10 | 0.15 | 0.00 | 13.00 | 0.00 | 0.00 | 0.00 | 0.00 |
| 1 | 40506 | 51.98 | 16.93 | 14.49 | 16.24 | 14.95 | 9.29 | 12.82 | 21.47 | 0.09 | 0.18 | 0.21 | 0.00 | 21.00 | 1.33 | 0.00 | 0.00 | 0.00 |
| 2 | 70506 | 44.89 | 20.09 | 15.39 | 16.73 | 15.96 | 14.26 | 15.62 | 23.31 | 0.08 | 0.15 | 0.12 | 2.12 | 21.06 | 0.67 | 0.15 | 0.00 | 0.00 |
| 2 | 100506 | 39.18 | 23.13 | 16.29 | 17.22 | 16.98 | 19.22 | 18.42 | 25.15 | 0.08 | 0.11 | 0.02 | 4.24 | 21.11 | 0.00 | 0.30 | 0.00 | 0.00 |
| 2 | 130506 | 35.45 | 25.95 | 17.00 | 17.53 | 17.68 | 12.58 | 12.58 | 19.70 | 0.37 | 0.12 | 0.14 | 9.85 | 15.56 | 1.15 | 0.30 | 0.00 | 0.38 |
| 2 | 170506 | 33.16 | 27.19 | 17.71 | 17.84 | 18.38 | 5.93 | 6.73 | 14.26 | 0.66 | 0.13 | 0.25 | 15.45 | 10.00 | 2.31 | 0.30 | 0.00 | 0.77 |
| 2 | 220506 | 33.36 | 26.83 | 17.68 | 17.82 | 18.15 | 5.29 | 5.89 | 12.90 | 0.54 | 0.13 | 0.20 | 15.61 | 20.50 | 5.19 | 0.38 | 0.00 | 0.58 |
| 4 | 290506 | 31.66 | 28.28 | 17.61 | 17.77 | 17.70 | 4.01 | 4.21 | 10.17 | 0.29 | 0.14 | 0.11 | 15.91 | 41.50 | 10.96 | 0.53 | 0.00 | 0.19 |
| 4 | 60606 | 31.92 | 27.78 | 17.57 | 17.74 | 17.48 | 3.36 | 3.36 | 8.81 | 0.17 | 0.14 | 0.06 | 16.06 | 52.00 | 13.85 | 0.61 | 0.00 | 0.00 |
| 4 | 110606 | 28.93 | 30.57 | 19.13 | 19.35 | 19.55 | 3.08 | 4.61 | 8.93 | 0.24 | 0.14 | 0.07 | 61.71 | 45.67 | 25.38 | 0.54 | 0.00 | 0.00 |
| 4 | 160606 | 24.56 | 35.57 | 22.24 | 22.57 | 23.70 | 2.52 | 7.09 | 9.17 | 0.39 | 0.13 | 0.10 | 153.02 | 33.00 | 48.46 | 0.40 | 0.00 | 0.00 |
| 4 | 210606 | 24.74 | 35.75 | 23.80 | 24.18 | 25.78 | 2.24 | 8.33 | 9.29 | 0.47 | 0.12 | 0.12 | 198.67 | 26.67 | 60.00 | 0.33 | 0.00 | 0.00 |
| 4 | 250606 | 22.84 | 38.73 | 24.96 | 25.14 | 26.67 | 8.86 | 10.81 | 20.35 | 1.11 | 2.77 | 0.30 | 120.67 | 22.67 | 37.00 | 0.17 | 0.00 | 0.00 |
| 4 | 280606 | 22.48 | 39.25 | 26.11 | 26.10 | 27.57 | 15.49 | 13.30 | 31.40 | 1.76 | 5.41 | 0.49 | 42.67 | 18.67 | 14.00 | 0.00 | 0.00 | 0.00 |
| 4 | 20706 | 22.48 | 38.96 | 25.48 | 25.62 | 26.42 | 9.18 | 13.06 | 20.59 | 3.24 | 7.13 | 0.68 | 21.33 | 32.00 | 11.00 | 0.00 | 0.00 | 0.00 |
| 3 | 50706 | 19.67 | 43.88 | 24.84 | 25.14 | 25.28 | 2.88 | 12.82 | 9.77 | 4.72 | 8.86 | 0.87 | 0.00 | 45.33 | 8.00 | 0.00 | 0.00 | 0.00 |
| 3 | 90706 | 16.28 | 52.74 | 26.59 | 26.84 | 26.68 | 7.29 | 12.02 | 10.49 | 5.36 | 9.48 | 1.22 | 0.00 | 34.67 | 15.50 | 0.00 | 0.00 | 1.00 |
| 3 | 120706 | 15.27 | 55.81 | 28.34 | 28.54 | 28.08 | 11.69 | 11.21 | 11.21 | 6.01 | 10.09 | 1.56 | 0.00 | 24.00 | 23.00 | 0.00 | 0.00 | 2.00 |
| 3 | 160706 | 15.03 | 56.14 | 27.15 | 27.23 | 27.03 | 39.65 | 21.43 | 13.30 | 23.34 | 14.17 | 2.42 | 1.60 | 14.67 | 19.00 | 0.00 | 0.00 | 1.50 |
| 3 | 190706 | 13.27 | 62.60 | 25.97 | 25.92 | 25.98 | 67.60 | 31.64 | 15.38 | 40.67 | 18.26 | 3.27 | 3.20 | 5.33 | 15.00 | 0.00 | 0.00 | 1.00 |
| 5 | 220706 | 12.61 | 65.20 | 26.70 | 26.74 | 27.04 | 39.09 | 25.67 | 15.22 | 25.68 | 18.95 | 13.34 | 9.60 | 13.33 | 8.50 | 0.00 | 0.00 | 0.50 |
| 5 | 260706 | 12.66 | 63.47 | 27.43 | 27.56 | 28.10 | 10.57 | 19.70 | 15.06 | 10.69 | 19.64 | 23.40 | 16.00 | 21.33 | 2.00 | 0.00 | 0.00 | 0.00 |
| 5 | 30806 | 13.61 | 56.92 | 26.81 | 26.96 | 27.25 | 11.29 | 16.18 | 16.42 | 13.17 | 15.68 | 29.54 | 16.00 | 21.33 | 5.00 | 0.00 | 0.00 | 0.00 |
| 5 | 90806 | 16.26 | 47.61 | 26.19 | 26.36 | 26.40 | 12.02 | 12.66 | 17.78 | 15.66 | 11.73 | 35.67 | 16.00 | 21.33 | 8.00 | 0.00 | 0.00 | 0.00 |
| 5 | 120806 | 16.95 | 45.81 | 25.08 | 25.09 | 25.00 | 19.30 | 14.10 | 16.58 | 13.54 | 12.11 | 24.80 | 17.09 | 33.33 | 4.00 | 0.00 | 0.00 | 0.00 |
| 5 | 160806 | 16.15 | 47.71 | 23.97 | 23.82 | 23.60 | 26.59 | 15.54 | 15.38 | 11.42 | 12.49 | 13.93 | 18.18 | 45.33 | 0.00 | 0.00 | 0.00 | 0.00 |
| 5 | 210806 | 16.60 | 46.28 | 23.33 | 23.51 | 23.41 | 20.59 | 14.74 | 19.74 | 12.76 | 12.38 | 10.65 | 16.44 | 41.67 | 5.00 | 0.00 | 0.05 | 0.30 |
| 5 | 270806 | 17.52 | 43.73 | 22.69 | 23.19 | 23.21 | 14.58 | 13.94 | 24.11 | 14.10 | 12.26 | 7.37 | 14.69 | 38.00 | 10.00 | 0.00 | 0.20 | 0.50 |
| 5 | 30906 | 20.96 | 36.96 | 22.37 | 23.03 | 23.12 | 11.57 | 13.54 | 26.29 | 14.78 | 12.21 | 5.73 | 13.82 | 36.17 | 12.50 | 0.00 | 0.40 | 1.00 |
| 3 | 80906 | 20.01 | 38.92 | 21.73 | 22.72 | 22.92 | 5.57 | 12.74 | 30.66 | 16.12 | 12.09 | 2.45 | 12.07 | 32.50 | 17.50 | 0.00 | 0.60 | 2.00 |
| 3 | 120906 | 18.73 | 41.57 | 21.41 | 22.56 | 22.83 | 2.56 | 12.34 | 32.84 | 16.79 | 12.03 | 0.80 | 11.20 | 30.67 | 20.00 | 0.00 | 0.67 | 2.00 |
| 3 | 170906 | 16.72 | 45.71 | 20.71 | 21.88 | 21.46 | 2.80 | 11.53 | 19.14 | 8.94 | 7.89 | 0.52 | 8.93 | 15.67 | 26.50 | 0.67 | 0.33 | 1.00 |
| 2 | 200906 | 16.66 | 45.63 | 20.00 | 21.20 | 20.10 | 3.04 | 10.73 | 5.45 | 1.10 | 3.75 | 0.24 | 6.67 | 0.67 | 33.00 | 1.33 | 0.00 | 0.00 |
| 4 | 230906 | 16.09 | 47.11 | 21.20 | 22.08 | 21.44 | 4.97 | 9.93 | 4.97 | 6.45 | 2.50 | 1.89 | 11.33 | 26.33 | 17.50 | 0.67 | 0.00 | 0.00 |
| 3 | 270906 | 15.97 | 46.94 | 22.40 | 22.95 | 22.78 | 6.89 | 9.13 | 4.49 | 11.80 | 1.24 | 3.54 | 16.00 | 52.00 | 2.00 | 0.00 | 0.00 | 0.00 |
| 3 | 51006 | 20.77 | 36.11 | 21.43 | 21.56 | 21.24 | 8.37 | 7.57 | 3.66 | 14.32 | 1.03 | 2.89 | 14.00 | 41.00 | 3.25 | 0.00 | 0.00 | 0.00 |
| 3 | 111006 | 20.13 | 37.26 | 19.48 | 18.79 | 18.16 | 11.33 | 4.45 | 2.02 | 19.37 | 0.61 | 1.59 | 10.00 | 19.00 | 5.75 | 0.00 | 0.00 | 0.00 |
| 3 | 181006 | 21.08 | 35.76 | 18.50 | 17.40 | 16.63 | 12.82 | 2.88 | 1.20 | 21.89 | 0.40 | 0.94 | 8.00 | 8.00 | 7.00 | 0.00 | 0.00 | 0.00 |
| 1 | 180407 | 22.00 | 41.08 | 12.43 | 10.70 | 13.50 | 7.53 | 8.33 | 10.73 | 0.20 | 0.00 | 0.06 | 2.29 | 2.00 | 0.67 | 0.00 | 0.50 | 0.00 |
| 1 | 220407 | 49.70 | 17.66 | 12.33 | 11.72 | 13.31 | 5.93 | 6.97 | 7.53 | 0.11 | 0.00 | 0.03 | 2.48 | 3.33 | 1.33 | 0.00 | 0.25 | 0.00 |
| 1 | 260407 | 12.70 | 68.62 | 12.23 | 12.74 | 13.13 | 4.33 | 5.61 | 4.33 | 0.02 | 0.00 | 0.00 | 2.67 | 4.67 | 2.00 | 0.00 | 0.00 | 0.00 |
| 2 | 10507 | 12.70 | 70.48 | 12.91 | 13.83 | 14.05 | 18.90 | 13.94 | 19.30 | 0.02 | 0.00 | 0.01 | 4.67 | 4.67 | 6.50 | 0.17 | 0.00 | 1.00 |
| 2 | 40507 | 12.70 | 71.34 | 13.60 | 14.92 | 14.98 | 33.48 | 22.27 | 34.28 | 0.01 | 0.00 | 0.03 | 6.67 | 4.67 | 11.00 | 0.33 | 0.00 | 2.00 |
| 2 | 70507 | 12.70 | 71.86 | 22.78 | 22.84 | 23.15 | 2.56 | 14.90 | 28.76 | 0.03 | 0.00 | 0.00 | 0.00 | 16.00 | 12.00 | 0.00 | 0.00 | 1.00 |
| 4 | 120507 | 21.50 | 42.69 | 19.41 | 19.28 | 19.71 | 2.92 | 11.21 | 25.99 | 0.02 | 0.00 | 0.00 | 18.00 | 19.67 | 13.50 | 0.00 | 0.00 | 1.00 |
| 4 | 170507 | 21.70 | 41.75 | 16.05 | 15.72 | 16.28 | 3.28 | 7.53 | 23.23 | 0.01 | 0.00 | 0.00 | 36.00 | 23.33 | 15.00 | 0.00 | 0.00 | 1.00 |
| 4 | 200507 | 25.00 | 35.72 | 17.39 | 18.10 | 18.84 | 2.92 | 6.73 | 17.06 | 0.01 | 0.00 | 0.09 | 27.33 | 84.00 | 17.00 | 0.00 | 0.67 | 2.00 |
| 4 | 240507 | 16.70 | 53.21 | 18.73 | 20.48 | 21.40 | 2.56 | 5.93 | 10.89 | 0.02 | 0.00 | 0.17 | 18.67 | 144.67 | 19.00 | 0.00 | 1.33 | 3.00 |
| 4 | 260507 | 16.70 | 53.09 | 19.59 | 20.80 | 21.20 | 3.04 | 4.33 | 16.09 | 0.04 | 0.00 | 0.45 | 35.00 | 94.33 | 20.50 | 0.17 | 1.00 | 2.50 |
| 4 | 310507 | 16.70 | 52.82 | 20.46 | 21.12 | 21.00 | 3.52 | 2.72 | 21.28 | 0.07 | 0.00 | 0.73 | 51.33 | 44.00 | 22.00 | 0.33 | 0.67 | 2.00 |
| 4 | 40607 | 14.70 | 60.31 | 20.93 | 21.66 | 21.47 | 6.69 | 15.22 | 24.57 | 0.23 | 0.22 | 0.57 | 47.00 | 55.17 | 21.00 | 0.33 | 0.67 | 1.50 |
| 4 | 90607 | 14.70 | 60.89 | 21.88 | 22.73 | 22.41 | 13.02 | 40.21 | 31.15 | 0.55 | 0.65 | 0.27 | 38.33 | 77.50 | 19.00 | 0.33 | 0.67 | 0.50 |
| 4 | 140607 | 14.70 | 61.19 | 22.35 | 23.26 | 22.88 | 16.18 | 52.71 | 34.44 | 0.70 | 0.87 | 0.12 | 34.00 | 88.67 | 18.00 | 0.33 | 0.67 | 0.00 |
| 4 | 180607 | 14.70 | 60.89 | 21.79 | 21.99 | 21.66 | 11.29 | 28.68 | 21.95 | 0.73 | 1.75 | 0.08 | 31.67 | 84.33 | 33.00 | 0.33 | 0.67 | 0.00 |
| 4 | 220607 | 14.70 | 61.34 | 21.23 | 20.71 | 20.44 | 6.41 | 4.65 | 9.45 | 0.76 | 2.63 | 0.05 | 29.33 | 80.00 | 48.00 | 0.33 | 0.67 | 0.00 |
| 4 | 260607 | 16.70 | 53.86 | 20.94 | 20.39 | 20.13 | 14.54 | 3.48 | 7.09 | 9.11 | 1.97 | 0.03 | 23.75 | 73.25 | 44.25 | 0.33 | 0.58 | 0.13 |
| 4 | 300607 | 16.70 | 53.73 | 20.38 | 19.75 | 19.52 | 30.80 | 1.16 | 2.36 | 25.81 | 0.66 | 0.01 | 12.58 | 59.75 | 36.75 | 0.33 | 0.42 | 0.38 |
| 4 | 50707 | 16.70 | 53.60 | 20.10 | 19.44 | 19.22 | 38.93 | 0.00 | 0.00 | 34.17 | 0.00 | 0.00 | 7.00 | 53.00 | 33.00 | 0.33 | 0.33 | 0.50 |
| 3 | 80707 | 14.70 | 60.75 | 19.23 | 18.80 | 18.61 | 36.65 | 7.26 | 16.86 | 17.33 | 3.36 | 7.78 | 12.83 | 39.50 | 25.50 | 0.17 | 0.17 | 0.75 |
| 5 | 120707 | 16.70 | 53.34 | 18.36 | 18.16 | 18.00 | 34.36 | 14.53 | 33.71 | 0.49 | 6.71 | 15.57 | 18.67 | 26.00 | 18.00 | 0.00 | 0.00 | 1.00 |
| 5 | 160707 | 16.70 | 53.21 | 19.74 | 20.14 | 20.15 | 32.08 | 26.65 | 26.63 | 10.29 | 12.32 | 8.07 | 30.17 | 26.33 | 22.00 | 0.00 | 0.00 | 0.50 |
| 3 | 190707 | 16.70 | 53.21 | 21.13 | 22.12 | 22.30 | 29.80 | 38.77 | 19.54 | 20.09 | 17.92 | 0.57 | 41.67 | 26.67 | 26.00 | 0.00 | 0.00 | 0.00 |
| 3 | 220707 | 16.70 | 53.34 | 21.09 | 21.58 | 21.90 | 43.89 | 27.39 | 11.29 | 27.73 | 15.05 | 0.68 | 26.00 | 22.00 | 37.50 | 0.00 | 0.00 | 0.00 |
| 3 | 260707 | 16.70 | 52.96 | 21.06 | 21.04 | 21.50 | 57.99 | 16.02 | 3.04 | 35.37 | 12.18 | 0.80 | 10.33 | 17.33 | 49.00 | 0.00 | 0.00 | 0.00 |
| 5 | 290707 | 12.70 | 68.95 | 20.29 | 20.24 | 20.34 | 45.10 | 15.06 | 3.20 | 24.16 | 11.01 | 3.13 | 12.33 | 14.67 | 40.00 | 0.33 | 0.67 | 1.00 |
| 5 | 20807 | 14.70 | 58.99 | 19.51 | 19.44 | 19.18 | 32.20 | 14.10 | 3.36 | 12.96 | 9.84 | 5.45 | 14.33 | 12.00 | 31.00 | 0.67 | 1.33 | 2.00 |
| 5 | 60807 | 12.70 | 67.44 | 19.87 | 20.73 | 20.81 | 18.18 | 14.74 | 21.31 | 9.30 | 8.95 | 12.39 | 14.00 | 20.00 | 30.50 | 0.33 | 1.33 | 1.00 |
| 5 | 90807 | 12.70 | 67.10 | 20.23 | 22.02 | 22.45 | 4.17 | 15.38 | 39.25 | 5.65 | 8.07 | 19.33 | 13.67 | 28.00 | 30.00 | 0.00 | 1.33 | 0.00 |
| 5 | 120807 | 12.70 | 66.59 | 21.14 | 21.89 | 22.61 | 11.45 | 11.29 | 34.52 | 18.39 | 9.76 | 11.14 | 17.00 | 16.00 | 21.50 | 0.33 | 2.67 | 0.00 |
| 5 | 160807 | 12.70 | 66.43 | 22.06 | 21.76 | 22.78 | 18.74 | 7.21 | 29.80 | 31.12 | 11.45 | 2.96 | 20.33 | 4.00 | 13.00 | 0.67 | 4.00 | 0.00 |
| 5 | 200807 | 12.70 | 65.93 | 22.32 | 21.99 | 22.73 | 30.04 | 30.20 | 24.19 | 23.01 | 18.89 | 3.54 | 24.57 | 17.33 | 14.00 | 0.33 | 2.00 | 2.00 |
| 5 | 230807 | 12.70 | 65.76 | 22.59 | 22.22 | 22.68 | 41.33 | 53.19 | 18.58 | 14.90 | 26.33 | 4.13 | 28.80 | 30.67 | 15.00 | 0.00 | 0.00 | 4.00 |
| 5 | 260807 | 12.70 | 65.43 | 21.50 | 21.12 | 21.29 | 49.74 | 43.41 | 14.98 | 18.91 | 17.30 | 3.66 | 22.40 | 23.33 | 11.00 | 0.00 | 0.00 | 2.00 |
| 3 | 300807 | 12.70 | 64.76 | 20.41 | 20.02 | 19.90 | 58.15 | 33.64 | 11.37 | 22.91 | 8.27 | 3.20 | 16.00 | 16.00 | 7.00 | 0.00 | 0.00 | 0.00 |
| 3 | 50907 | 12.60 | 64.11 | 19.72 | 19.34 | 19.20 | 58.39 | 25.23 | 8.60 | 23.00 | 6.20 | 2.42 | 17.33 | 14.67 | 9.75 | 0.17 | 0.00 | 0.00 |
| 3 | 90907 | 12.60 | 64.44 | 18.33 | 17.99 | 17.81 | 58.85 | 8.41 | 3.06 | 23.17 | 2.07 | 0.86 | 20.00 | 12.00 | 15.25 | 0.50 | 0.00 | 0.00 |
| 4 | 130907 | 12.60 | 65.27 | 17.64 | 17.31 | 17.11 | 59.09 | 0.00 | 0.29 | 23.26 | 0.00 | 0.08 | 21.33 | 10.67 | 18.00 | 0.67 | 0.00 | 0.00 |
| 4 | 170907 | 15.90 | 51.86 | 16.25 | 15.96 | 15.72 | 42.78 | 0.00 | 0.59 | 16.84 | 0.00 | 0.17 | 15.47 | 17.00 | 13.50 | 0.33 | 0.00 | 0.50 |
| 4 | 200907 | 12.60 | 65.44 | 14.86 | 14.60 | 14.33 | 26.47 | 0.00 | 0.89 | 10.42 | 0.00 | 0.25 | 9.60 | 23.33 | 9.00 | 0.00 | 0.00 | 1.00 |
| 2 | 230907 | 12.70 | 64.22 | 15.05 | 14.83 | 14.70 | 26.97 | 0.00 | 0.55 | 10.61 | 0.00 | 0.16 | 7.20 | 11.67 | 4.50 | 0.00 | 0.00 | 0.50 |
| 1 | 270907 | 12.70 | 64.14 | 15.24 | 15.06 | 15.08 | 27.46 | 0.00 | 0.21 | 10.81 | 0.00 | 0.06 | 4.80 | 0.00 | 0.00 | 0.00 | 0.00 | 0.00 |
| 2 | 80508 | 31.59 | 27.31 | 14.80 | 15.50 | 15.90 | 1.76 | 2.24 | 7.69 | 0.00 | 0.00 | 0.00 | 48.00 | 14.36 | 1.00 | 0.40 | 0.00 | 0.00 |
| 4 | 110508 | 31.72 | 26.93 | 15.75 | 16.60 | 17.15 | 2.56 | 1.52 | 8.57 | 0.00 | 0.00 | 0.00 | 72.40 | 73.52 | 25.50 | 0.40 | 0.33 | 2.00 |
| 4 | 150508 | 34.75 | 24.77 | 16.70 | 17.70 | 18.40 | 3.36 | 0.80 | 9.45 | 0.00 | 0.00 | 0.00 | 96.80 | 132.67 | 50.00 | 0.40 | 0.67 | 4.00 |
| 4 | 200508 | 32.41 | 26.63 | 16.72 | 17.52 | 17.90 | 4.16 | 0.60 | 14.25 | 0.00 | 0.00 | 0.00 | 81.90 | 106.50 | 42.16 | 1.00 | 1.17 | 3.00 |
| 4 | 240508 | 30.10 | 28.88 | 16.77 | 17.17 | 16.90 | 5.76 | 0.60 | 23.87 | 0.00 | 0.00 | 0.00 | 52.10 | 54.16 | 26.50 | 2.20 | 2.17 | 1.00 |
| 4 | 290508 | 42.55 | 21.34 | 16.80 | 17.00 | 16.40 | 6.57 | 0.80 | 28.68 | 0.00 | 0.00 | 0.00 | 37.20 | 28.00 | 18.67 | 2.80 | 2.67 | 0.00 |
| 4 | 20608 | 111.70 | 8.65 | 17.95 | 18.60 | 18.90 | 4.65 | 1.20 | 14.66 | 0.00 | 0.00 | 0.00 | 25.00 | 19.67 | 18.00 | 1.60 | 1.67 | 0.00 |
| 2 | 50608 | 95.06 | 10.27 | 19.10 | 20.20 | 21.40 | 2.72 | 1.60 | 0.64 | 0.00 | 0.00 | 0.00 | 12.80 | 11.33 | 17.33 | 0.40 | 0.67 | 0.00 |
| 4 | 80608 | 72.39 | 12.68 | 20.15 | 20.65 | 21.40 | 4.45 | 3.44 | 3.88 | 1.22 | 1.22 | 1.22 | 61.54 | 11.42 | 27.92 | 1.63 | 0.33 | 0.75 |
| 4 | 120608 | 83.82 | 11.05 | 21.20 | 21.10 | 21.40 | 6.17 | 5.29 | 7.13 | 2.43 | 2.43 | 2.43 | 110.29 | 11.50 | 38.50 | 2.86 | 0.00 | 1.50 |
| 6 | 160608 | 78.16 | 11.62 | 21.15 | 20.80 | 20.95 | 9.17 | 2.88 | 6.05 | 6.64 | 6.64 | 6.64 | 70.14 | 52.25 | 103.25 | 1.93 | 1.50 | 1.08 |
| 5 | 190608 | 50.79 | 17.92 | 21.10 | 20.50 | 20.50 | 12.18 | 0.48 | 4.97 | 10.85 | 10.85 | 10.85 | 30.00 | 93.00 | 168.00 | 1.00 | 3.00 | 0.67 |
| 6 | 230608 | 37.57 | 24.00 | 21.75 | 21.05 | 21.50 | 8.33 | 2.48 | 3.36 | 7.88 | 7.88 | 7.88 | 25.00 | 54.50 | 134.00 | 1.00 | 2.50 | 2.33 |
| 6 | 270608 | 41.76 | 21.07 | 22.40 | 21.60 | 22.50 | 4.49 | 4.49 | 1.76 | 4.92 | 4.92 | 4.92 | 20.00 | 16.00 | 100.00 | 1.00 | 2.00 | 4.00 |
| 5 | 300608 | 40.03 | 21.61 | 22.15 | 22.60 | 22.70 | 15.30 | 28.60 | 6.17 | 14.51 | 14.51 | 14.51 | 29.00 | 29.75 | 72.33 | 0.83 | 2.00 | 3.00 |
| 5 | 30708 | 35.51 | 24.05 | 21.90 | 23.60 | 22.90 | 26.11 | 52.71 | 10.57 | 24.09 | 24.09 | 24.09 | 38.00 | 43.50 | 44.67 | 0.67 | 2.00 | 2.00 |
| 5 | 60708 | 33.71 | 25.28 | 21.55 | 22.40 | 22.10 | 35.56 | 46.94 | 6.81 | 29.18 | 29.18 | 29.18 | 43.33 | 38.75 | 101.33 | 1.00 | 1.50 | 3.33 |
| 5 | 100708 | 32.81 | 25.84 | 21.20 | 21.20 | 21.30 | 45.02 | 41.17 | 3.04 | 34.28 | 34.28 | 34.28 | 48.67 | 34.00 | 158.00 | 1.33 | 1.00 | 4.67 |
| 5 | 130708 | 29.94 | 28.46 | 21.35 | 21.15 | 21.40 | 41.89 | 31.72 | 3.12 | 29.15 | 29.15 | 29.15 | 46.00 | 34.00 | 130.00 | 1.17 | 0.75 | 3.67 |
| 5 | 170708 | 27.32 | 31.74 | 21.50 | 21.10 | 21.50 | 38.77 | 22.27 | 3.20 | 24.03 | 24.03 | 24.03 | 43.33 | 34.00 | 102.00 | 1.00 | 0.50 | 2.67 |
| 5 | 190708 | 34.85 | 24.88 | 21.40 | 21.05 | 21.30 | 76.42 | 24.03 | 5.13 | 39.89 | 39.89 | 39.89 | 67.67 | 39.50 | 101.67 | 1.67 | 4.00 | 1.67 |
| 5 | 220708 | 35.70 | 24.23 | 21.30 | 21.00 | 21.10 | 114.06 | 25.79 | 7.05 | 55.75 | 55.75 | 55.75 | 92.00 | 45.00 | 101.33 | 2.33 | 7.50 | 0.67 |
| 6 | 260708 | 30.22 | 28.20 | 22.25 | 23.05 | 22.90 | 64.24 | 29.40 | 29.16 | 36.58 | 36.58 | 36.58 | 52.00 | 35.00 | 109.33 | 1.67 | 5.00 | 7.67 |
| 6 | 300708 | 29.26 | 28.46 | 23.20 | 25.10 | 24.70 | 14.42 | 33.00 | 51.26 | 17.40 | 17.40 | 17.40 | 12.00 | 25.00 | 117.33 | 1.00 | 2.50 | 14.67 |
| 6 | 30808 | 33.59 | 24.80 | 22.85 | 23.40 | 23.45 | 58.31 | 56.39 | 37.17 | 43.12 | 43.12 | 43.12 | 42.00 | 17.50 | 86.67 | 0.83 | 3.75 | 12.67 |
| 5 | 70808 | 48.65 | 17.61 | 22.50 | 21.70 | 22.20 | 102.21 | 79.78 | 23.07 | 68.84 | 68.84 | 68.84 | 72.00 | 10.00 | 56.00 | 0.67 | 5.00 | 10.67 |
| 5 | 100808 | 38.84 | 22.38 | 22.35 | 21.75 | 22.30 | 75.93 | 49.50 | 12.90 | 61.82 | 61.82 | 61.82 | 62.67 | 13.00 | 33.00 | 1.33 | 3.50 | 5.67 |
| 5 | 140808 | 34.48 | 25.28 | 22.20 | 21.80 | 22.40 | 49.66 | 19.22 | 2.72 | 54.81 | 54.81 | 54.81 | 53.33 | 16.00 | 10.00 | 2.00 | 2.00 | 0.67 |
| 5 | 170808 | 46.28 | 18.83 | 21.65 | 21.25 | 21.65 | 35.24 | 12.66 | 7.53 | 34.92 | 34.92 | 34.92 | 60.67 | 30.50 | 18.67 | 2.00 | 3.50 | 3.67 |
| 6 | 210808 | 38.54 | 22.33 | 21.10 | 20.70 | 20.90 | 20.83 | 6.09 | 12.34 | 15.03 | 15.03 | 15.03 | 68.00 | 45.00 | 27.33 | 2.00 | 5.00 | 6.67 |
| 6 | 250808 | 31.10 | 27.19 | 20.75 | 20.40 | 20.60 | 17.46 | 7.21 | 10.49 | 10.50 | 10.50 | 10.50 | 39.67 | 31.50 | 26.67 | 2.17 | 4.25 | 6.33 |
| 6 | 280808 | 30.23 | 27.63 | 20.40 | 20.10 | 20.30 | 14.10 | 8.33 | 8.65 | 5.97 | 5.97 | 5.97 | 11.33 | 18.00 | 26.00 | 2.33 | 3.50 | 6.00 |
| 6 | 10908 | 27.46 | 30.18 | 20.30 | 19.90 | 20.15 | 41.33 | 13.94 | 5.69 | 17.69 | 17.69 | 17.69 | 7.00 | 16.25 | 26.67 | 4.50 | 3.25 | 6.00 |
| 6 | 40908 | 27.46 | 29.95 | 20.20 | 19.70 | 20.00 | 68.57 | 19.54 | 2.72 | 29.40 | 29.40 | 29.40 | 2.67 | 14.50 | 27.33 | 6.67 | 3.00 | 6.00 |
| 6 | 70908 | 25.40 | 32.13 | 20.20 | 19.70 | 19.90 | 56.71 | 17.46 | 2.48 | 19.70 | 19.70 | 19.70 | 46.67 | 42.75 | 46.67 | 5.17 | 3.75 | 4.67 |
| 6 | 110908 | 28.07 | 28.85 | 20.20 | 19.70 | 19.80 | 44.86 | 15.38 | 2.24 | 10.00 | 10.00 | 10.00 | 90.67 | 71.00 | 66.00 | 3.67 | 4.50 | 3.33 |
| 6 | 140908 | 25.34 | 32.04 | 17.65 | 16.95 | 16.45 | 38.53 | 32.12 | 6.57 | 6.31 | 6.31 | 6.31 | 60.33 | 64.50 | 48.67 | 2.17 | 3.75 | 2.00 |
| 6 | 180908 | 30.62 | 27.28 | 15.10 | 14.20 | 13.10 | 32.20 | 48.86 | 10.89 | 2.62 | 2.62 | 2.62 | 30.00 | 58.00 | 31.33 | 0.67 | 3.00 | 0.67 |

Data base of SOM including: Subcl – the number of the subcluster; Code of date - the code for each term consists of two digits for the day, two digits for the month and two digits for the year of sampling, e.g., 190799 = 19th of July 1999; Inflow (m^3^ s^-1^); Ret time - retention time (days); TRtem - temperature (˚C) in Tresta; BRtem - temperature (˚C) in Bronisławów; ZAtem - temperature (˚C) in Zarzęcin; TRchl – concentration of chlorophyll *a* (µg dm^-3^) in Tresta; BRchl – concentration of chlorophyll *a* (µg dm^-3^) in Bronisławów; ZAchl – concentration of chlorophyll *a* (µg dm^-3^) in Zarzęcin; TRcya – biomass of cyanobacteria (mg dm^-3^) in Tresta; BRcya – biomass of cyanobacteria (mg dm^-3^) in Bronisławów; ZAcya – biomass of cyanobacteria (mg dm^-3^) in Zarzęcin; TRdap - density of *D. longispina* (ind dm^-3^) in Tresta; BRdap - density of *D. longispina* (ind dm^-3^) in Bronisławów; ZAdap - density of *D. longispina* (ind dm^-3^) in Zarzęcin; TRlep - density of *L. kindtii* (ind dm^-3^) in Tresta; BRlep - density of *L. kindtii* (ind dm^-3^) in Bronisławów; ZAlep - density of *L. kindtii* (ind dm^-3^) in Zarzęcin.
