# Supplementary material for: Long-Term Patterns in the Population Dynamics of Daphnia longispina, Leptodora kindtii and Cyanobacteria in a Shallow Reservoir: A Self-Organising Map (SOM) Approach
Source: PLoS One. 2015 Dec 3;10(12):e0144109. doi: 10.1371/journal.pone.0144109 (PMC4669109; doi:10.1371/journal.pone.0144109)
Supplement: S2 Table — (DOCX) [file pone.0144109.s003.docx]

**S2 Table. Self-organizing maps of different calculated sizes, quantization and topographic errors and the number of empty neurons (which were not appreciated).**

| Size | Quantization error | Topographic error | No. of empty neurons |
| --- | --- | --- | --- |
| 4 × 4 | 0.447 | 0.010 | 0 |
| 5 × 4 | 0.433 | 0.002 | 0 |
| 6 × 4 (final choice) | 0.410 | 0.007 | 0 |
| 7 × 5 | 0.385 | 0.007 | 0 |
| 8 × 6 | 0.359 | 0.015 | 0 |
| 9 × 7 | 0.362 | 0.007 | 1 |
| 10 × 8 | 0.350 | 0.029 | 4 |
| 11 × 9 | 0.332 | 0.007 | 7 |
| 12 × 9 | 0.329 | 0.017 | 8 |
